# Tofu and fish oil independently modulate serum lipid profiles in rats: Analyses of 10 class lipoprotein profiles and the global hepatic transcriptome

**DOI:** 10.1371/journal.pone.0210950

**Published:** 2019-01-17

**Authors:** Yoko Takahashi, Tomokazu Konishi, Kohji Yamaki

**Affiliations:** 1 Food Research Institute, National Agriculture and Food Research Organization (NARO), Tsukuba, Ibaraki, Japan; 2 Graduate School of Bioresource Sciences, Akita Prefectural University, Akita, Japan; University of Illinois, UNITED STATES

## Abstract

Soy protein and fish oil are food components that decrease the risk of cardiovascular disease. Previous studies demonstrated that these food components reduced serum cholesterol levels and suppressed hepatic lipogenesis. However, the underlying mechanisms of action of these food components remain unclear. Ten classes of serum lipoprotein profiles showed that dietary tofu, a soybean curd, suppressed cholesterol absorption, while fish oil reduced most of the lipoprotein classes in rats. Tofu and fish oil both halved the level of the lipoprotein class LAC1 (LDL-anti-protease complex), a 15-nm LDL-anti-protease complex, which is speculated to be a cause of atherosclerosis. Moreover, a global transcriptome analysis revealed that tofu inhibited the mRNA expression of genes involved in hepatic lipogenesis, while fish oil stimulated that of genes related to fatty acid degradation. Therefore, tofu and fish oil independently regulate lipid metabolism. The decrease observed in LAC1 may have been due to reduced cholesterol absorption in the tofu diet group and the interference of lipogenesis via the activation of polyunsaturated fatty acid detoxification in the fish oil group.

## Introduction

Cardiovascular disease, a leading cause of death worldwide, is associated with atheroma. Its major direct cause appears to be the rupture of atherosclerotic plaques [1], and focal arterial inflammatory activity is one of its most prominent characteristics [2]. Continuous exposure to inflammation or other types of endothelial activation increases vascular permeability, thereby allowing excess lipid infiltration in the intima and promoting transmigration on immune cells and monocytes, resulting in the formation of an atherosclerotic plaque [3]. The formation of fragile and leaky vessels that invade the expanding intima concomitantly contributes to enlarging the necrotic core, which increases the vulnerability of the plaque [3]. When plaques rupture, prothrombotic material is exposed to the coagulation system with the ensuing inhibition of blood flow [1]. Since atherosclerotic plaques contain a fibrous cap overlying a lipid-rich necrotic core consisting of oxidized lipoproteins, cholesterol crystals, and cellular debris [4], high plasma LDL-cholesterol concentrations are considered to contribute to the progression of atherosclerosis [5]. However, previous cohort studies did not find a correlation between LDL-cholesterol levels and the incidence of coronary events [6,7]. Thus, the 2013 American College of Cardiology/American Heart Association guidelines did not specify the LDL and non-HDL cholesterol goals needed to prevent atherosclerotic cardiovascular disease [8].

Dietary lifestyles may be related to the risk of cardiovascular disease; however, the mechanisms by which diet affects disease progression remain unknown. The effects of diet on the risk of cardiovascular disease have been extensively investigated in epidemiological and intervention studies [9,10]; epidemiological studies indicated that the habitual consumption of soy and fish contribute to reducing cardiovascular disease mortality rates to low as those among Japanese populations [9,11]. Among food components, soy protein and fish oil have been studied in detail in randomized clinical trials and animal studies in order to elucidate their relationships with a reduced risk of cardiovascular disease [9]. Soy protein increases the rates of cholesterol turnover, cholesterol oxidation, and fecal steroid excretion and reduces cholesterol absorption [12]. Omega-3 polyunsaturated fatty acids (PUFAs) in fish oil have been shown to exert hypolipidemic effects in the liver, inhibit platelet aggregation, and exert anti-inflammatory effects in vessels [13]. However, the effectiveness and precise underlying mechanisms of action of these food components remain unclear.

Inappropriate profiles of serum lipoproteins may cause atherosclerosis. Lipoproteins transport hydrophobic lipids through the bloodstream [14]. Lipoproteins have been divided into several classes [15]. Chylomicrons (CM) incorporate dietary lipids in the small intestine. Very-low-density lipoproteins (VLDL) incorporate endogenous lipids in the liver. These particles and their metabolized derivatives transport lipids to peripheral tissues. High-density lipoprotein (HDL) is responsible for the transport of lipids from peripheral tissues to the liver. We recently identified ubiquitous lipoprotein LDL-anti-protease complexes (LACs), which are 10-15-nm liver-derived particles that hold large amounts of cholesterol, in the serum of the rat [15]. Since LACs consist of cholesterol and anti-protease, they may promote atherosclerosis by supplying the material of the plaque and inhibiting plasmin activity. Despite this potential risk, since they have a very high density, LACs may have been wrongly categorized as HDL. Therefore, they may be important for elucidating the relationship between lipoprotein metabolomics and atherosclerosis.

Dietary soy protein and fish oil may improve lipoprotein profiles, thereby reducing the risk of vascular disease. A previous study reported that soy protein mainly reduced cholesterol and phospholipid levels in VLDL and LDL [16]. Furthermore, a fish oil-supplemented diet decreased plasma triacylglycerol (TG) levels by markedly lowering CM and VLDL levels [17]. Therefore, dietary soy protein and fish oil may favorably regulate lipoprotein metabolism and contribute to reducing the risk of atherosclerosis. In the present study, we investigated how soy protein-rich tofu and ω-3 PUFA-rich fish oil affect the types and amounts of serum lipoproteins in rats using a gel filtration system [15]. Additionally, a comprehensive analysis of the hepatic transcriptome was performed to examine changes in lipid metabolism, and the involvement of dietary factors in the modulation of circulating lipoproteins was demonstrated. Metabolomic and transcriptomic studies revealed that soy protein and fish oil independently modulated lipoprotein metabolism. These dietary components both markedly decreased the concentration of LAC1, which is presumed to be the cause of atherosclerosis.

## Materials and methods

### Materials

Powdered freeze-dried tofu [18], a source of soy protein, was purchased from Misuzu Corporation Co., Ltd. (Nagano, Japan). It contained 50.3 g/100 g protein and 34.4 g/100 g fat. Tuna-derived fish oil was purchased from NOF Corporation (Tokyo, Japan). Soybean oil and casein were purchased from Wako Pure Chemical Industries (Osaka, Japan). The crude protein content of casein was 88.7 g/100 g. The fatty acid compositions of soybean oil and fish oil were shown in S1 Table.

### Animals and diets

Male Sprague-Dawley rats were obtained from Charles River Japan (Kanagawa, Japan) at 4 weeks of age. Animals were housed individually in a room with a controlled temperature (23 ± 1°C) and humidity (55 ± 5%), and 12-h light-dark cycle for 5 days. Animals were randomly divided into 4 groups (*n* = 7–8) and assigned an experimental diet for 21 days: casein and soybean oil diet (CS); casein and fish oil diet (CF); tofu and soybean oil diet (TS); tofu and fish oil diet (TF). Casein and soybean oil were employed as a reference to tofu and fish oil for protein and fat sources, respectively. The total protein and fat contents of each diet were 200 and 150 g/kg, respectively. In fish oil diets, 50 g/kg of fish oil was replaced with the same amount of soybean oil (CF and TF). Since freeze-dried tofu contained large amounts of fat and protein, the amount of tofu that covered 100 g/kg of fat was approximately 290 g/kg (equivalent to 14.6% protein in diet). Thus, 60.9 g/kg of casein (equivalent to 5.4% protein) was supplemented in order to adjust dietary protein contents in the tofu diets (TS and TF). The compositions of the experimental diets were shown in S2 Table. Animals were allowed free access to food and water. Feces were collected for 3 days prior to dissection and stored at −30°C. At the end of the experimental period, animals were euthanized by collecting blood from the abdominal aorta under isoflurane anesthesia after being deprived of food for 3 h. Liver samples were immediately excised, weighed, frozen in liquid N_2_, and stored at −80°C. The present study was approved by the Review Board of the Animal Ethics of the Food Research Institute, NARO (approval number: H19-052) and followed institutional guidelines for the care and use of laboratory animals.

### Lipid analysis of the liver and feces

Hepatic lipids were extracted [19] and analyzed for TG [20] and cholesterol [21] as previously described. Feces were lyophilized and weighed. Total fecal bile acids were analyzed enzymatically with a modification as described elsewhere [22].

### Analysis of fatty acid compositions in the liver

TG in liver lipid extracts were separated by thin-layer chromatography, and fatty acid compositions were assessed by gas-liquid chromatography as described elsewhere [23].

### Analysis of lipoprotein profiles in serum

Serum lipoproteins were examined by the LipoSEARCH profiling service (Skylight Biotech Inc., Akita, Japan). Briefly, lipoproteins in serum were separated using a gel filtration HPLC system and the effluent was constitutively monitored for the concentrations of TG and cholesterol as described by Usui et al. [24]. The output patterns of HPLC for TG and cholesterol were fit to the curve using the least number of parameters [15]. This method estimated ten classes of lipoproteins and mimicked their elution patterns by a set of normal distributions. Since each class of lipoprotein had a similar size distribution among the diet classes, fitting started from values estimated in the previous study [15], and parameters were tuned to find a better fit to each data set. The appropriateness of the model was verified through the fit (S1 Fig).

### Analysis of mRNA profiles in the liver

In each of the groups, five rats with average food consumption, i.e., those with the highest and lowest values were eliminated, were used in microarray analyses. The extraction of hepatic RNA and sample preparation for the DNA microarray analysis were conducted according to a previous study [25]; after the normalization of raw signals, positive genes were selected and analyzed using a principal component analysis (PCA). Each sample was applied to one microarray chip (Rat Genome 230_2.0 GeneChip, Affymetrix, Santa Clara, CA). Microarray data were normalized using the SuperNORM data processing service (Skylight Biotech Inc.), and gene expression in each group was estimated [25,26]. To cancel interference by noise, positive genes were selected prior to PCA. The significance of dietary factors was statistically tested by a two-way analysis of variance (ANOVA) that was applied to the factors, Perfect Match (PM) cell sensitivity and dietary group effect [26]. The level of significance used for the selection was 0.01. The magnitude of the difference was estimated by taking trimmed means among differences calculated for PM data; in comparisons between CS and other diets, genes with Δz scores of more or less than 0.2 were selected. This magnitude corresponded to a 1.41-fold difference. A total of 4871 out of 31042 genes in the microarray satisfied the criteria for significance and differences (S3 Table). The selected genes and the groups of samples formed a matrix of multivariate data. The relationships between the genes and differences among the groups were further assessed using PCA [27]. The appearance frequency of key words, supplied for each gene by the manufacturer, was tested using a binominal distribution model. Among genes with absolute values that were larger than 0.1 in PC1 or 0.05 in PC2, words with *p*-values less than 0.0005 and appearing in more than four genes were selected. Microarray data have been deposited into the Gene Expression Omnibus (GEO) public repository, accession code GSE66371.

### Analyses of hepatic enzymatic activities involved in lipid metabolism

Enzymatic activities involved in fatty acid synthesis and oxidation in the liver were measured spectrophotometrically using the supernatant fraction of liver homogenates and total homogenates, respectively, as described previously [28].

### Statistical analysis

To estimate the significance of differences among the groups, a one-way ANOVA was used to avoid the issues associated with the multiplicity of tests. To clarify significant differences among the groups, the Tukey-Kramer test was used for post hoc analyses. Data are presented as means ± SD. The significance of differences was defined at a level of *p* < 0.05.

## Results

### Food consumption, body weight, and tissue weights

**Fig 1:**
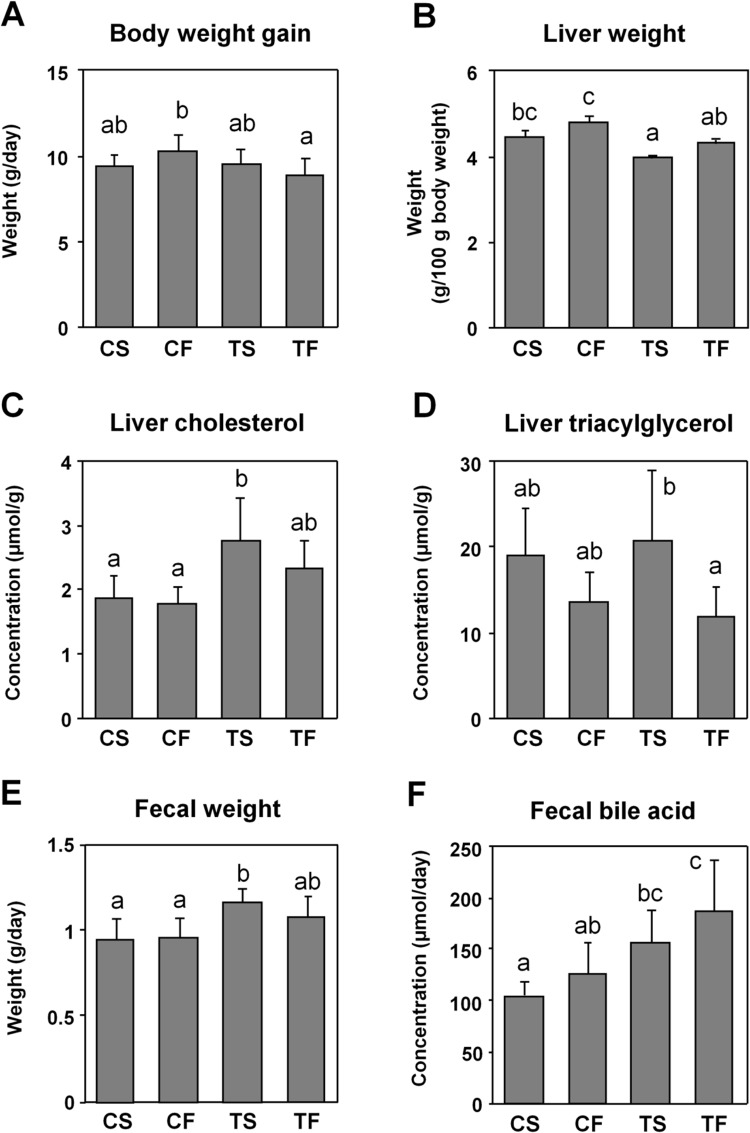
Growth and biochemical parameters of rats. (**A**) Body weight gain during the experimental period. (**B**) Liver weight in the experimental period. (**C**) Cholesterol and (**D**) TG levels in the liver. (**E**) Daily lyophilized fecal weight. (**F**) Fecal bile acid levels. C, Casein; T, tofu; S, soy oil; F, fish oil. Values are means ± SD, *n* = 7–8. Means without a common letter significantly differ, *p* < 0.05.

No significant differences were observed in food consumption and average energy intake among the groups throughout the experiment (S4 Table). No marked differences were observed in body weight gain (Fig 1A). Liver weight was lower in the TS group than in the CF group (Fig 1B). Hepatic cholesterol levels were increased by kori tofu (Fig 1C) and TG levels were significantly reduced by fish oil (Fig 1D). Similar to previous findings [22], tofu diets relatively, but not significantly increased daily fecal weights (Fig 1E), and fecal bile acid levels were significantly elevated by tofu diets and moderately increased by fish oil diets (Fig 1F). A previous study reported that the high-molecular-weight fraction (HMF), a fraction increased by the coagulation and freeze-drying of tofu protein, may increase fecal weight and has a high bile acid-binding capacity [22].

### Hepatic fatty acid composition

**Fig 2:**
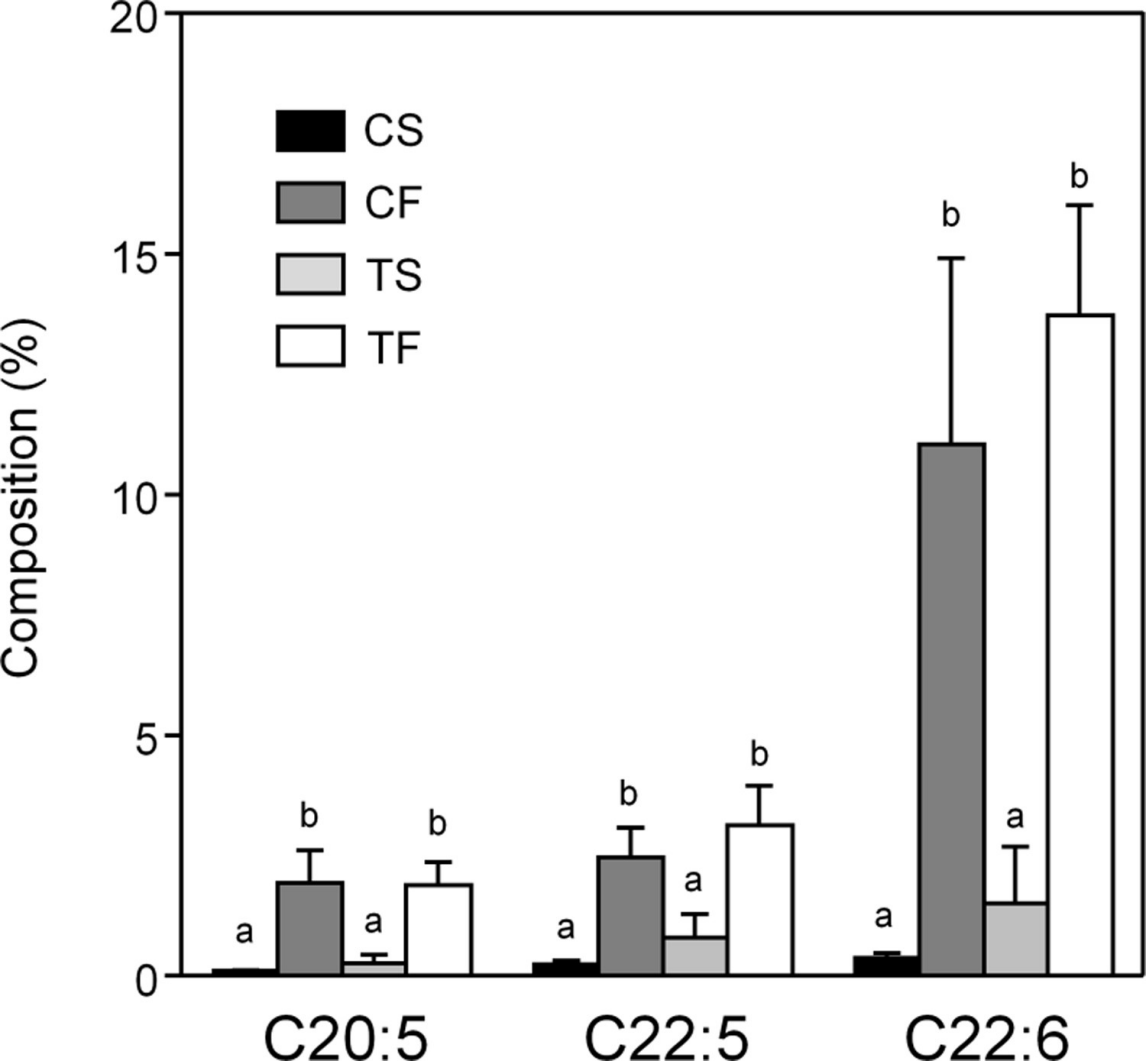
Ratio of selected *ω*-3 fatty acids to total fatty acids in liver TG. C, Casein; T, tofu; S, soy oil; F, fish oil. Values are means ± SD, *n* = 7–8. Means without a common letter significantly differ, *p* < 0.05.

Hepatic fatty acid compositions in TG were markedly affected by dietary lipids (S5 Table). Fish oil feeding increased the ratio of *ω*-3 fatty acids to total fatty acids, mainly as EPA (eicosapentaenoic acid; C20:5), DPA (docosapentaenoic acid; C22:5), and DHA (docosahexaenoic acid; C22:6) (Fig 2).

### Total cholesterol and TG levels in serum

**Fig 3:**
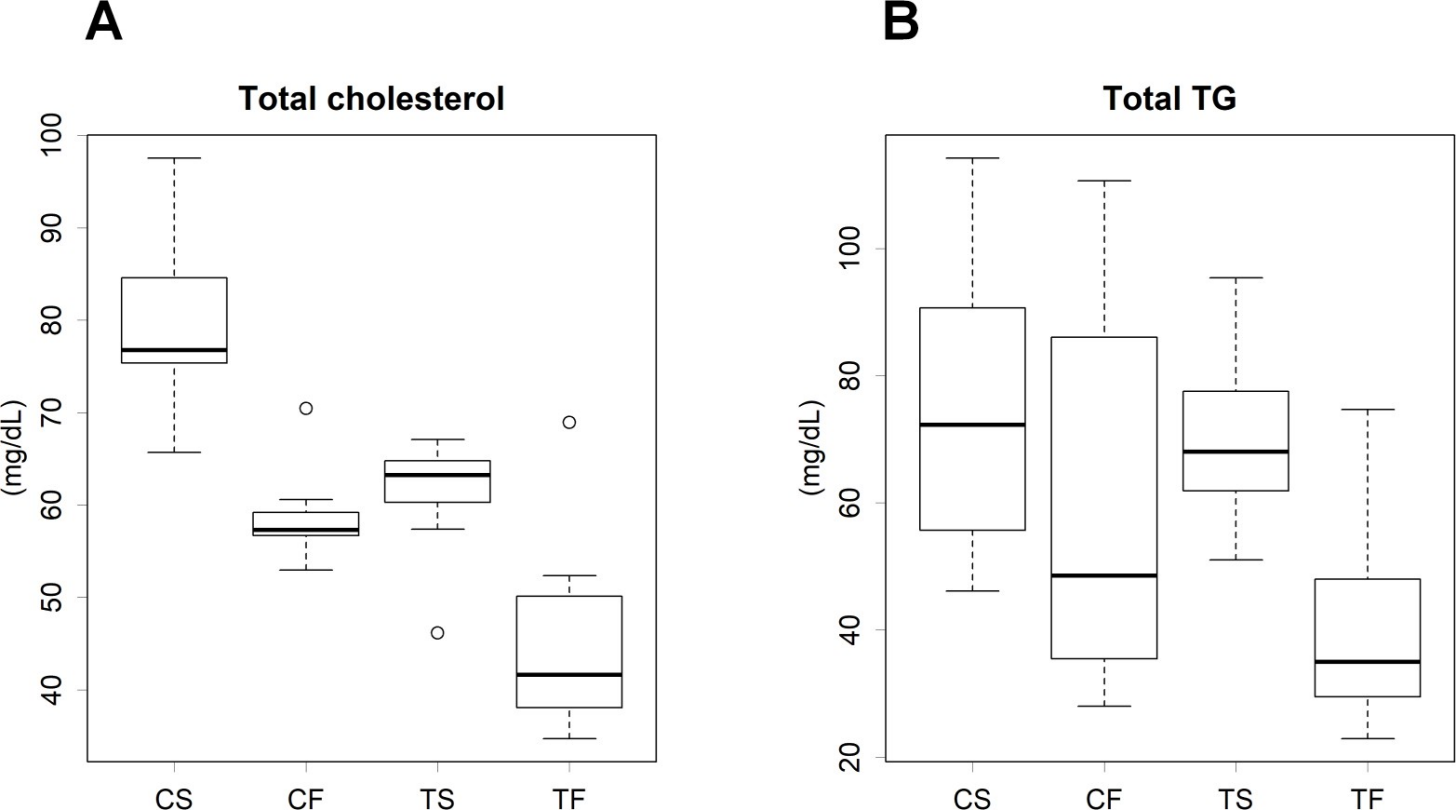
Serum lipid levels in rats. (**A**) Cholesterol. (**B**) TG. C, Casein; T, tofu; S, soy oil; F, fish oil. Values are means ± SD, *n* = 7–8. Results of statistical analyses are presented in S1 Appendix. Results of the post hoc analysis and 95% family-wise confidence levels are also shown. Results of statistical analyses are presented in S1 Appendix.

Tofu and fish oil significantly decreased serum total cholesterol levels more than casein and soy oil (Fig 3A). TG levels were lower in rats fed tofu and fish oil than in those fed casein and soy oil (Fig 3B).

### Particle sizes of lipoproteins

**Fig 4:**
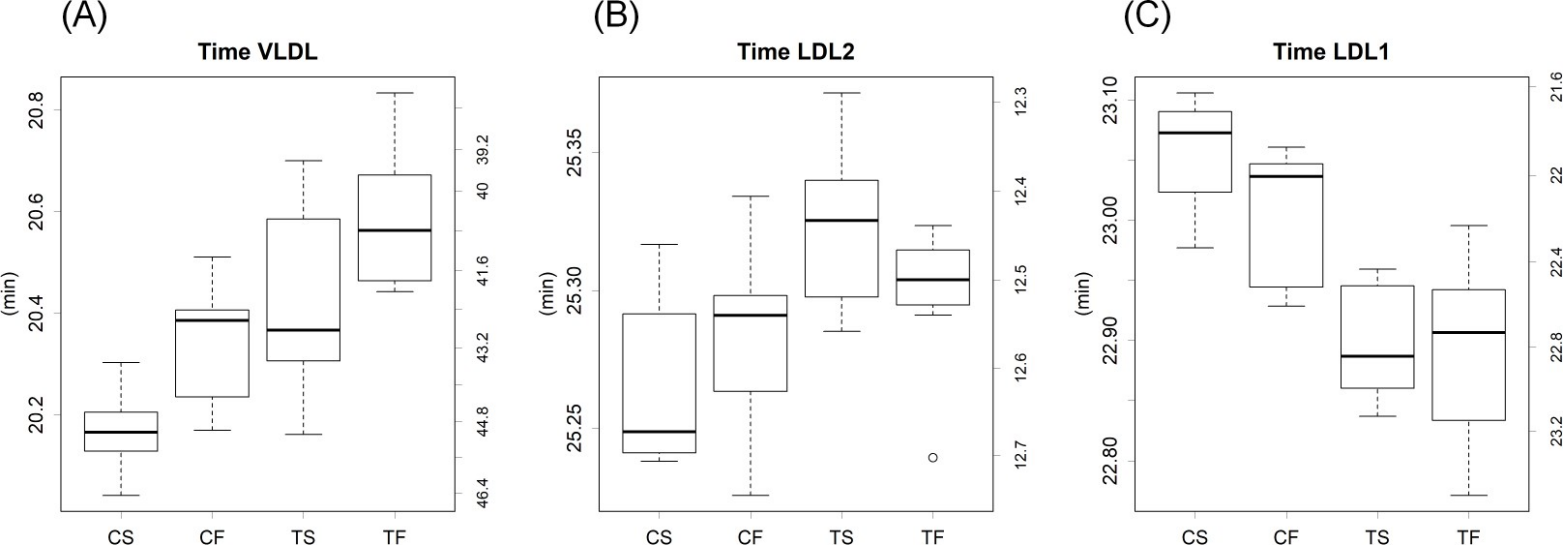
Elution times of lipoprotein classes. (**A**) VLDL, (**B**) LDL2, and (**C**) LDL1. C, casein; T, tofu; S, soy oil; F, fish oil. The bottom and top of the box are the first and third quartiles, respectively, and the band inside the box indicates the second quartile. Small circles show outliers. Results of statistical analyses are presented in S1 Appendix.

**Fig 5:**
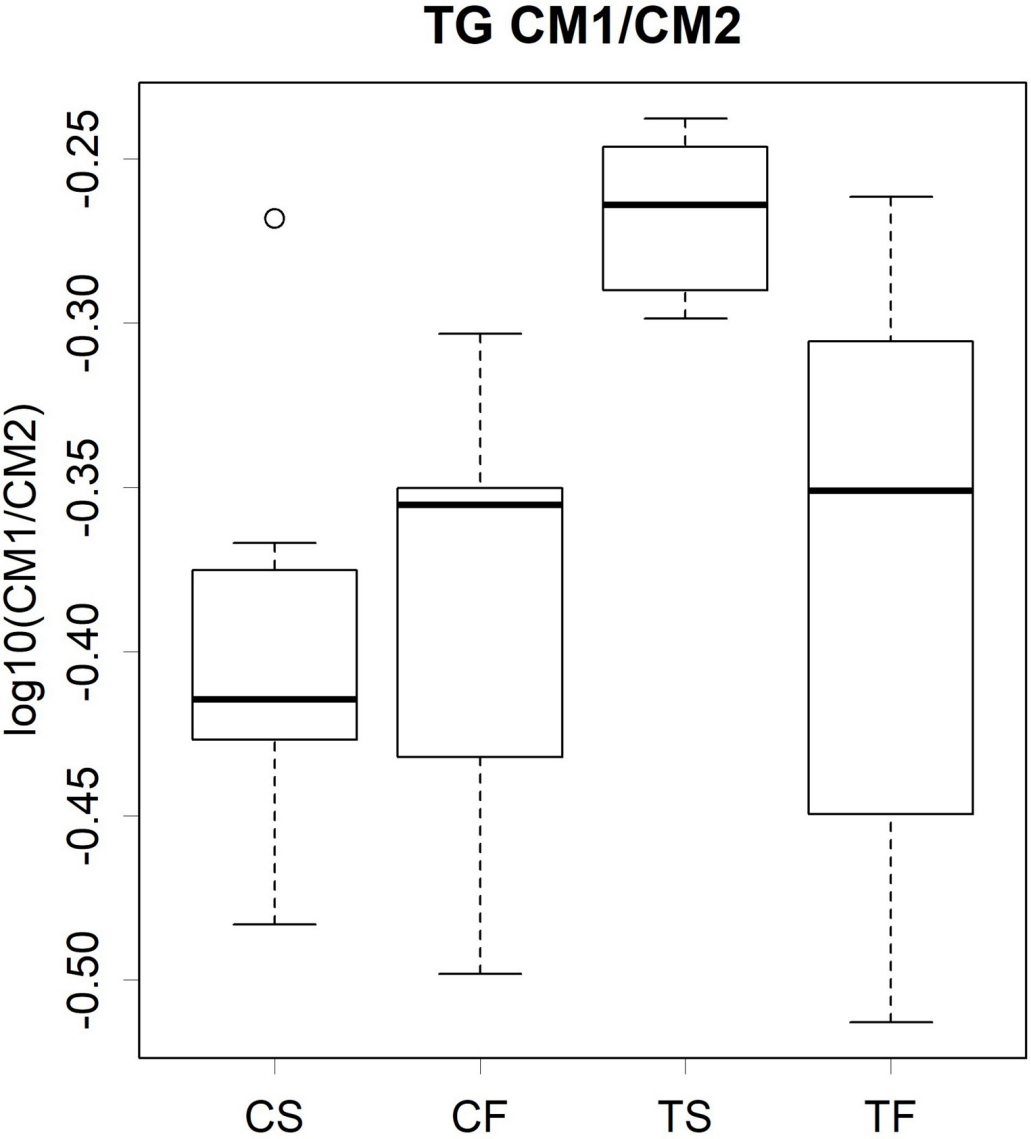
Ratio of CM1 to CM2 in serum TG. C, Casein; T, tofu; S, soy oil; F, fish oil. The bottom and top of the box are the first and third quartiles, respectively, and the band inside the box indicates the second quartile. Small circles show outliers. Results of statistical analyses are presented in S1 Appendix.

Tofu modulated the particle sizes of several lipoprotein classes (S2 Fig). The peak time for the normal distribution is known to be proportional to the logarithm of the particle size [15]; thus, it represents the diameter of a lipoprotein. The elution times of VLDL and its metabolite LDL2 were delayed by the tofu diets, whereas those of LDL1 were earlier (Fig 4). Regarding CM, the ratio of the earlier peak CM1 (larger than the pore size of our column) [15] to the later peak CM2 was higher in the TS group than in the other groups (Fig 5). However, these variations in particle sizes were within a several percent range of their size. They did not correlate with the total amount of lipids in lipoproteins or variations in particle volumes. Therefore, the modulation of particle sizes may represent a qualitative change in apolipoproteins as described later.

### Amounts of cholesterol and TG in lipoprotein classes

**Fig 6:**
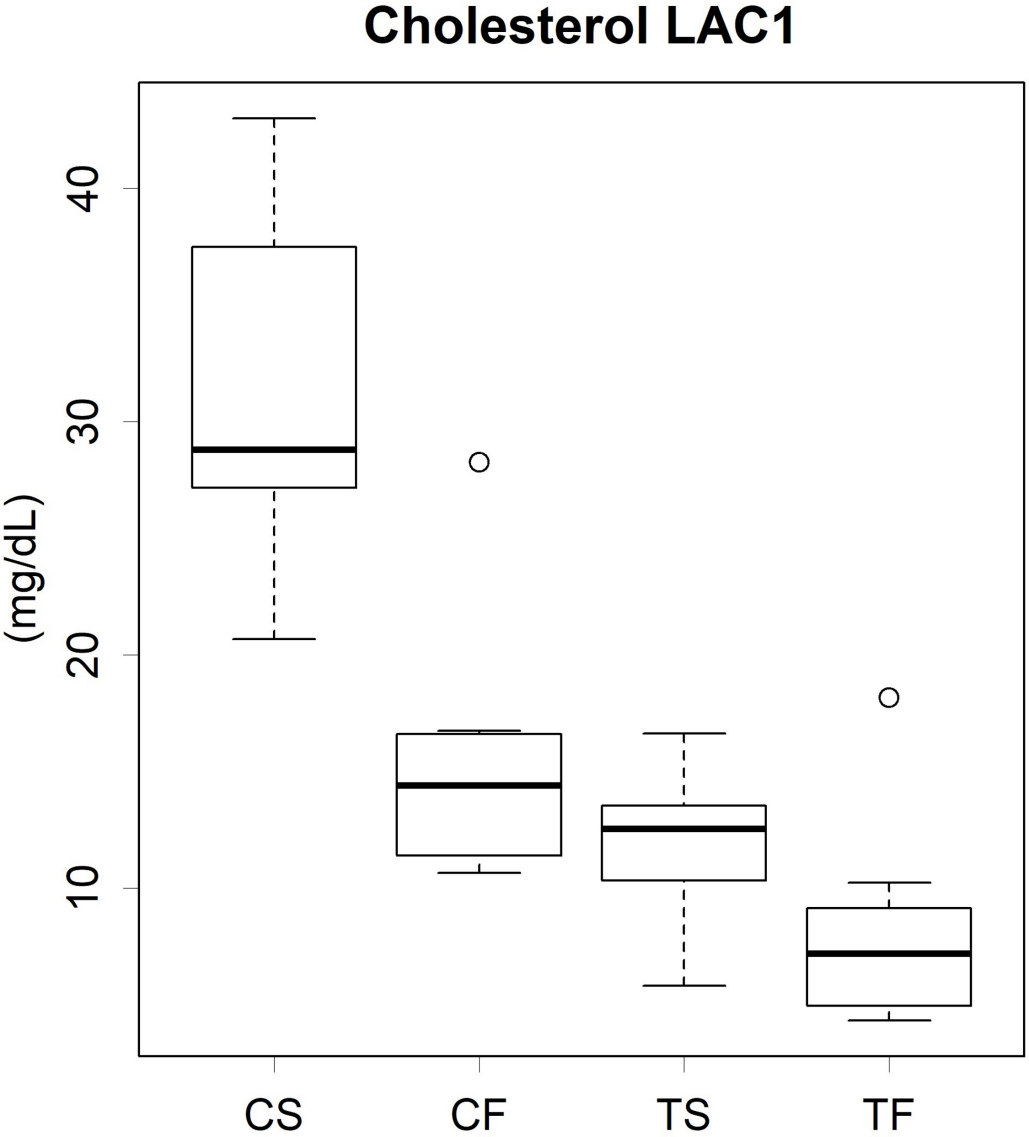
Cholesterol level of the lipoprotein class LAC1. C, Casein; T, tofu; S, soy oil; F, fish oil. The bottom and top of the box are the first and third quartiles, respectively, and the band inside the box indicates the second quartile. Small circles show outliers. Results of statistical analyses are presented in S1 Appendix.

Significant differences in cholesterol levels among the groups were observed in the lipoprotein classes (S3 Fig). Fish oil reduced the cholesterol levels of several lipoprotein classes (CM1, CM2, VLDL, LDL1, LDL2, LAC1, and HDL1). Cholesterol-rich lipoprotein LDL2 and LAC1 were sensitive to the fish oil diets; significant differences were observed between CS-TF and TS-TF in LDL2, and CS-CF and CS-TF in LAC1. The value for LAC1 was almost 50% lower in the fish oil groups than in the soy oil groups (Fig 6). The TS diet did not reduce the cholesterol levels of CMs, VLDL, LDLs, or LAC2 from those with the CS diet. In contrast, the level of LAC1, a major cholesterol-rich lipoprotein class, in the TS group was approximately 1/3 that in the CS group.

Although significant differences were not always observed in the level of TG, fish oil and tofu reduced these values in some lipoprotein classes (S4 Fig). Fish oil slightly reduced TG levels in CMs. Similar to cholesterol levels, TG levels were suppressed by fish oil and tofu in LAC1. Moreover, these dietary effects were clearly observed in the ratio of TG to cholesterol in lipoprotein classes. Tofu increased the ratio in CMs, whereas it reduced it in its metabolite LDL1 (S5 Fig).

### Transcriptome profile in the liver based on PCA

**Fig 7:**
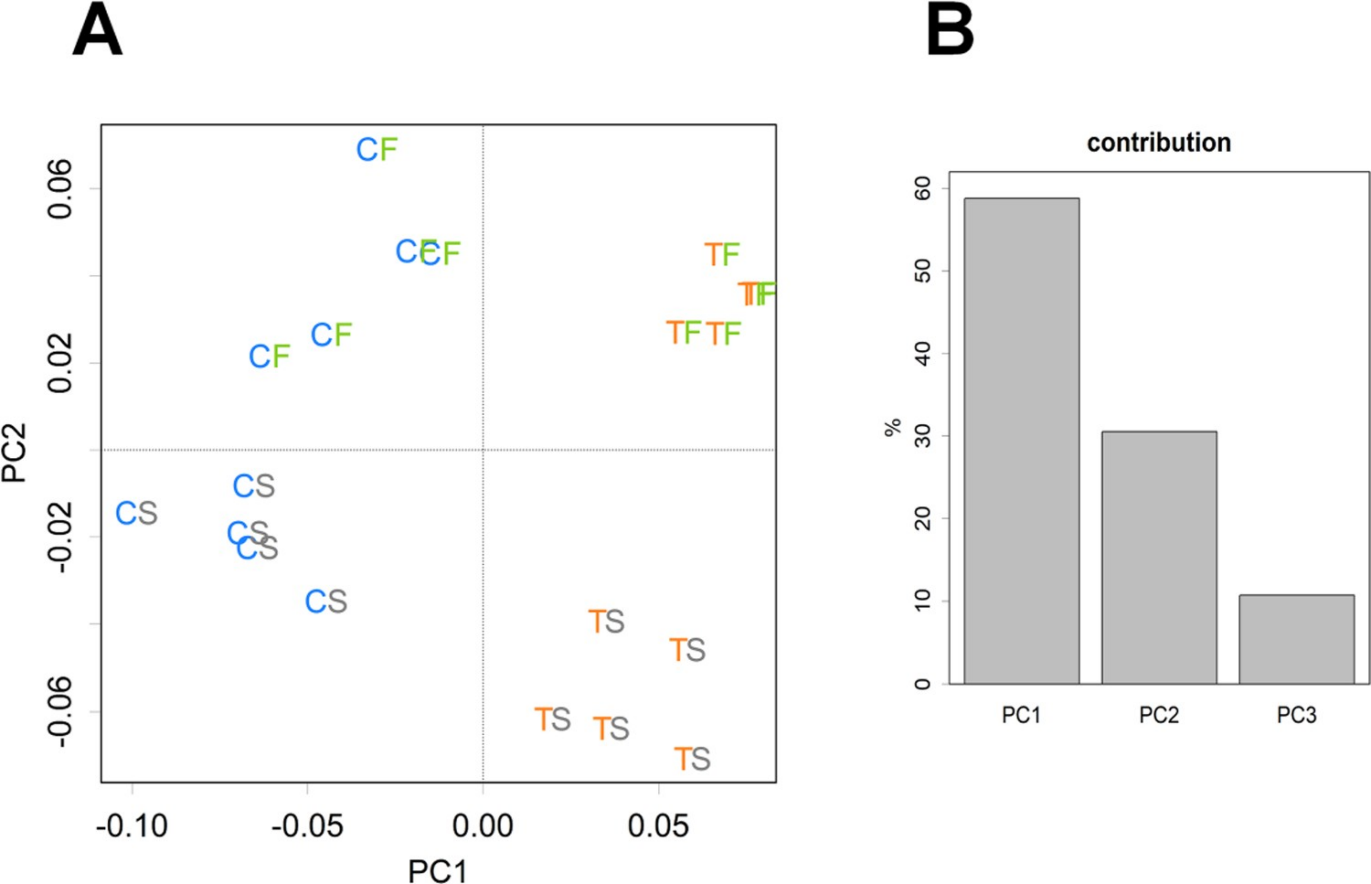
Principal component analysis applied to individual samples. (**A**) PC for samples. Score plots of PC1 compared with PC2 were derived from hepatic gene expression in rats: each plot represents a rat sample. C, Casein; T, tofu; S, soy oil; F, fish oil. (**B**) Contribution of PC axes (%). PC for genes are presented in S6 and S7 Tables.

The matrix of transcriptome data of samples by genes was analyzed using PCA (Method). The multivariate analysis separated the experimental groups into two individual directions. As shown in Fig 7A, PC1 and PC2 represented differences in dietary protein and fat sources, respectively; each group was clearly divided by the grids. These results suggested that protein and fat sources independently affect hepatic gene expression, and that gene expression reflected independent responses to tofu or fish oil (the contribution of PCs was shown in Fig 7B). The numbers of genes selected for PC1 and PC2 were 259 and 546, respectively, and only 96 were found in both. Scores for the selected genes in PC1 and PC2 showed Pearson’s correlation of -0.0641, negating a correlation between the axes.

In PC1, tofu suppressed the mRNA expression of genes related to fatty acid synthesis, fatty acid elongation, fatty acid desaturation, cholesterol synthesis, and fatty acid transport; this was clear from the annotated functions and keywords of the genes provided by the protein source (Tables 1 and S6). Tofu depressed the PC1-negative gene group including the lipogenic rate-limiting enzyme. Moreover, other lipogenic genes, such as fatty acid synthases, ATP-citrate lyase, fatty acid desaturases, and SREBP-1, were involved in the gene list (S3 Table).

**Table 1:** Over-represented gene ontology involving hepatic genes expressed differently among groups (PC1).

Category of gene ontology biological process	genes	P-value
oxidation reduction	36	0.0.E+00
metabolic process	36	1.3.E-14
transport	24	2.0.E-03
lipid metabolic process	11	2.2.E-07
response to drug	11	7.8.E-06
regulation of transcription	10	9.7.E-04
lipid biosynthetic process	9	6.8.E-09
response to nutrient	9	4.5.E-07
fatty acid biosynthetic process	8	4.5.E-10
response to organic substance	7	1.1.E-05
response to organic cyclic substance	7	2.6.E-05
glucose metabolic process	6	1.8.E-06
response to glucocorticoid stimulus	6	3.2.E-05
response to toxin	5	4.3.E-06
steroid biosynthetic process	5	1.8.E-05
aging	5	2.1.E-04
xenobiotic metabolic process	4	4.1.E-07
liver development	4	2.4.E-04
regulation of cell cycle	4	3.0.E-04
fatty acid metabolic process	4	9.0.E-04
positive regulation of cell migration	4	9.0.E-04

Yellow cells show the categories related to lipid metabolism.

On the other hand, PC2-positive genes suggested that the ingestion of fish oil stimulated the mRNA expression of genes involved in lipid metabolism and fatty acid β-oxidation (Tables 2 and S7). The fatty acid β-oxidation pathway contains several enzymes that catalyze irreversible reactions; the mRNA expression of these enzymes was stimulated well in the fish oil-fed groups. For example, the expression of acyl-CoA dehydrogenases and 3-hydroxybutyrate dehydrogenase, in addition to irreversible thioesterases, was induced. Moreover, the enzymes of the genes required to catabolize the unsaturated fatty acids, enoly-CoA hydratase and enoyl-CoA Δ-isomerase, were present in the list. Other enzymes facilitating fatty acid β-oxidation, such as carnitine acyltransferases and acyl-CoA oxidase, and fatty acid catabolism represented by acyl-CoA thioesterases were also involved in the PC2-positive group. These reactions may induce the production or degradation of acetyl-CoA and/or fatty acid re-synthesis. Since the levels of mRNAs for some subunits of the rate-limiting enzyme of TCA cycle isocitrate dehydrogenase were slightly induced by fish oil, these enzymes may be involved in the oxidation of acetyl-CoA (S7 Table). The mRNA expression of malonyl-CoA decarboxylase, which plays a role in the reverse reaction of acetyl-CoA carboxylase, was strongly stimulated by fish oil. Although the transcript level of acetyl-CoA carboxylase was increased in the casein groups, elevated levels of malonyl-CoA decarboxylase may cancel its activity and reduce the total production of fatty acids in the fish oil groups.

**Table 2:** Over-represented gene ontology involving hepatic genes expressed differently among groups (PC2).

PC2	genes	P-value
metabolic process	49	2.4.E-14
lipid metabolic process	32	0.0.E+00
oxidation reduction	29	7.1.E-12
fatty acid metabolic process	23	2.1.E-14
responses to drugs	13	1.1.E-04
phosphate transport	10	7.2.E-06
fatty acid beta-oxidation	9	3.2.E-12
responses to organic cyclic substances	9	4.4.E-05
long-chain fatty acid metabolic process	8	6.4.E-13
responses to nutrients	8	2.8.E-04
acyl-CoA metabolic process	7	4.0.E-10
responses to a glucocorticoid stimulus	7	1.7.E-04
heart development	7	6.0.E-03
very-long-chain fatty acid metabolic process	6	1.1.E-10
positive regulation of apoptosis	6	6.9.E-03
regulation of fatty acid oxidation	5	2.4.E-07
regulation of the cell cycle	5	5.4.E-04
cell migration	5	1.8.E-03
lipid biosynthetic process	5	2.2.E-03
locomotory behavior	5	2.5.E-03
responses to an estrogen stimulus	5	5.1.E-03
responses to organic substances	5	9.8.E-03

Yellow cells show the categories related to lipid metabolism.

### Enzyme activities involved in lipid metabolism in the liver

Alterations in hepatic lipid metabolism at the level of the transcriptome were verified by some enzymatic activities. The enzyme activities involved in fatty acid oxidation were significantly suppressed by tofu and fish oil (S8 Table). The fish oil diets largely induced the activities of fatty acid β-oxidation enzymes, while the tofu diets slightly reduced peroxisomal fatty acid oxidation.

## Discussion

Changes in the particle sizes of LDL by tofu may reflect differences in apoproteins, which influences the qualitative properties of lipoproteins. Differences in particle sizes (represented in time; S2 Fig) were less prominent than those in the quantity of lipoproteins (represented in the levels of cholesterol and TG; S3 and S4 Figs) among individual samples. Furthermore, the log normal distribution and its σ were nearly constant in the dietary group (S1 Fig), suggesting that the particle sizes of lipoproteins were generally conserved and stable [15]. In terms of a process of elimination, differences in particle sizes appear to represent those in the types of components, possibly apolipoproteins, instead of the amount of lipids being transported. Since apolipoproteins are specific to their receptors, the characteristics of lipoproteins vary depending on the proteins contained within. Apolipoprotein B (ApoB), the primary apolipoprotein of LDL particles, is a candidate for the cause of these changes. The protein size of ApoB is known to be modulated by dietary conditions via RNA editing [29]. In principle, the larger molecular weight form (ApoB100) is known to be synthesized in the liver, whereas the smaller molecular weight form (ApoB48) is synthesized in the intestine. However, evidence for this tissue specificity is limited. ApoB100 was predominant in the fetal human intestine, but disappeared in the adult intestine in which ApoB48 was synthesized [30]. Thus, the tissue-specific localization of apolipoproteins is possibly altered by diets. Alternatively, lipoproteins may include unknown apolipoproteins. We previously reported that lipoproteins may contain more protein than previously considered [15]. Unfortunately, we were unable to identify and directly measure these apolipoproteins in the present study. In principle, proteins are more difficult to identify than RNA because their biochemical properties are divergent.

We speculate that differences in the particle sizes of lipoproteins may affect their turnover rates. Tofu slightly increased LDL1 particle sizes, but decreased those of VLDL and LDL2 (Fig 4). Furthermore, size differences were observed in CM as an increased CM1/CM2 level (Fig 5), which implies that particles at the void volume of the gel-filtration column increased. As described above, size differences may be reflected by the types of apolipoproteins. Therefore, the affinity of lipoproteins and their receptors vary depending on the constituents of apolipoproteins. Increases in the levels of LDL1 and VLDL classes in the TS group may be influenced by these changes (S3 Fig).

Since the level of LAC1 was markedly higher than that of LAC2, the total level of LAC was considered to be strongly suppressed by tofu and fish oil (S3 Fig). LAC1 and LAC2 are a complex of LDLs and anti-proteases [15]. The anti-proteases alpha-1-macroglobulin (A1m) and alpha-1-inhibitor 3 (A1i3) were detected in LAC1 and LAC2, respectively. In the CS group, LAC1, along with LDL1 and LDL2, contained a large amount of serum cholesterol, whereas the level of LAC2 was approximately 1/10 that of LAC1 (S3 Fig). Tofu and fish oil individually, but not additively halved the level of LAC1 (S3 Fig). Although limited information is currently available on these proteases, if LAC, as a complex of thrombotic material and an anti-fibrinolysis enzyme, is a cause of atheroma, soy protein and fish oil have the potential to prevent the progression of atherosclerosis. We speculate that fish oil decreased total cholesterol levels in serum, while tofu suppressed the synthesis of cholesterol. The mRNA levels of genes involved in hepatic cholesterol synthesis were lower in the tofu diet groups than in the casein groups (S2 Table). Since soy protein is known to increase the rates of cholesterol turnover, cholesterol oxidation, and fecal steroid excretion and reduce cholesterol absorption [12], these actions may have contributed to decreasing serum cholesterol levels in the tofu diet groups. In contrast, fish oil effectively suppressed cholesterol levels in the major cholesterol-rich lipoproteins, particularly LDL1 and LAC1 (S3 Fig). Moreover, the ingestion of fish oil stimulated the mRNA expression of genes involved in lipid metabolism and fatty acid β-oxidation (Tables 2 and S7). Fatty acids are esterified with cholesterol stored in lipoproteins [31]. Thus, we speculated that esterified fatty acids were rapidly degraded, and the cholesterol residue was excreted as a bile acid into feces. However, the precise mechanisms underlying this regulation were not elucidated in the present study.

Since tofu and fish oil independently affected the levels of different lipoprotein classes in serum (S3 Fig) and hepatic transcriptomes (Fig 7 and S6 and S7 Tables), the mechanisms underlying this regulation by these dietary factors differ. Tofu suppressed mRNA expression by regulating hepatic fatty acid synthesis (S6 Table). These results were consistent with our previous findings [25] and other studies [32]. Fish oil facilitated the expression of genes involved in fatty acid oxidation and catabolism (S7 Table), which is in agreement with previous findings [33].

The suppression of the synthesis of hepatic fatty acids and cholesterol by tofu (Tables 1 and S6) suggested that circulating levels of TG and LDL were sufficient to satisfy physiological demands in rats fed the tofu diets. The levels of CM2 and VLDL (major carriers of TG) and LDL1 and LDL2 (major carriers of cholesterol) in tofu groups were not markedly lower or higher than those in the CS group (S3 and S4 Figs). These results implied that abundant TG was supplied by the diet. Moreover, the ratio of TG to cholesterol in CM1 and CM2 was higher in the tofu groups (S5 Fig): this increase suggested that the absorption efficacies of TG and cholesterol varied for some reason even though food consumption did not significantly differ among the groups (S4 Table). In contrast to CM, the ratio of TG to cholesterol was similar in VLDL classes, and was significantly lower in the LDL1 class when tofu-fed animals were compared with casein-fed animals (S5 Fig). Tofu may have increased the chance of contact between TG carriers and lipase in peripheral tissue or the activity of lipoprotein lipase. Since this was not observed in LDL2, the latter may be excluded as a possibility. The increased affinity of TG-rich lipoproteins for lipoprotein lipase was also proposed. Increased TG absorption in the intestines and its transport from lipoproteins to peripheral tissues may contribute to sufficient deposits of TG on adipose tissues. Furthermore, the levels of LDL1 and LDL2 in the tofu groups were higher and slightly lower, respectively, than those in the casein groups (S3 Fig), indicating that serum cholesterol and TG were sufficient to supply lipids to peripheral tissues. Therefore, the suppression of lipogenesis may have contributed to a decreased burden on the liver, which, in turn, slightly reduced liver weights.

Our results suggested that the intake of fish oil caused the excessive degradation of PUFAs in the liver. Some PUFAs, such as EPA and DHA, are essential for mammals because of the lack of several types of desaturases [34]; fish oil is a good source of these fatty acids. The fatty acid composition of the liver was strongly influenced by dietary fat (Fig 3 and S5 Table). Excessive PUFAs alter cell membrane fluidity [35], and ω-3 fatty acid epoxides mediate signal transduction that enhances mast cell activation and anaphylaxis; this may be cytotoxic for cells [36]. Inflammatory cells and molecules are recruited and accumulate on atherosclerotic lesions, and these lesions are enlarged during the process of dead cell clearance [37]. Thus, the excessive accumulation of PUFAs may contribute to the progression of inflammation and atherosclerosis. The saturation and desaturation of fatty acids are reversible reactions. The cytotoxicity of excessive PUFAs may be attenuated if mammals possess appropriate enzymes: i.e., desaturases; although these enzymes were named from a single direction, the reactions are reversible; ω-3 fatty acids are essential because animals do not have desaturases. Therefore, excess fatty acids need to be stepwise oxidized to acetyl-CoA. We speculated that the induction of hepatic fatty acid oxidation in rats fed fish oil indicated the degradation of toxic PUFAs. The acetyl-CoA produced may be used to resynthesize fatty acids; however, the levels of transcriptome (S7 Table) and enzymatic activity (S8 Table) did not indicate that acetyl-CoA was used in fatty acid synthesis. Fish oil increased the mRNA level of malonyl-CoA decarboxylase, catalyzing the reverse reaction of a key enzyme in fatty acid synthesis (S7 Table). Fish oil reduced almost all classes of lipoproteins (S3 Fig). Fish oil may suppress hepatic fatty acid synthesis, and acetyl-CoA was oxidized or used for other reactions. Since the selective β-oxidation of unsaturated fatty acids does not appear to be possible, *de novo* synthesized fatty acids may be at least partly degraded when fatty acid β-oxidation is stimulated. In this case, energy is dissipated for the elongation of fatty acid chains. The suppression of fatty acid synthesis may prevent wasted energy expenditure. Moreover, liver weights in the fish oil groups may have increased due to the detoxification of PUFAs.

CM may be preferentially retrieved by the liver. Some sterols, such as phytosterols and coprostanol, cannot be metabolized and may be toxic in animals. They are included in diets; while most are removed [38], some parts may remain in CM. Animals have a limited ability to degrade cholesterol and its derivatives, and, thus, they have to be secreted into the intestines [39]. The liver has multiple options for retrieved TG and cholesterol: it may degrade TG by hepatic lipase, consume cholesterol to produce bile acid, and distribute both by secreting VLDL and LACs [40].

**Fig 8:**
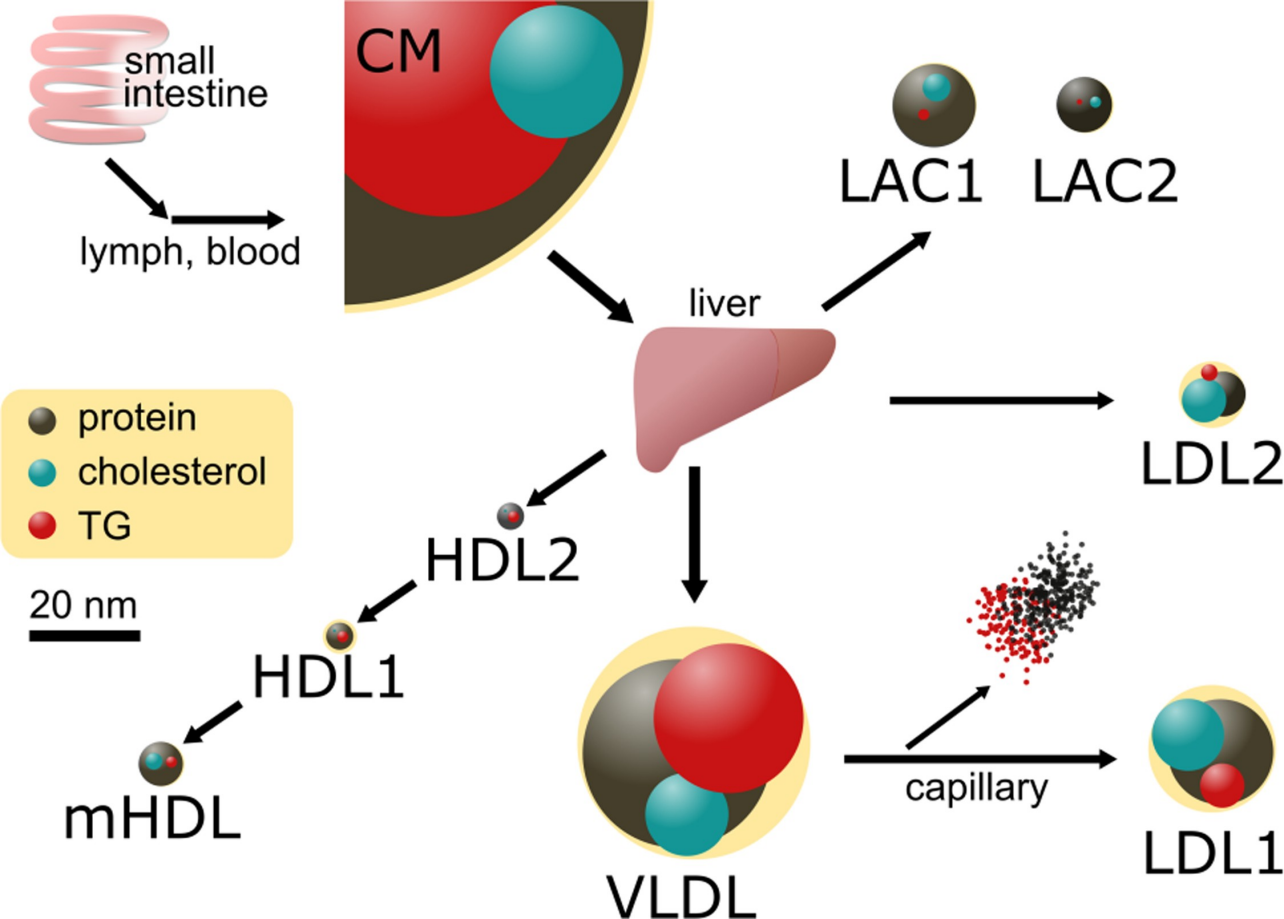
Alternative schematic flowchart of lipoprotein metabolism in rat. Relative amounts of cholesterol, TG, and proteins are indicated as spheres on a pale-yellow background that indicates the size of the lipoprotein.

We herein propose an alternative pathway model for lipoprotein metabolism considering the detoxification of PUFAs and steroids (Fig 8). In this model, CM is preferentially captured by the liver, degraded, and reconstructed into smaller lipoproteins. TG is transported to peripheral tissues by VLDL. However, the present model is not opposed to our previous model [15]. It is another aspect that is observed from different directions of the same phenomenon. The probability of one CM particle being metabolized in any tissue may be influenced by the affinity between the apolipoprotein included in CM and lipases in peripheral tissues. Since CM is not necessarily a homogeneous particle, affinity may vary depending on the apolipoprotein in CM.

Although the lipoprotein profile in serum was significantly changed by dietary protein and fat sources, it is noteworthy that body weight gain was similar among the groups (Fig 1A). Neither tofu, which exclusively suppressed LAC levels, nor fish oil, which modulated various classes of lipoprotein levels, affected the growth of animals. These dietary components efficiently halved LAC levels. These results suggest that serum lipid profiles were favorably improved without affecting healthy growth.

We assume that the levels of lipoproteins transporting TG and cholesterol to peripheral tissues are generally maintained at specific levels (Figs 3 and S3 and S4) through a similar mechanism as glucose homeostasis. However, this regulation appears to be exceptional in the TF group. Total cholesterol levels were significantly higher in the CS group than in the other groups (Fig 3A). This was mostly attributed to a marked increase in the level of LAC1 (Fig 6), while cholesterol levels were not increased in other lipoprotein classes transporting cholesterol, such as VLDL, LDL1, and LDL2 (S3 Fig). The lipoprotein profile may be assessed by the metabolic balance between the synthesis and degradation of substances. The transcriptome analysis revealed that fatty acid synthesis was significantly suppressed in the tofu groups (Tables 1 and S6), whereas this alteration was not observed in the metabolome of lipoproteins. Therefore, homeostasis may be maintained at the level of the metabolome. Alternatively, the demand for cholesterol in peripheral tissue may have been reduced in the tofu groups. In the present study, the health of rats did not appear to be impaired, possibly because the experimental period was not long enough to perturb physiological homeostasis.

According to serum lipoprotein profiles, the CS diet appears to be the most plausible combination of dietary components to induce atherosclerosis among the groups. Although sufficient cholesterol circulated in the bloodstream, why did the CS diet induce the synthesis of cholesterol in the liver? Furthermore, why was the level of LAC1 significantly higher in the CS group than in the other groups (S3 Fig)? The ratio of TG to cholesterol in the VLDL class was not affected by the diet (S4 Fig). A certain number of cholesterol molecules may be required to assemble LAC1, regardless of the demand for cholesterol in peripheral tissue. Therefore, cholesterol may be necessary as a carrier in order to maintain a constant level of TG. Since mammals are not able to fully hydrolyze cholesterol, excess cholesterol must be removed mainly through its conversion to bile acids [41]. A sufficient lipid level was maintained in the peripheral tissue of rats fed the CS diet and the voluntary uptake of lipids from the diet was not essential; therefore, the concentration of fecal bile acid was presumably not increased. Mammals have adapted to starvation throughout almost the whole process of evolution, and, thus, they may not have gained a sufficient ability to reduce excess cholesterol. Excess LDL1 and LDL2 are presumed to be recovered by the liver. However, re-incorporating cholesterol into VLDL may be difficult because the recovery and synthesis of VLDL occur in different hepatocytes; if these processes were performed within a cell, the cell has to distinguish the particles of likely protein components and sizes: particles under development and recovery. Therefore, reloading may require the intrahepatic transfer of recovered cholesterol, which requires energy and time [42]. LAC1, which contains a large amount of serum cholesterol, appears to be utilized as a convenient cholesterol pool; however, this pool may be a risk factor for cardiovascular disease.

## Supporting information

S1 Fig Curve fitting of the lipoprotein profile of each animal. (ZIP)

S2 Fig Peak time for the normal distribution of lipoprotein classes. (ZIP)

S3 Fig Cholesterol levels in lipoprotein classes. (ZIP)

S4 Fig TG levels in lipoprotein classes. (ZIP)

S5 Fig The ratio of TG to cholesterol in lipoprotein classes. (ZIP)

S1 Table Fatty acid compositions of dietary fat sources. (DOCX)

S2 Table Compositions of experimental diets. (DOCX)

S3 Table A full list of differentially expressed probe sets in the liver of rats. (XLSX)

S4 Table Growth parameters of rats. (DOCX)

S5 Table Fatty acid compositions of hepatic TG in rats. (DOCX)

S6 Table A list of differentially expressed probe sets involved in the PC1 cluster. (XLSX)

S7 Table A list of differentially expressed probe sets involved in the PC2 cluster. (XLSX)

S8 Table Activities of enzymes involved in hepatic fatty acid synthesis and oxidation. (DOCX)

S1 Appendix Statistical tests of Figs 3, 4, 5, and 6. (ZIP)
