# Supplementary figures and images for: Tofu and fish oil independently modulate serum lipid profiles in rats: Analyses of 10 class lipoprotein profiles and the global hepatic transcriptome
Source: PLoS One. 2019 Jan 17;14(1):e0210950. doi: 10.1371/journal.pone.0210950 (PMC6336308; doi:10.1371/journal.pone.0210950)

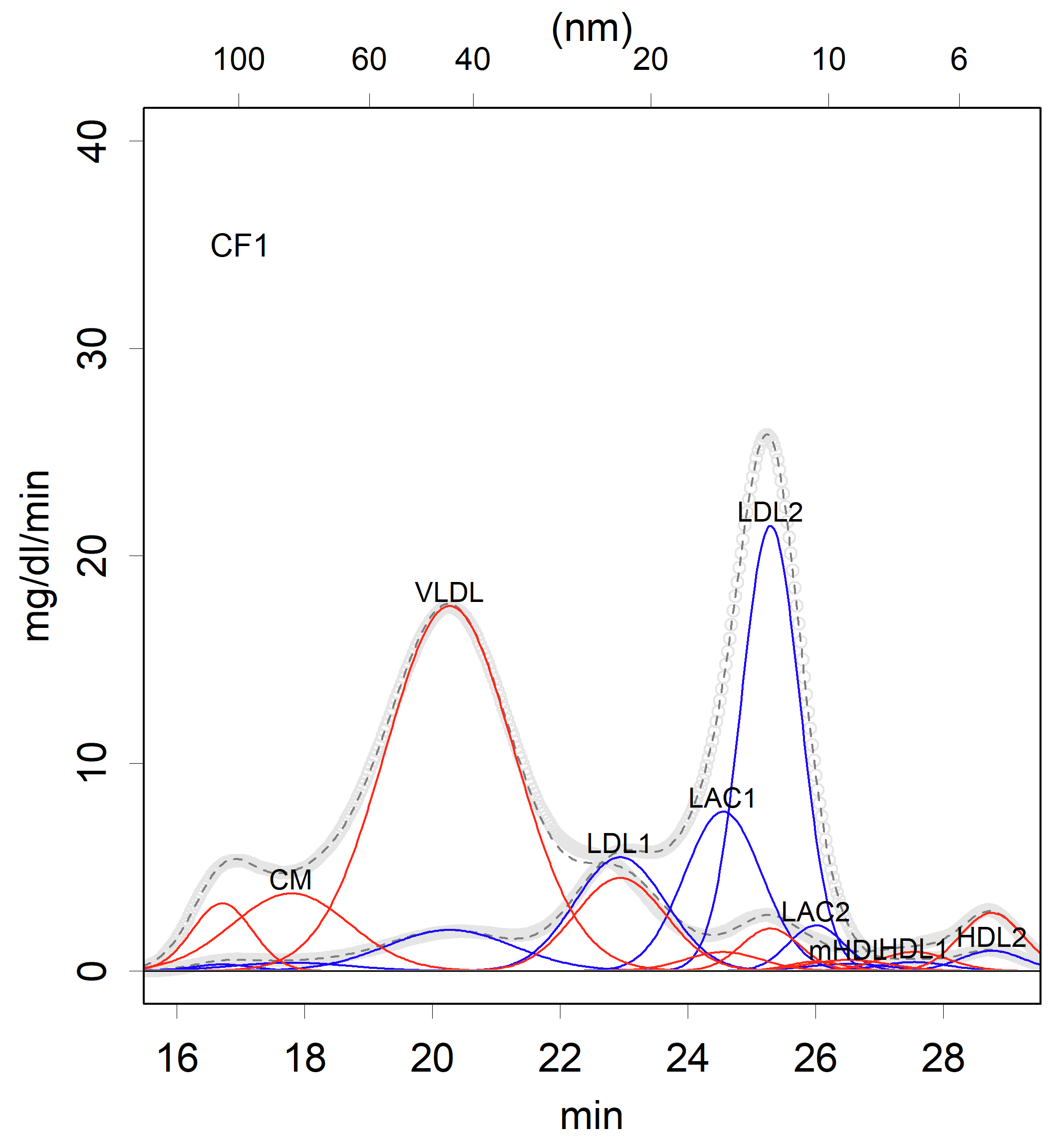

Supplement: S1 Fig — (ZIP) [file pone.0210950.s001.zip › S1_Fig/box/CF1.png]

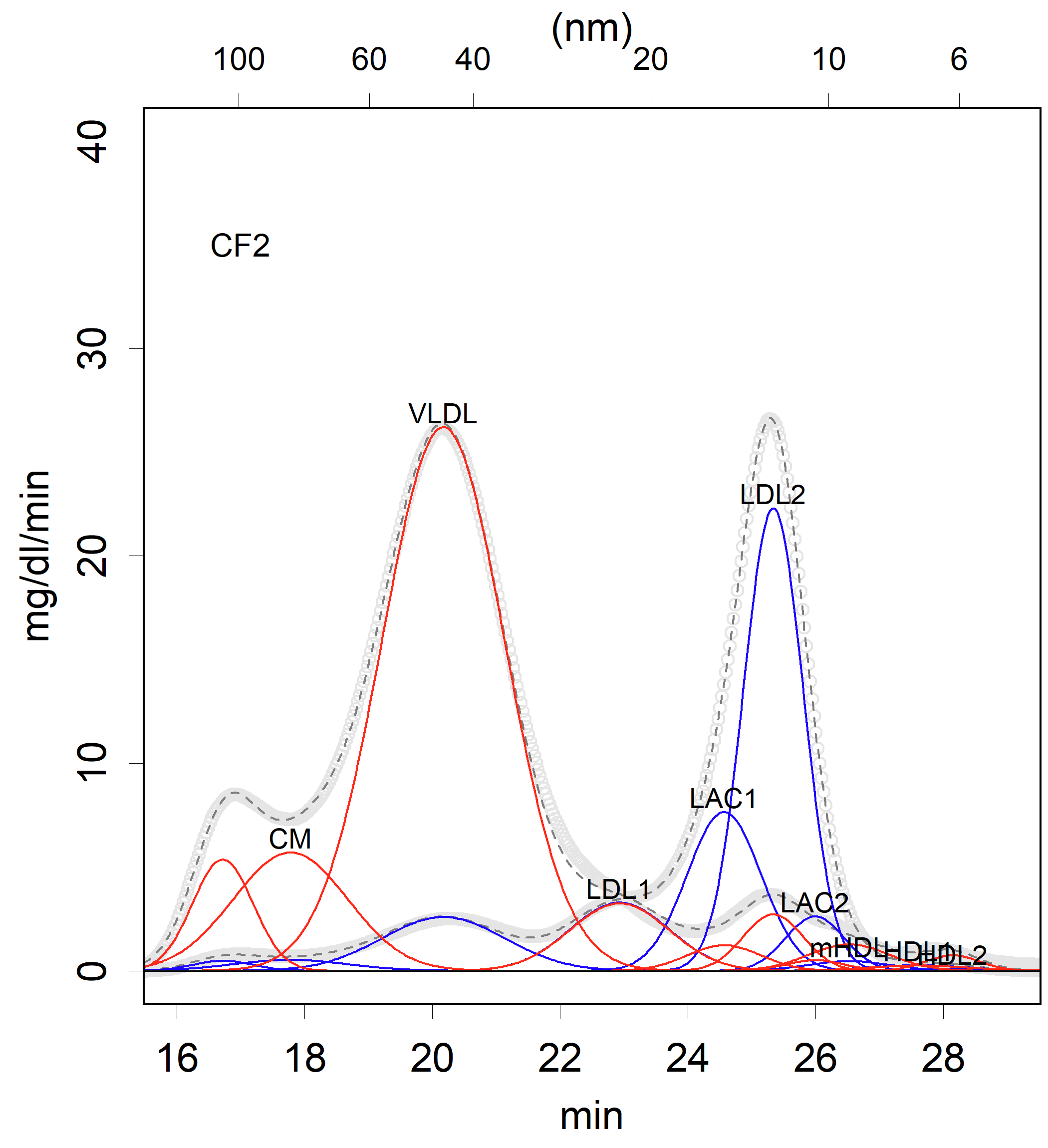

Supplement: S1 Fig — (ZIP) [file pone.0210950.s001.zip › S1_Fig/box/CF2.png]

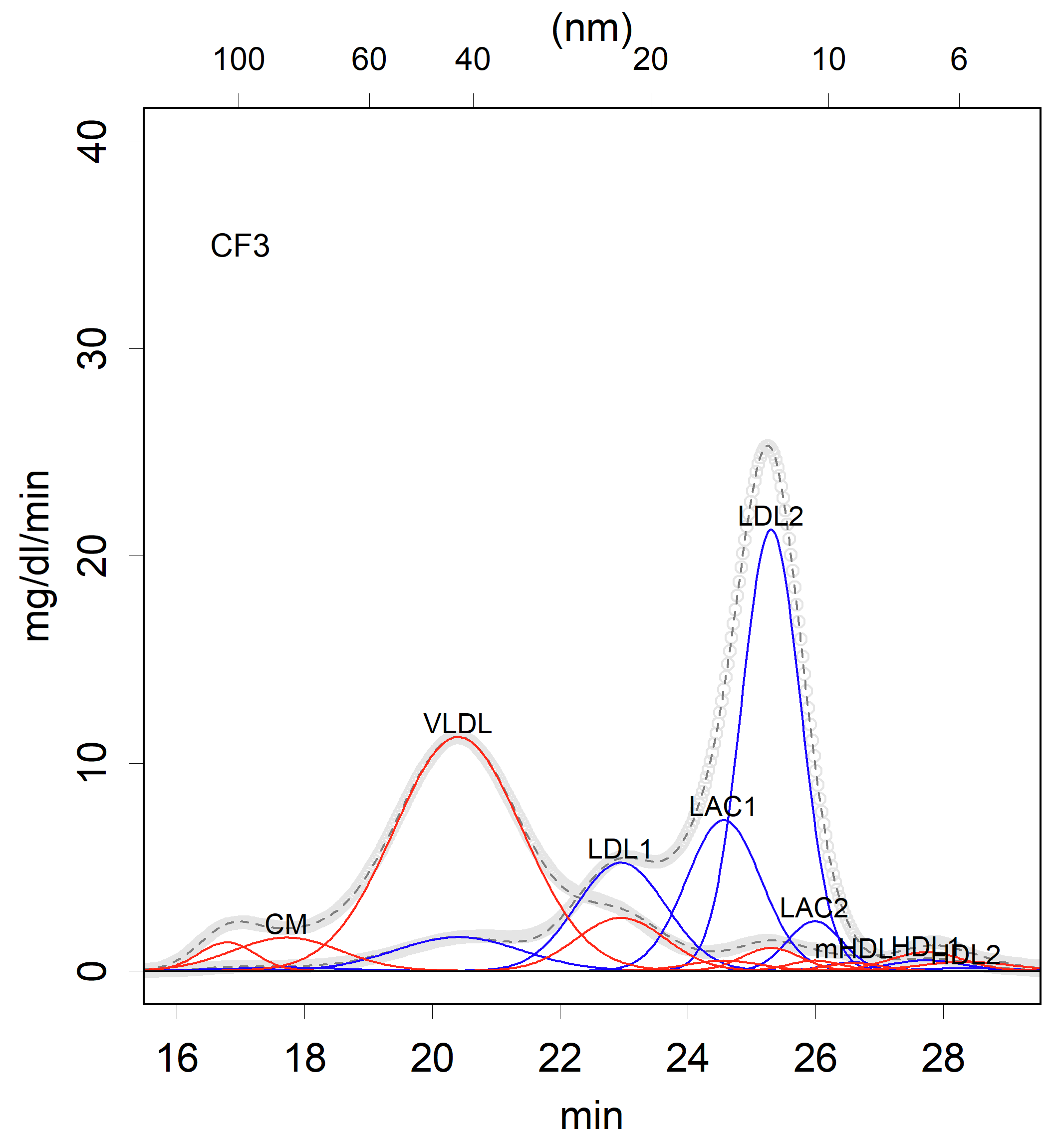

Supplement: S1 Fig — (ZIP) [file pone.0210950.s001.zip › S1_Fig/box/CF3.png]

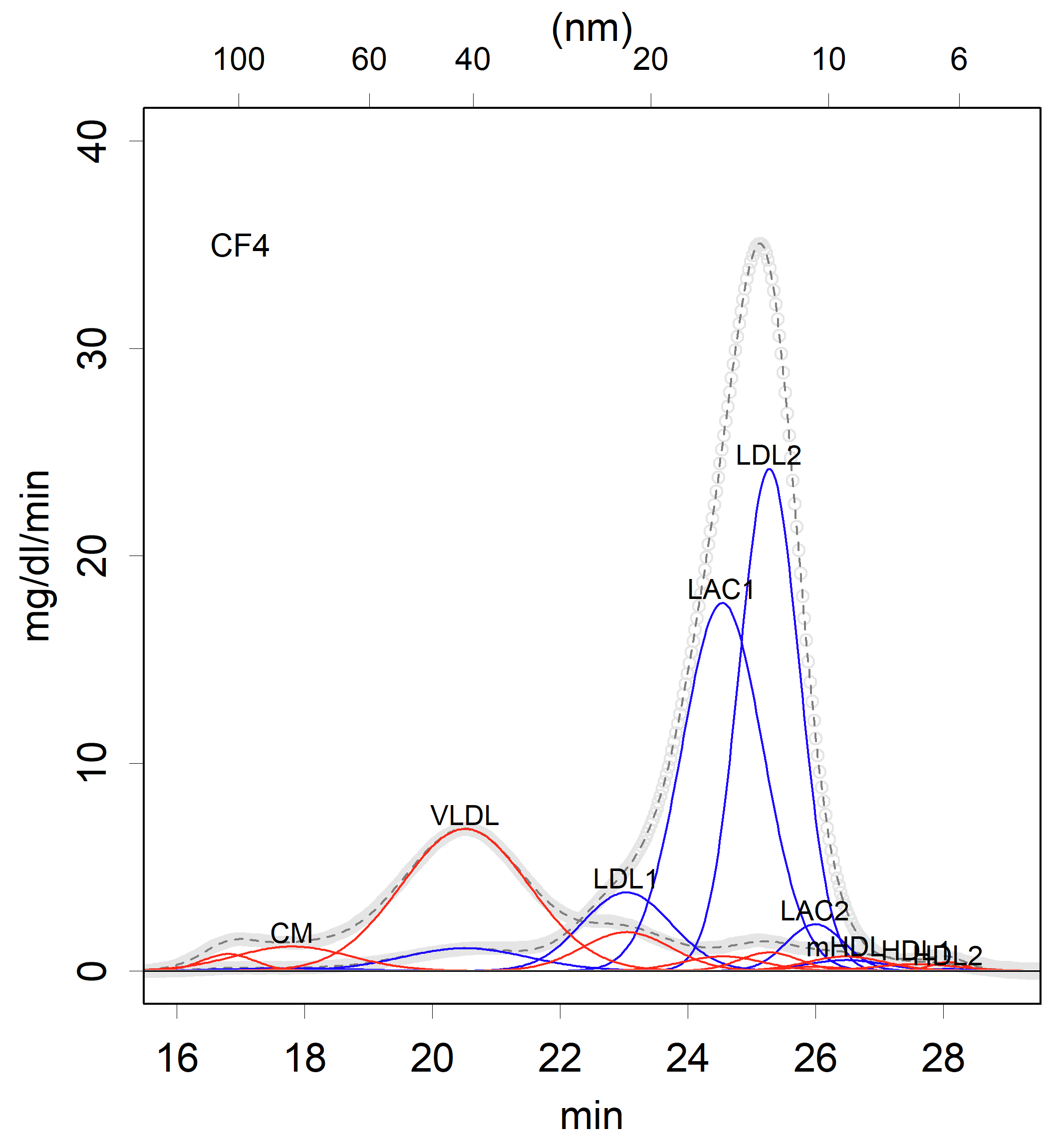

Supplement: S1 Fig — (ZIP) [file pone.0210950.s001.zip › S1_Fig/box/CF4.png]

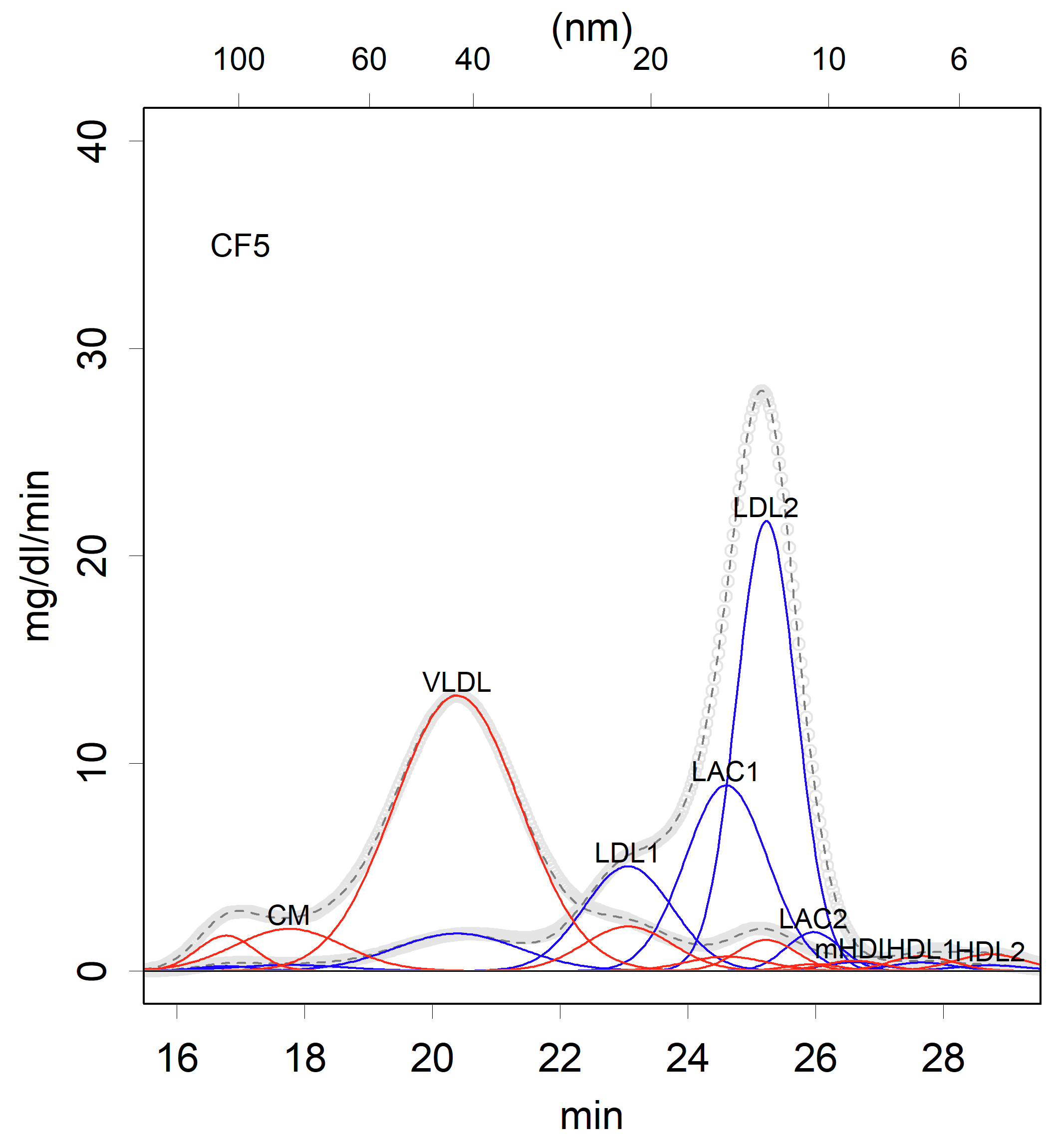

Supplement: S1 Fig — (ZIP) [file pone.0210950.s001.zip › S1_Fig/box/CF5.png]

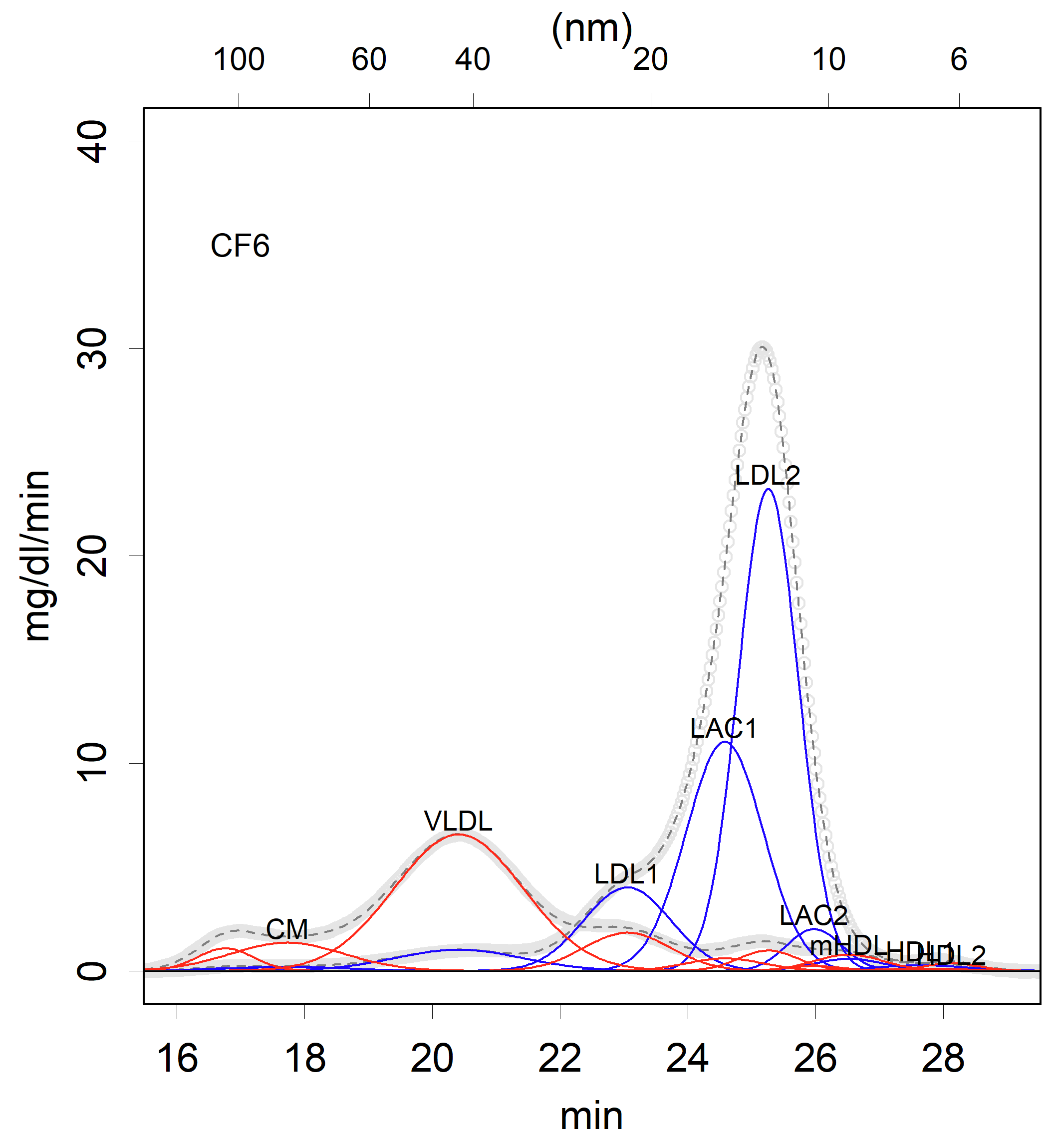

Supplement: S1 Fig — (ZIP) [file pone.0210950.s001.zip › S1_Fig/box/CF6.png]

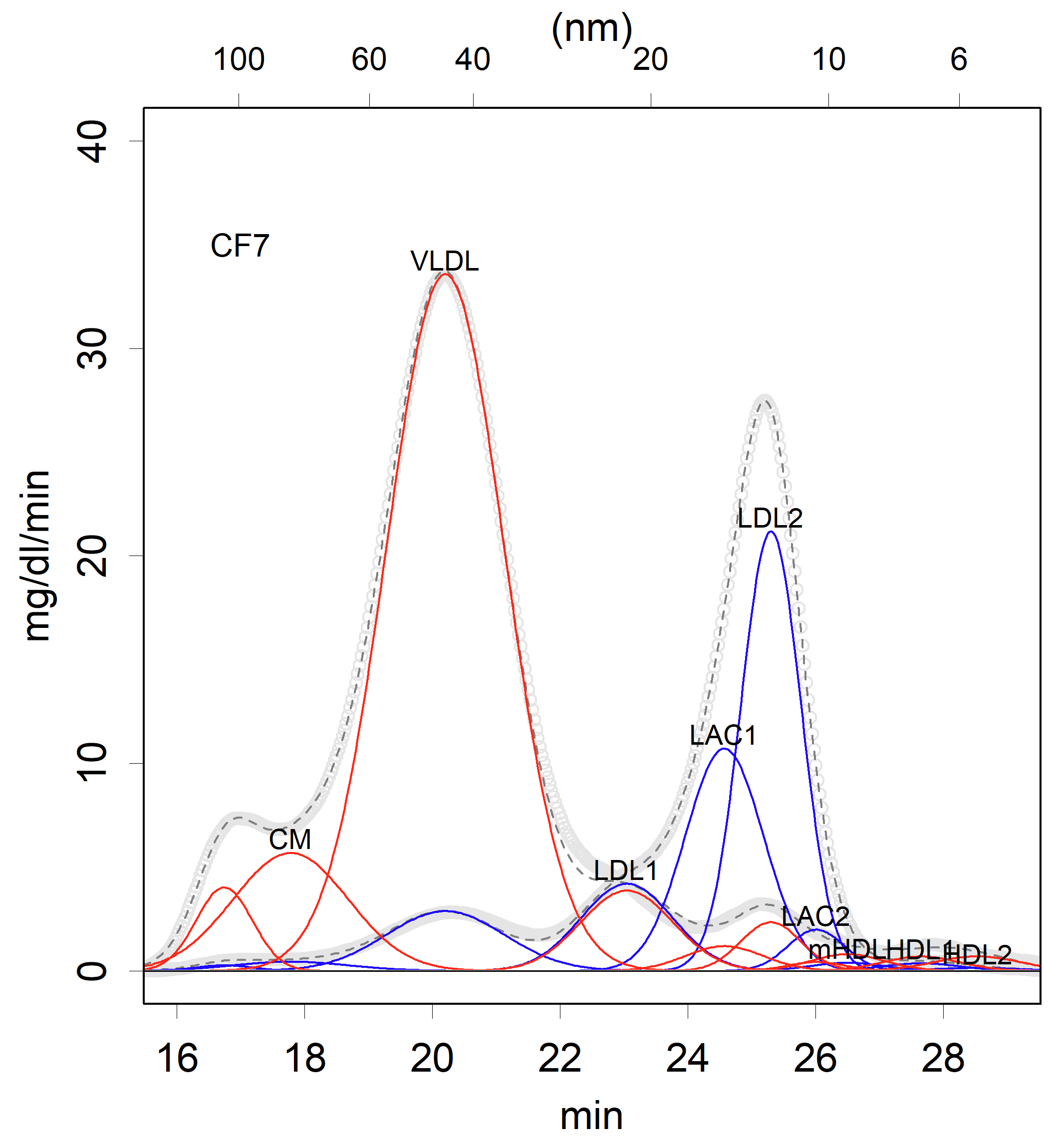

Supplement: S1 Fig — (ZIP) [file pone.0210950.s001.zip › S1_Fig/box/CF7.png]

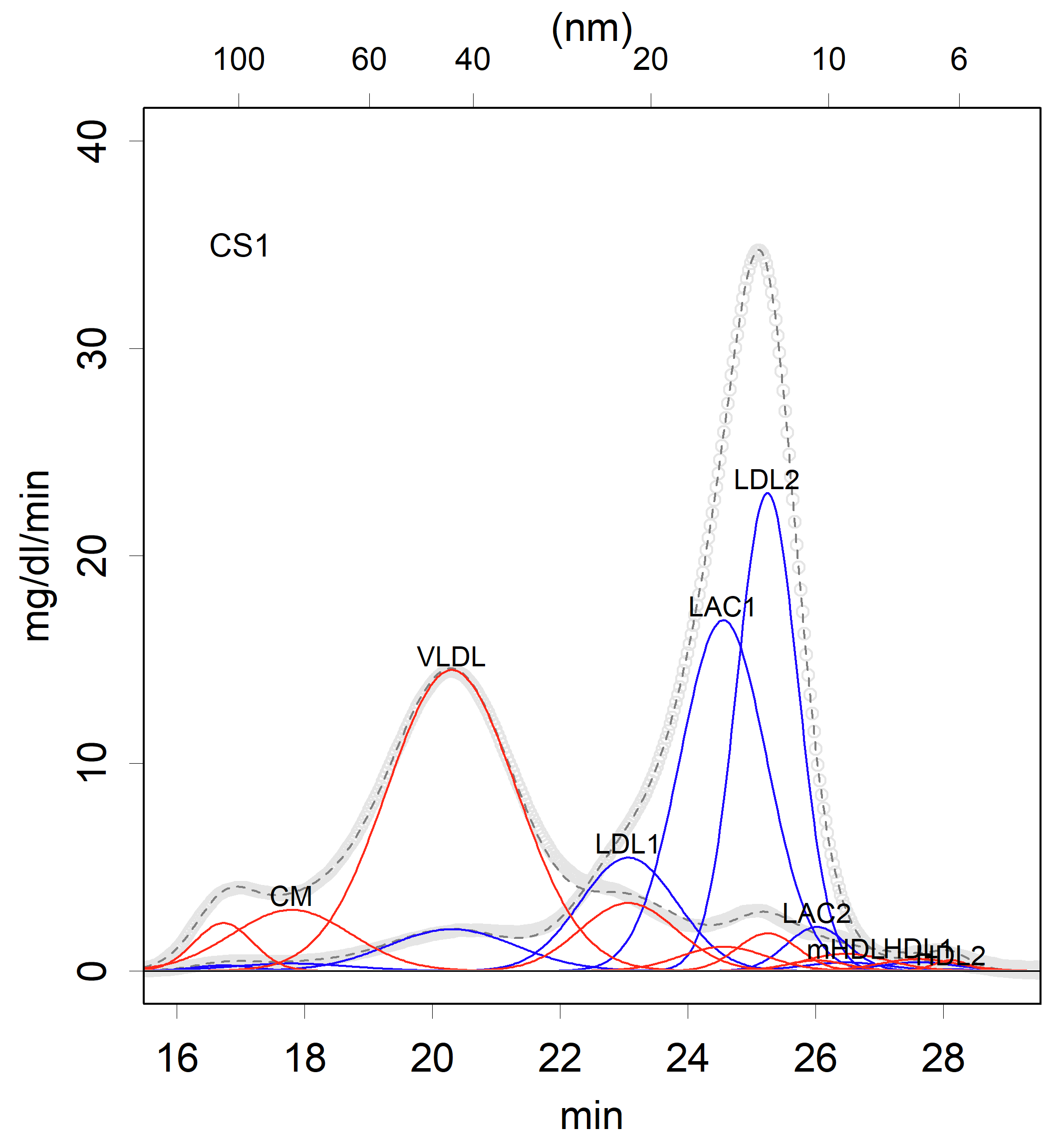

Supplement: S1 Fig — (ZIP) [file pone.0210950.s001.zip › S1_Fig/box/CS1.png]

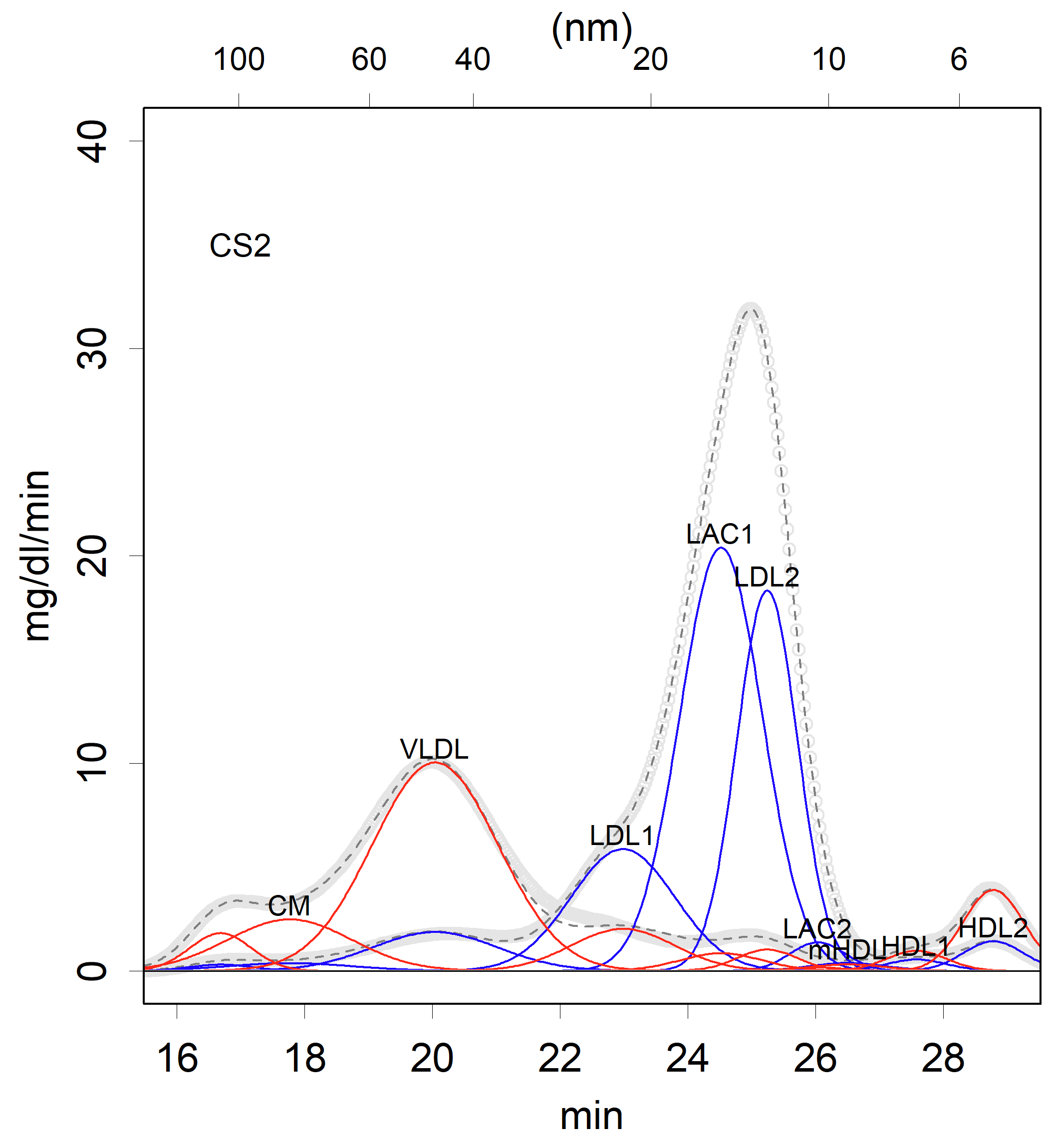

Supplement: S1 Fig — (ZIP) [file pone.0210950.s001.zip › S1_Fig/box/CS2.png]

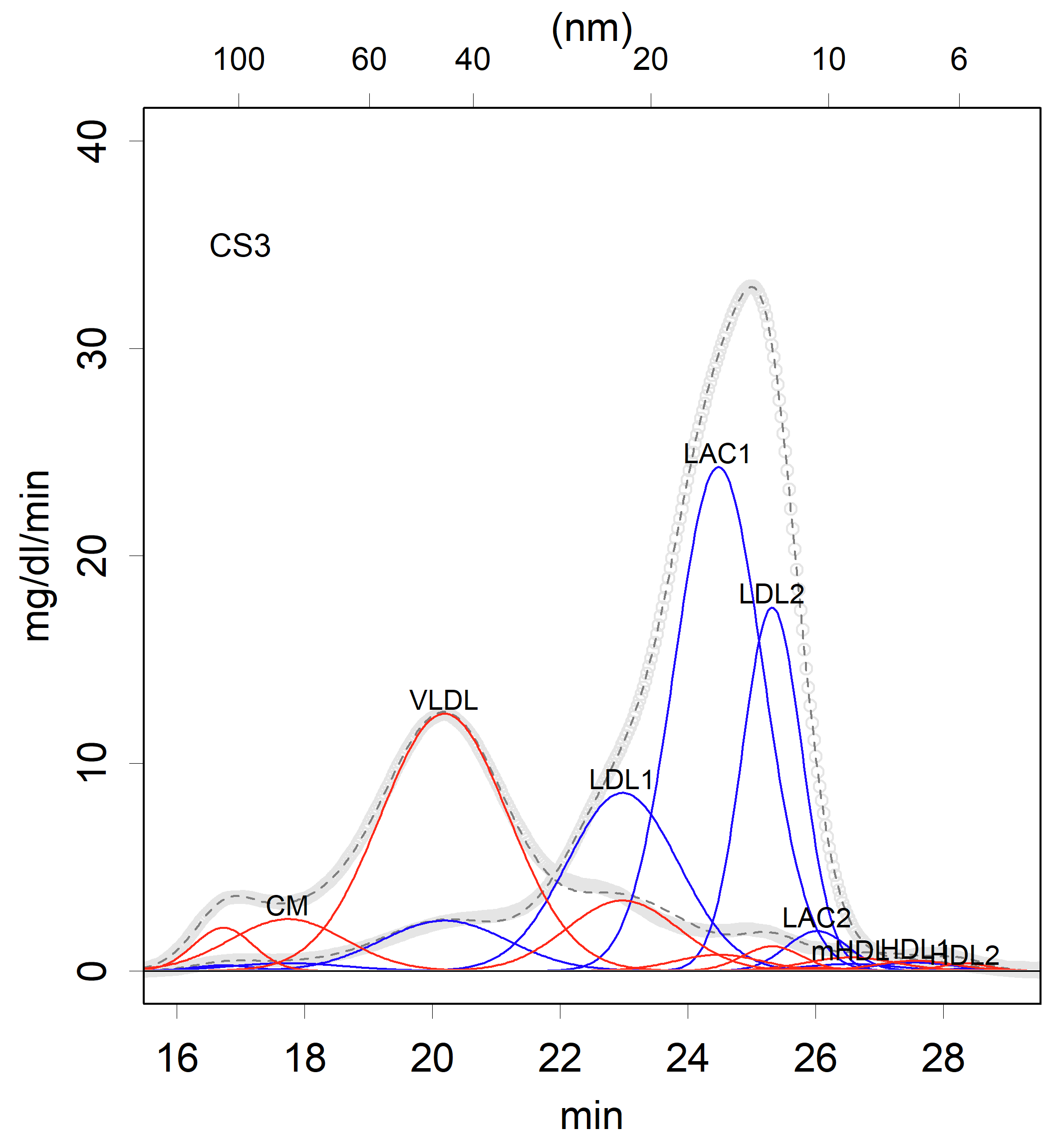

Supplement: S1 Fig — (ZIP) [file pone.0210950.s001.zip › S1_Fig/box/CS3.png]

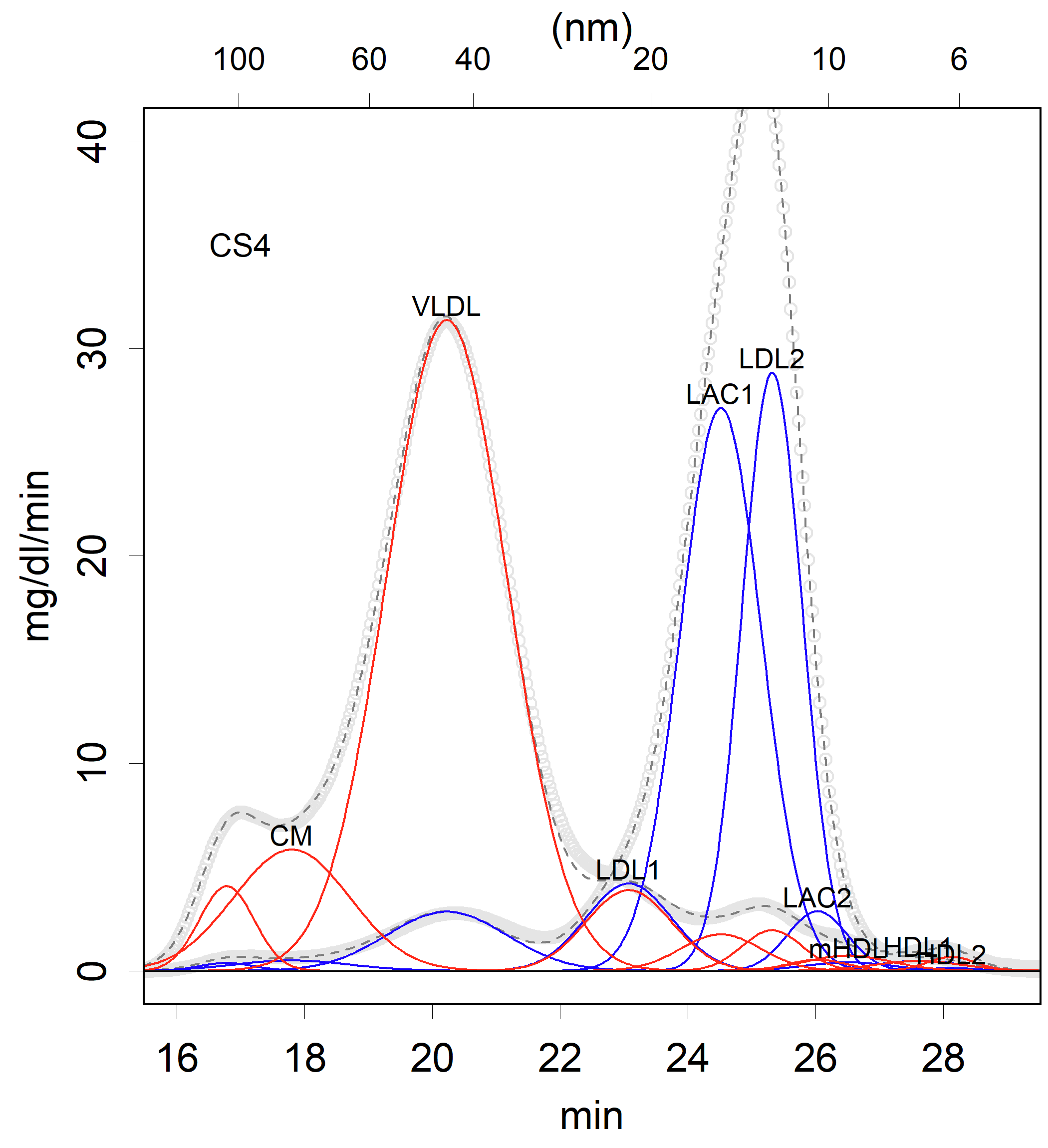

Supplement: S1 Fig — (ZIP) [file pone.0210950.s001.zip › S1_Fig/box/CS4.png]

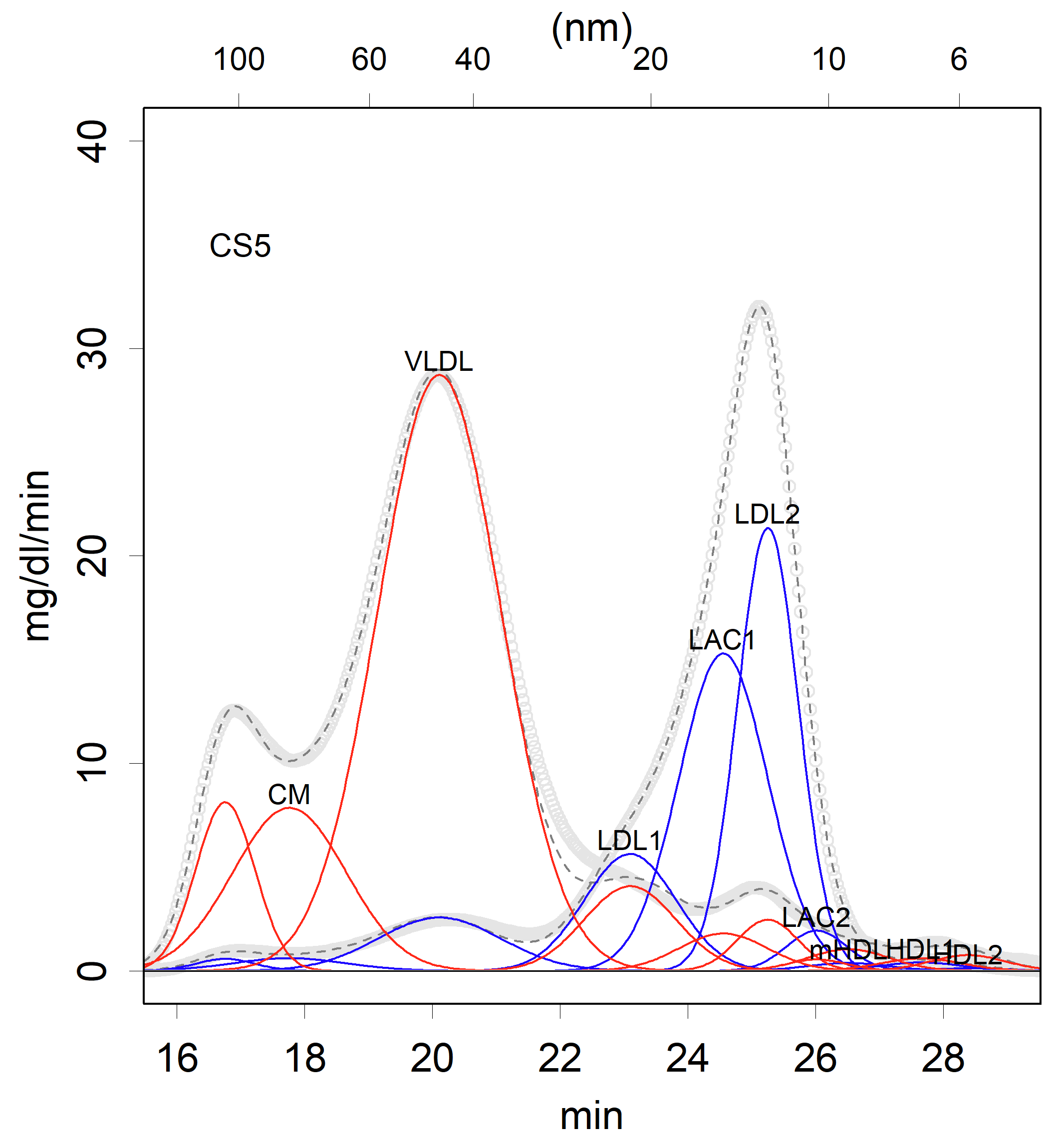

Supplement: S1 Fig — (ZIP) [file pone.0210950.s001.zip › S1_Fig/box/CS5.png]

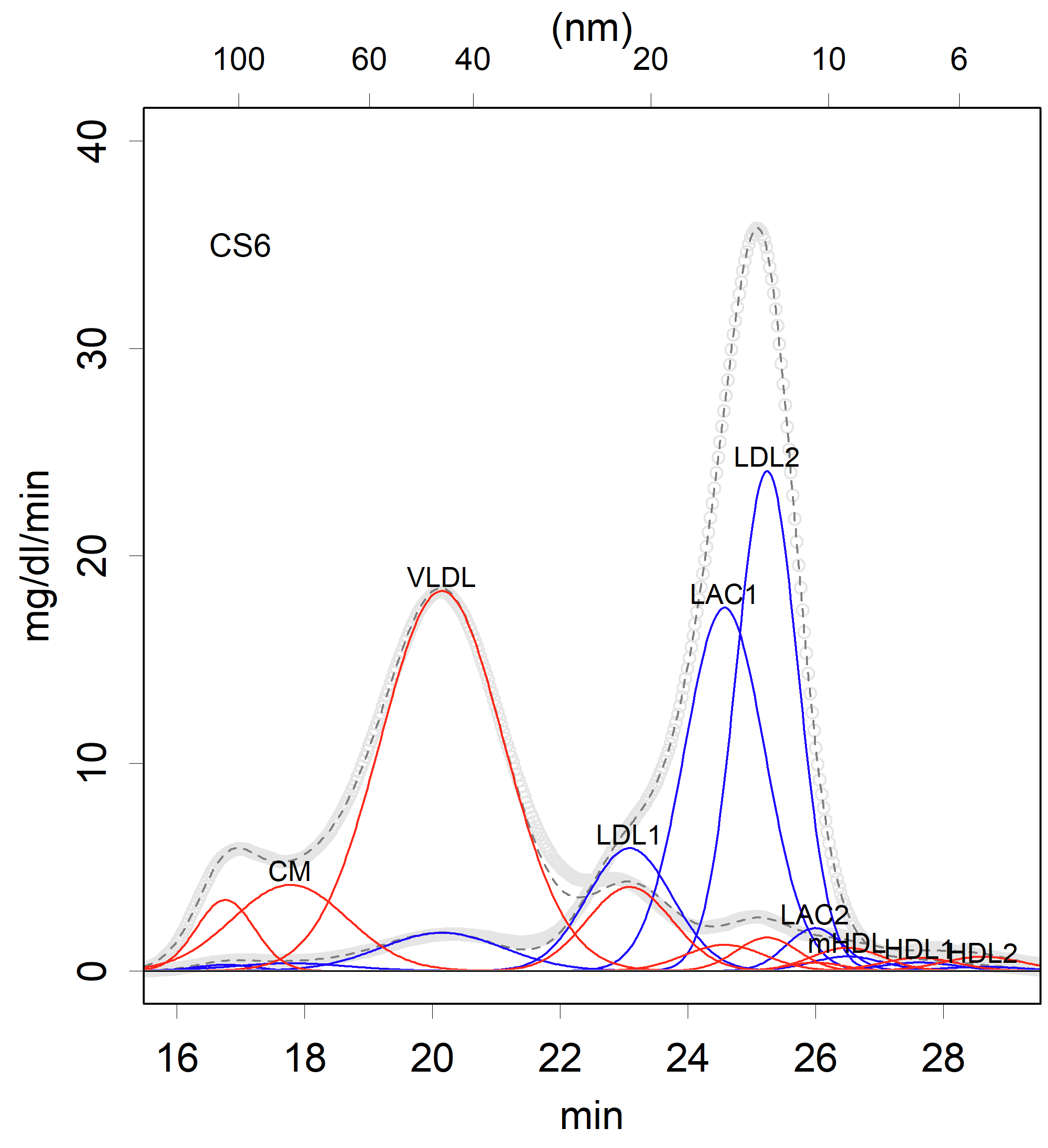

Supplement: S1 Fig — (ZIP) [file pone.0210950.s001.zip › S1_Fig/box/CS6.png]

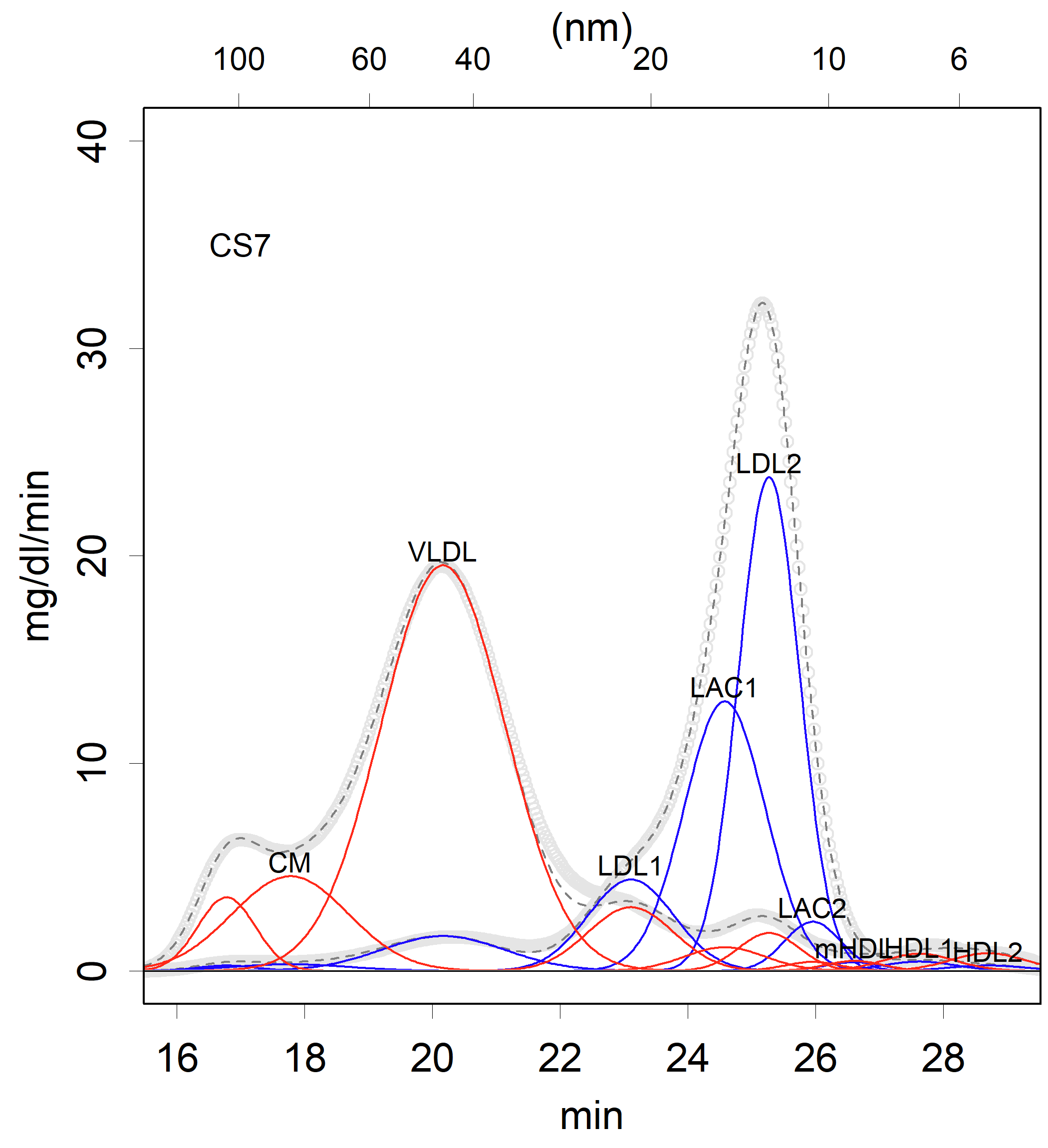

Supplement: S1 Fig — (ZIP) [file pone.0210950.s001.zip › S1_Fig/box/CS7.png]

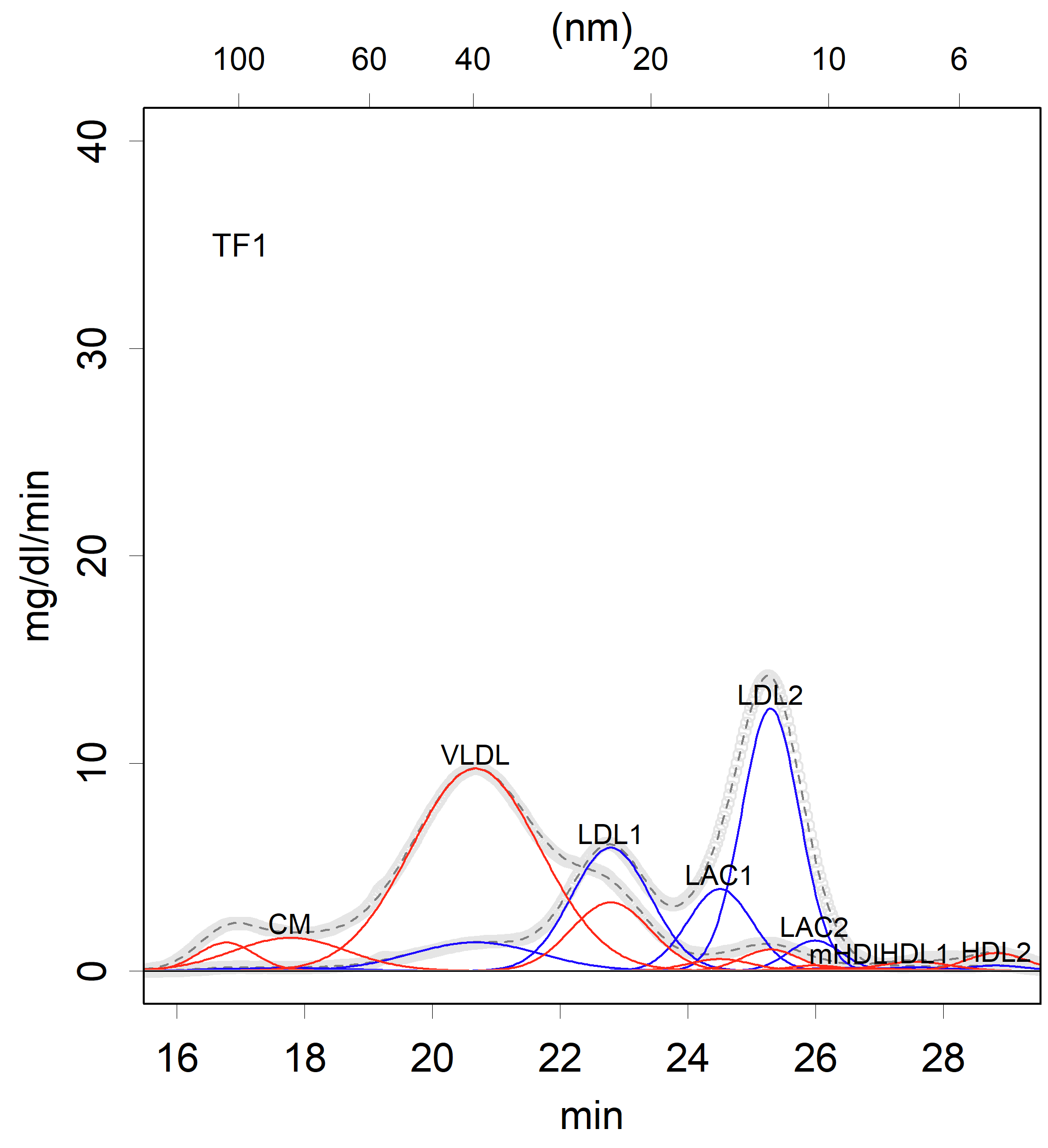

Supplement: S1 Fig — (ZIP) [file pone.0210950.s001.zip › S1_Fig/box/TF1.png]

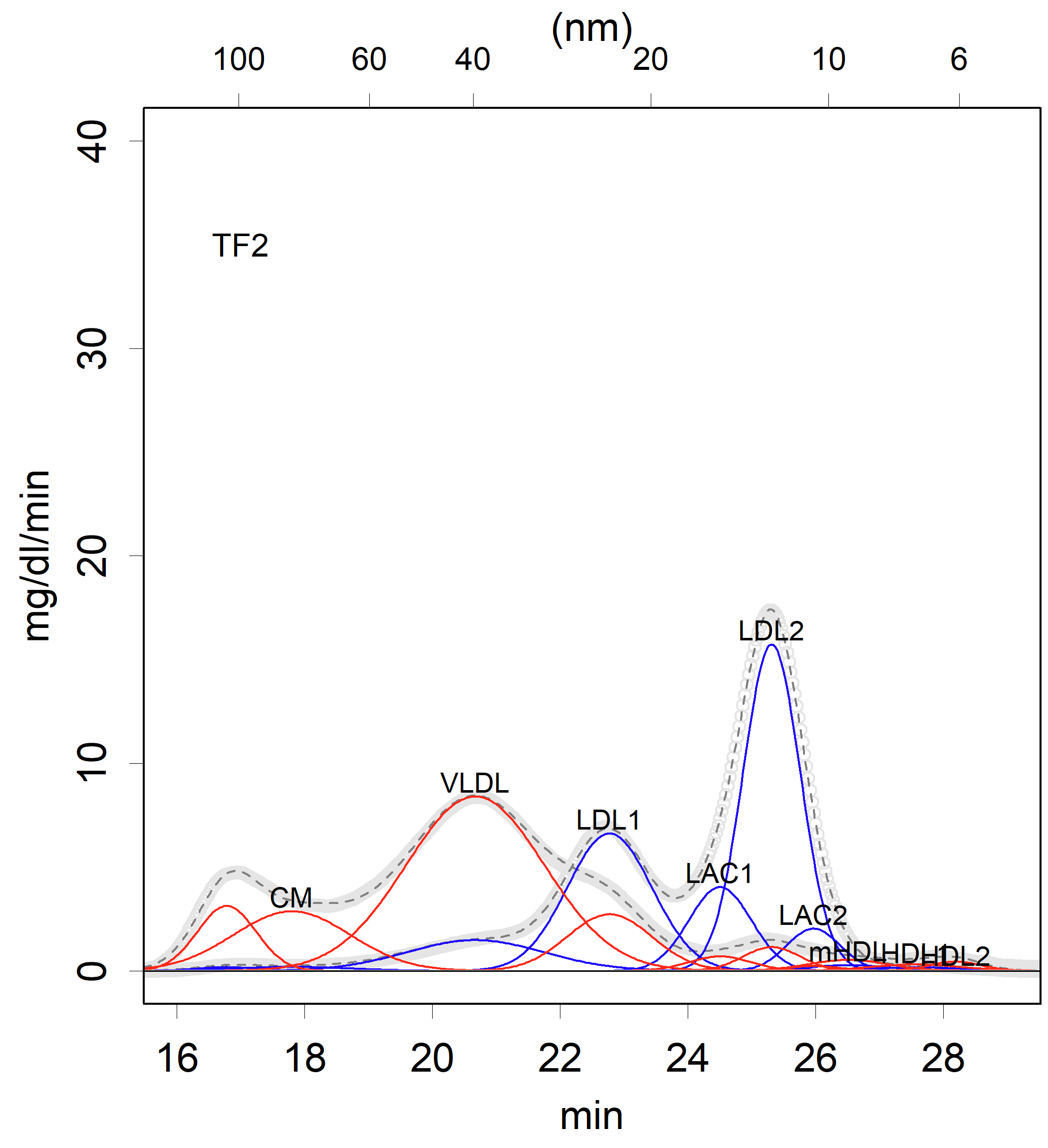

Supplement: S1 Fig — (ZIP) [file pone.0210950.s001.zip › S1_Fig/box/TF2.png]

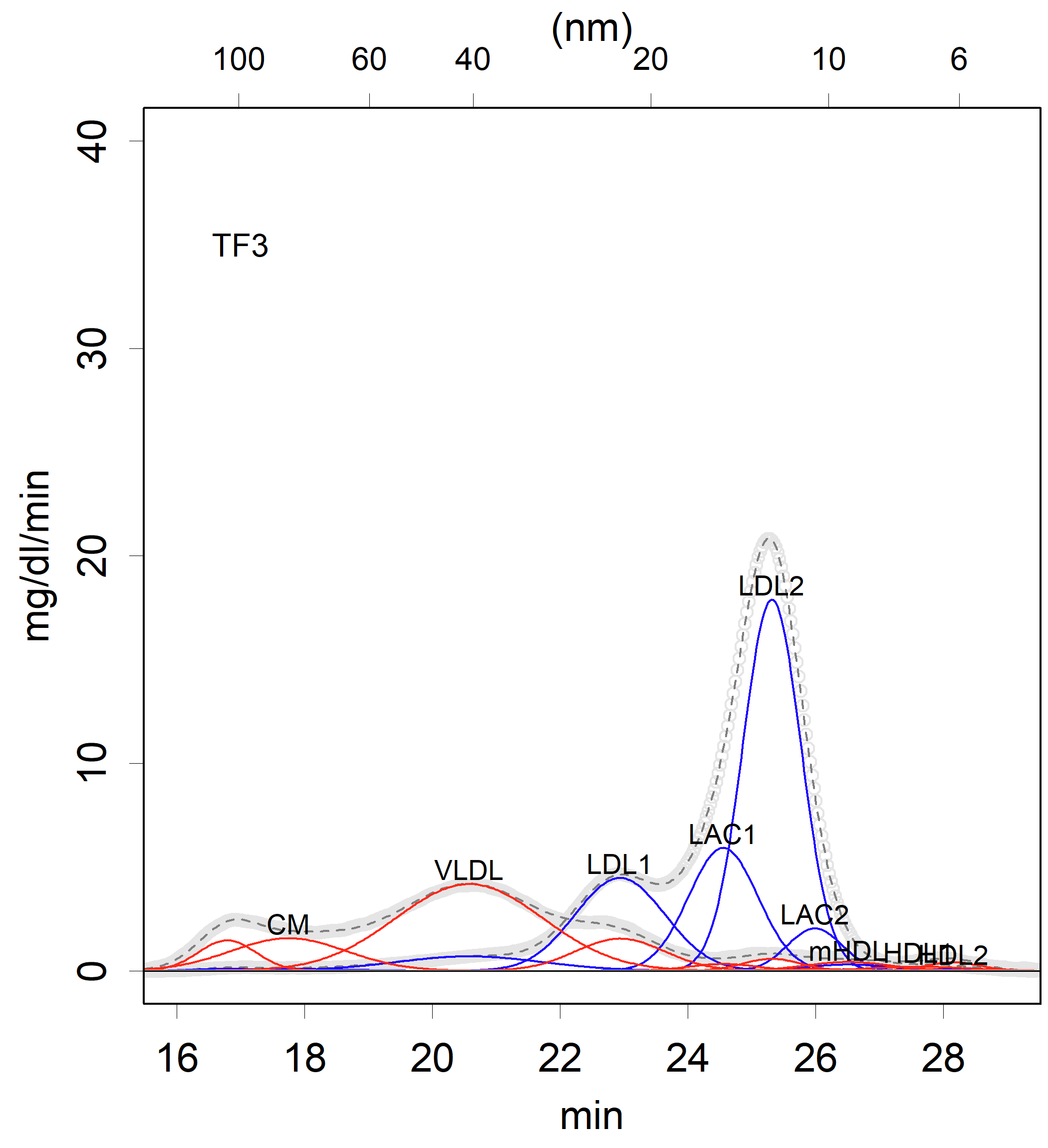

Supplement: S1 Fig — (ZIP) [file pone.0210950.s001.zip › S1_Fig/box/TF3.png]

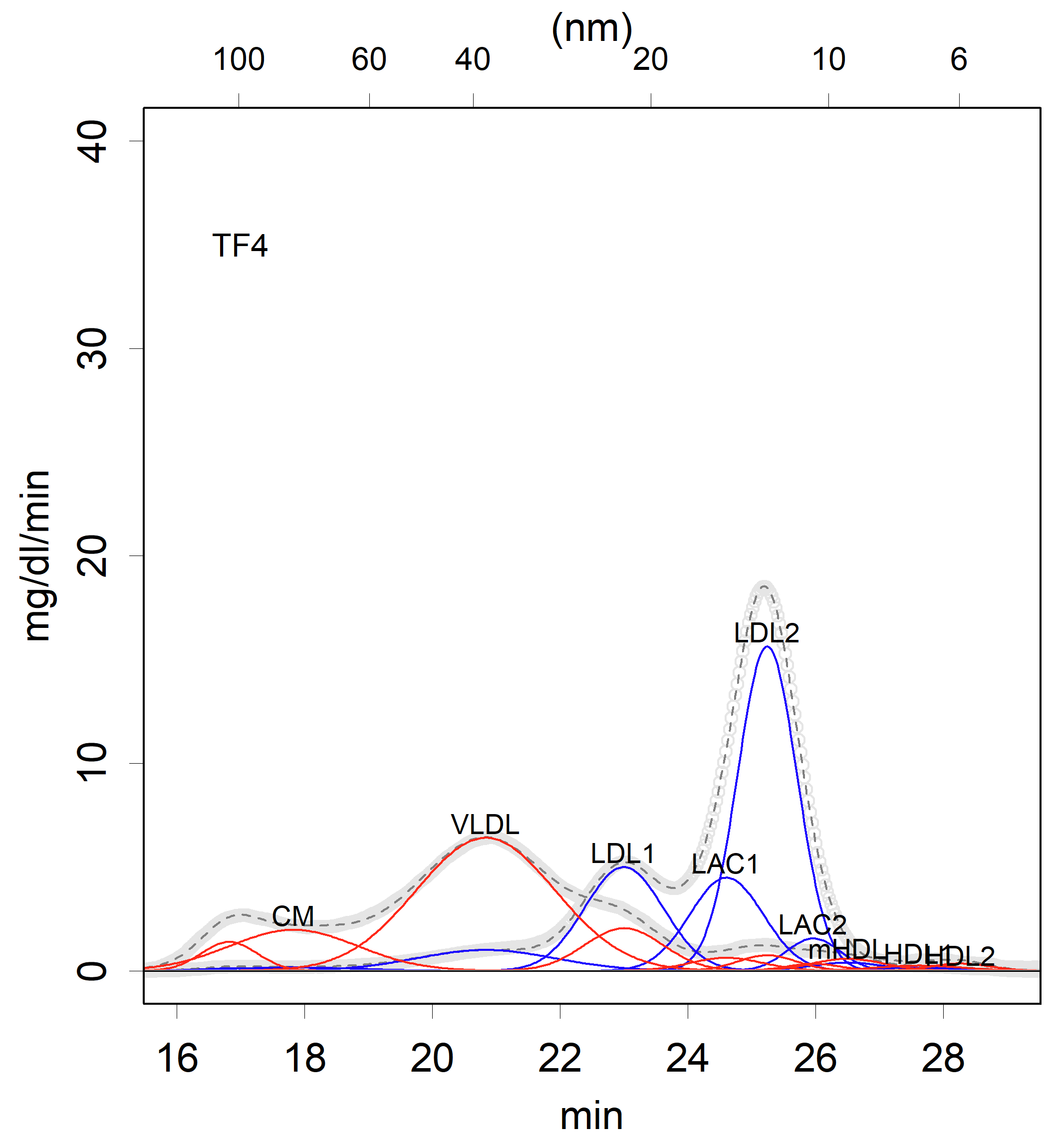

Supplement: S1 Fig — (ZIP) [file pone.0210950.s001.zip › S1_Fig/box/TF4.png]

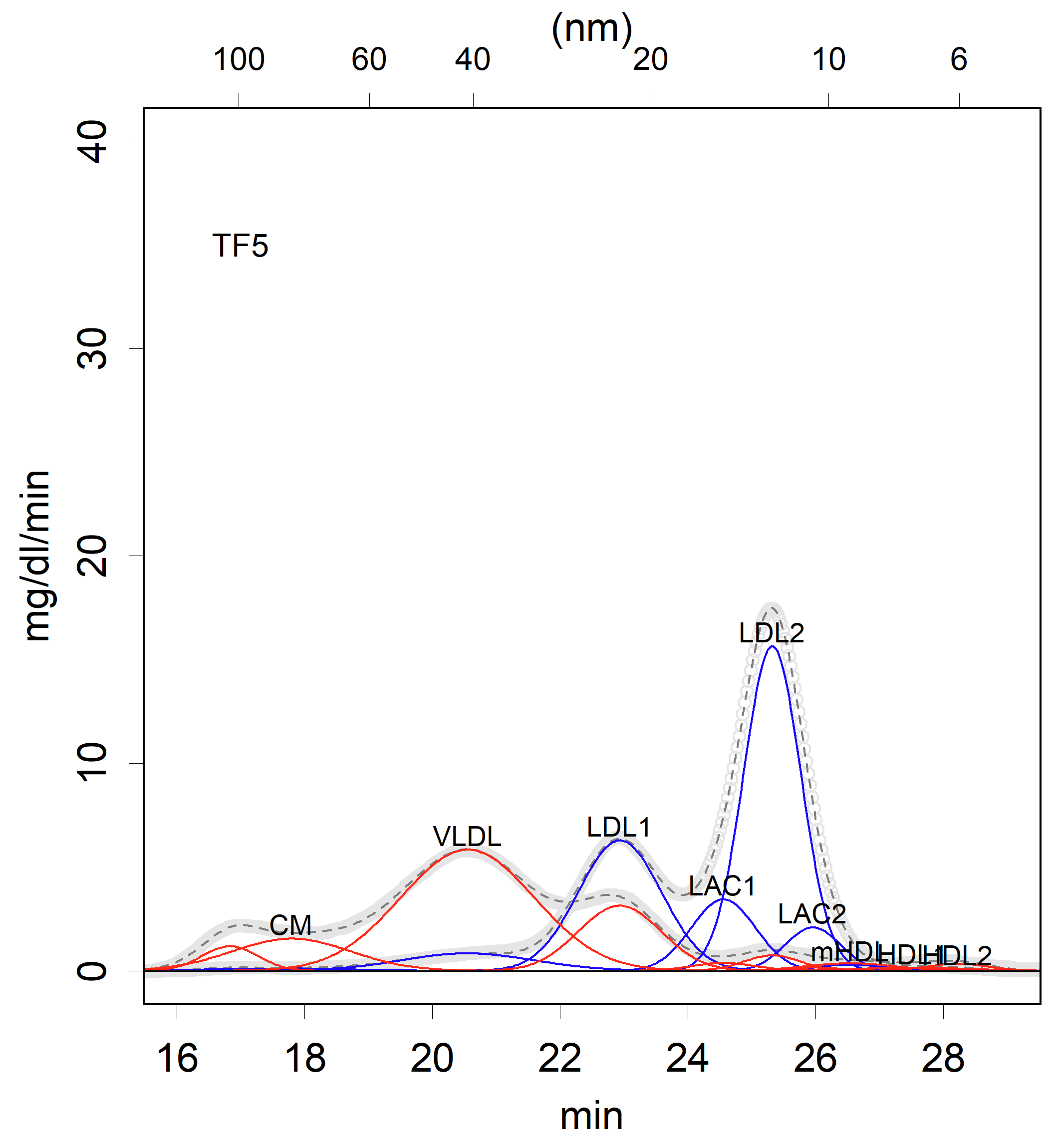

Supplement: S1 Fig — (ZIP) [file pone.0210950.s001.zip › S1_Fig/box/TF5.png]

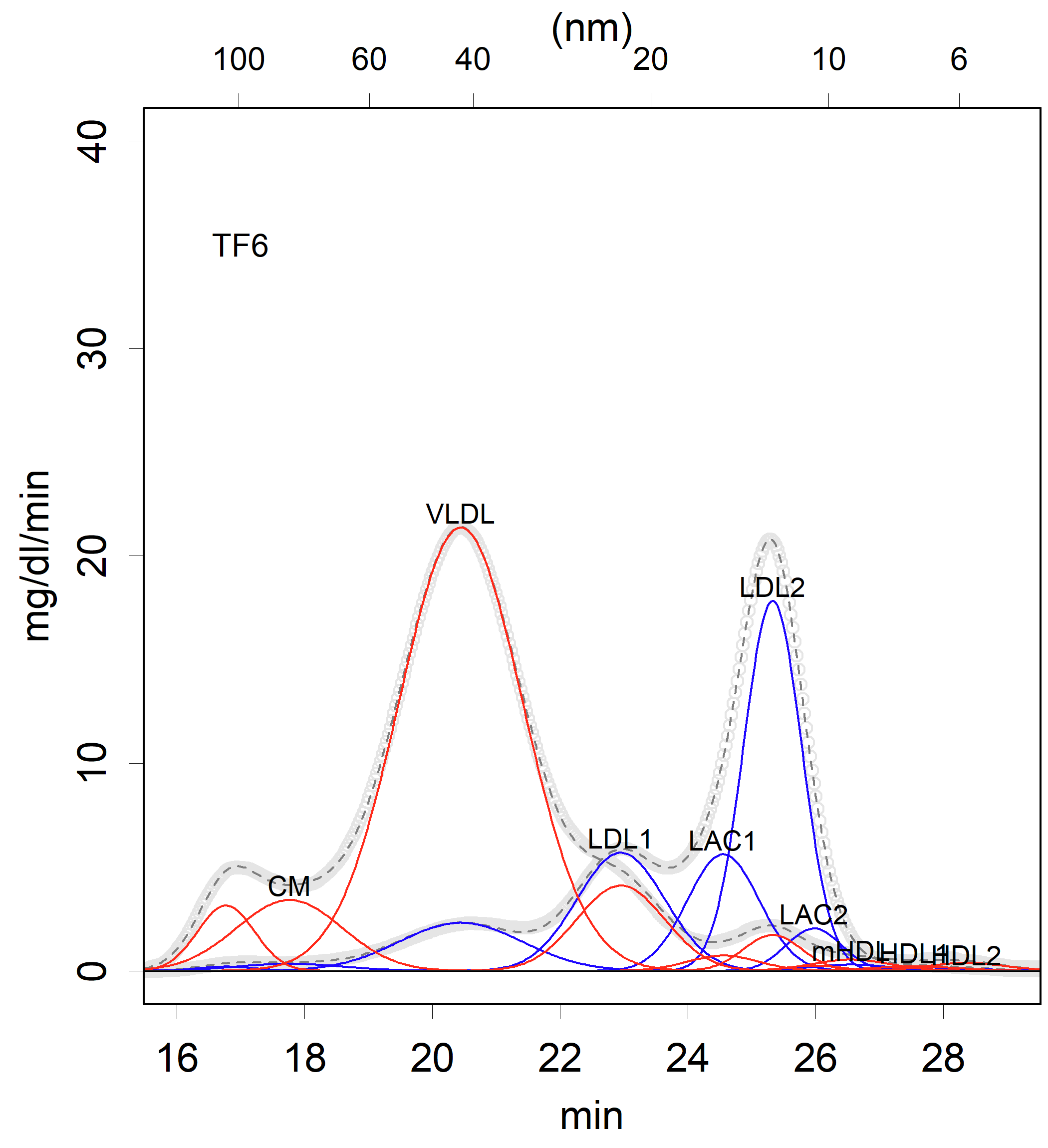

Supplement: S1 Fig — (ZIP) [file pone.0210950.s001.zip › S1_Fig/box/TF6.png]

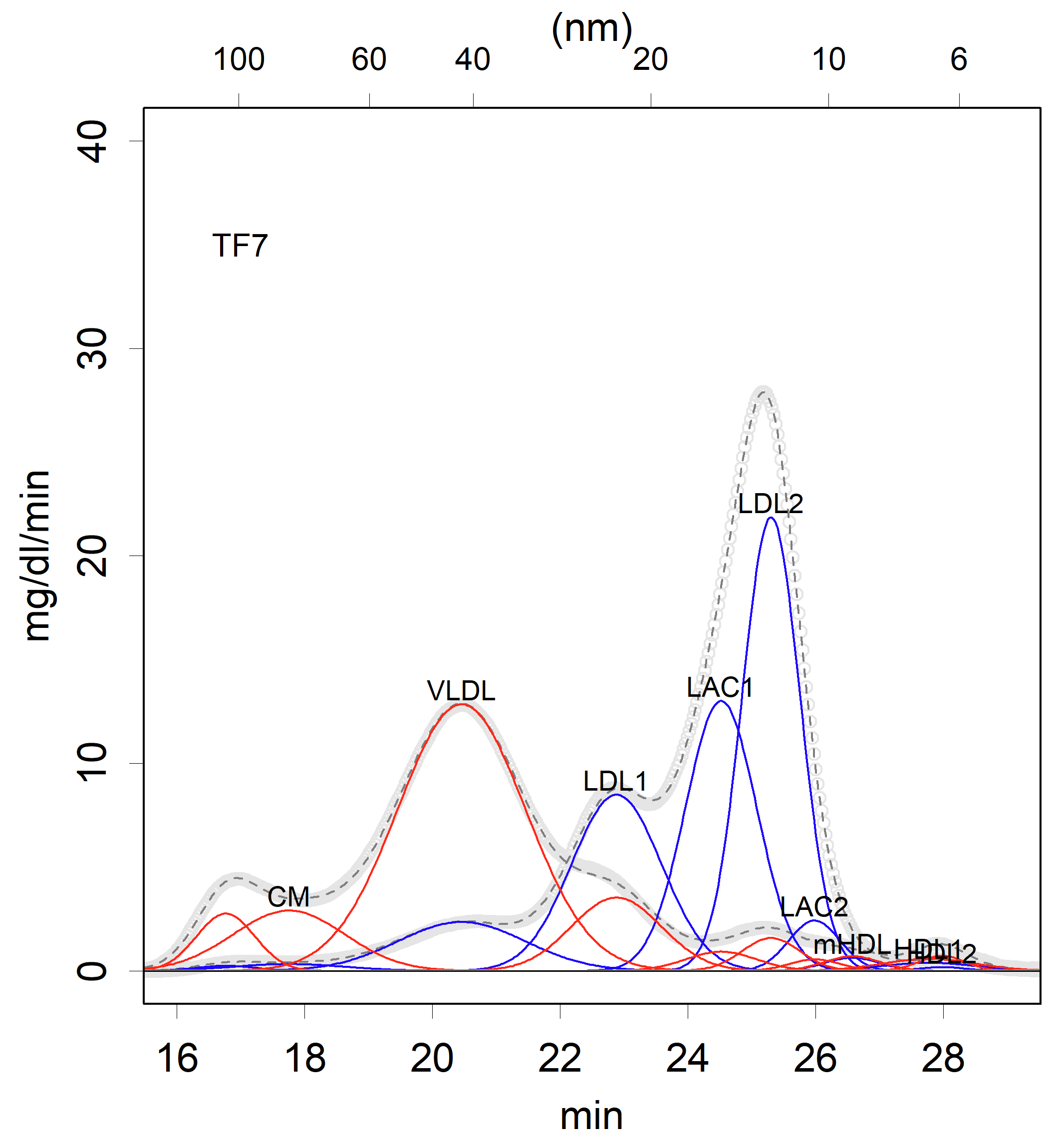

Supplement: S1 Fig — (ZIP) [file pone.0210950.s001.zip › S1_Fig/box/TF7.png]

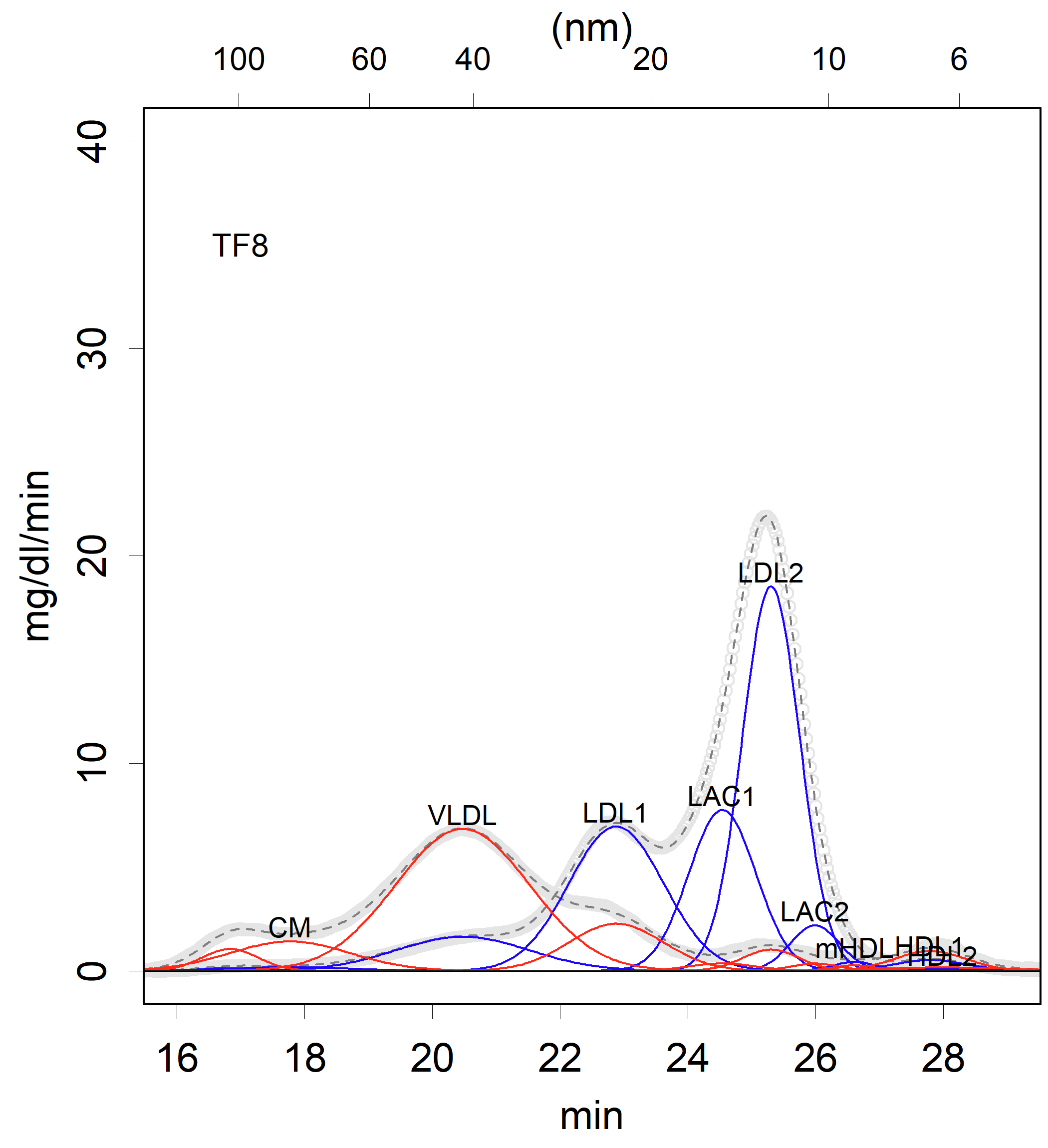

Supplement: S1 Fig — (ZIP) [file pone.0210950.s001.zip › S1_Fig/box/TF8.png]

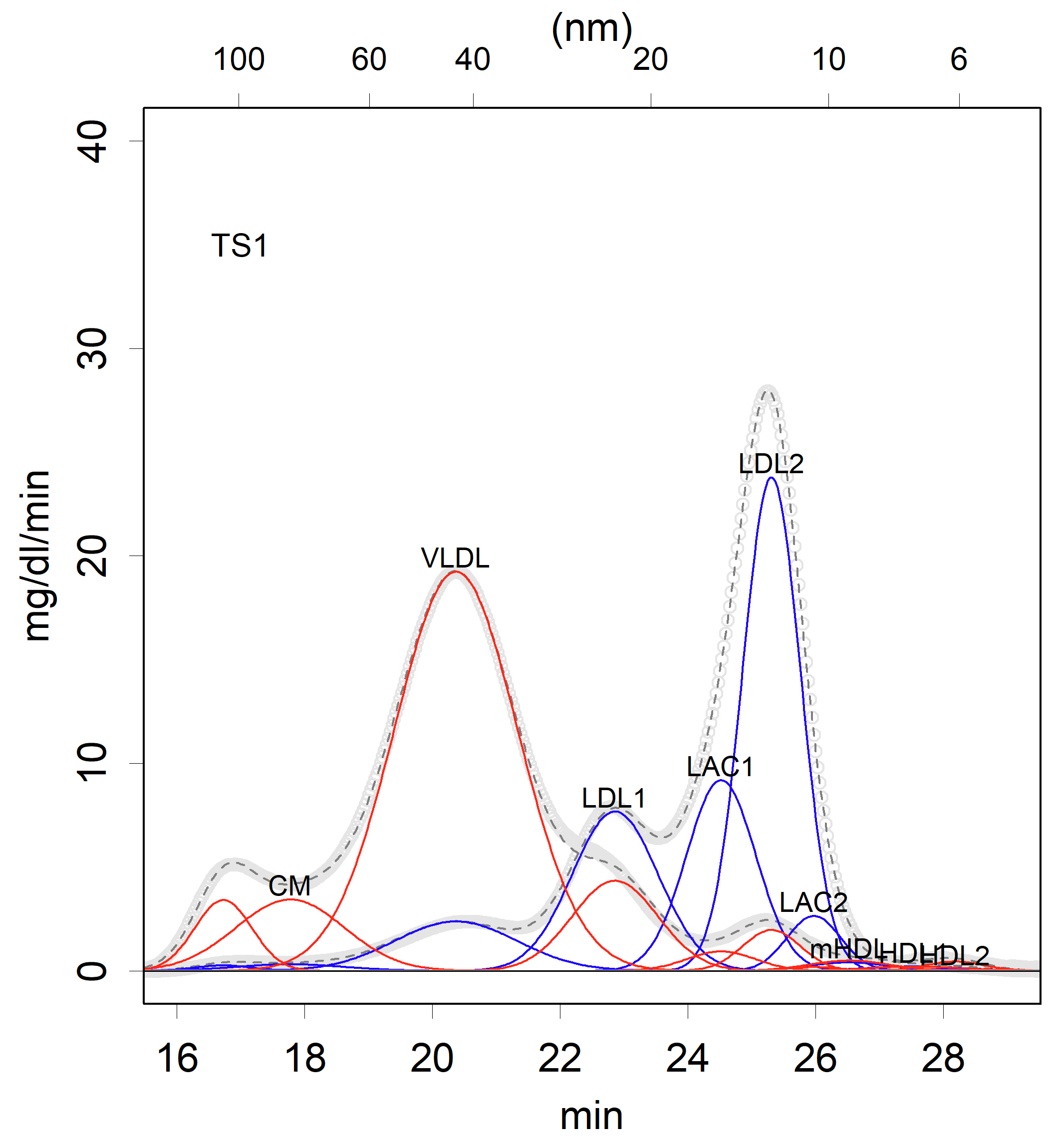

Supplement: S1 Fig — (ZIP) [file pone.0210950.s001.zip › S1_Fig/box/TS1.png]

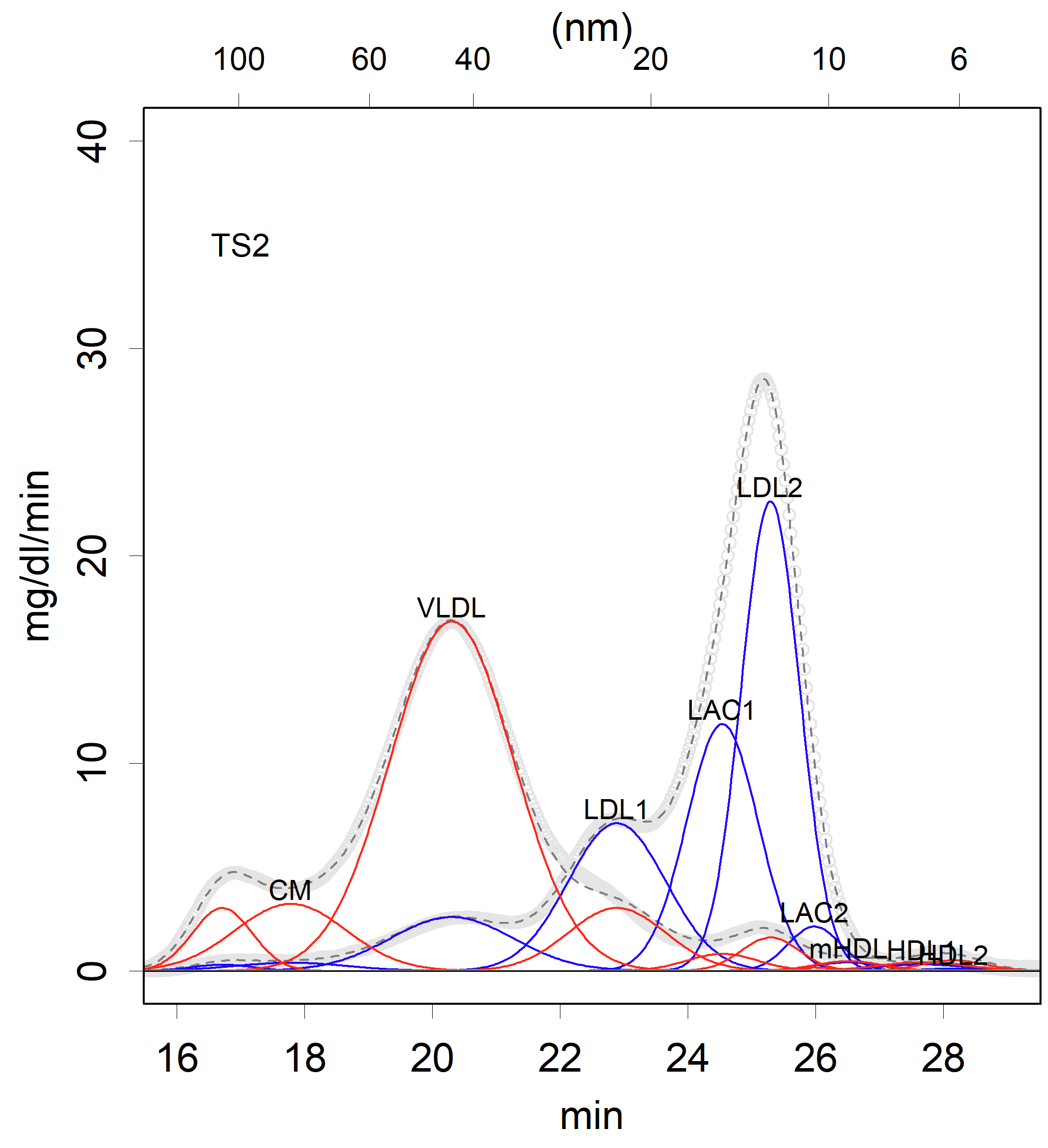

Supplement: S1 Fig — (ZIP) [file pone.0210950.s001.zip › S1_Fig/box/TS2.png]

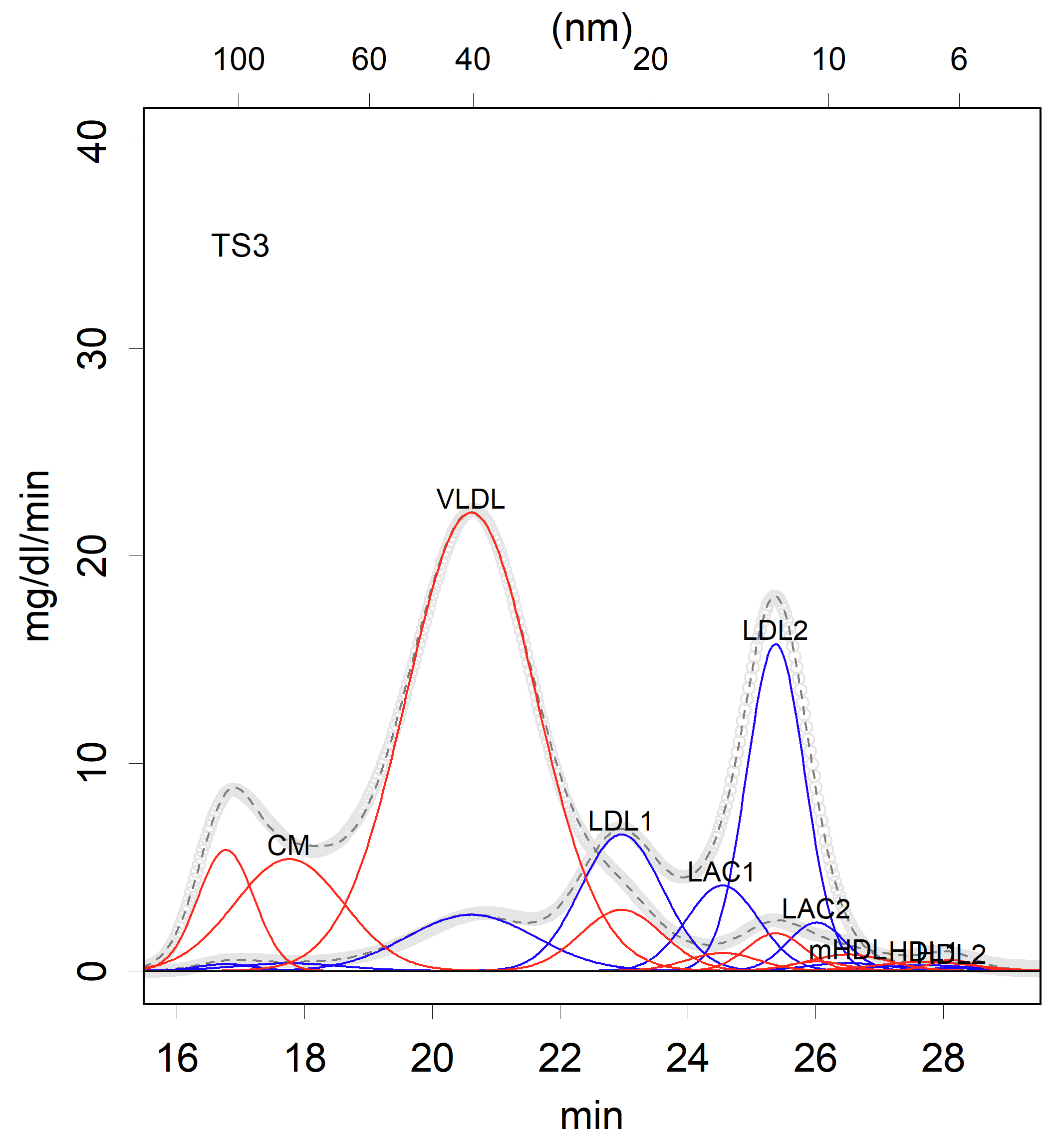

Supplement: S1 Fig — (ZIP) [file pone.0210950.s001.zip › S1_Fig/box/TS3.png]

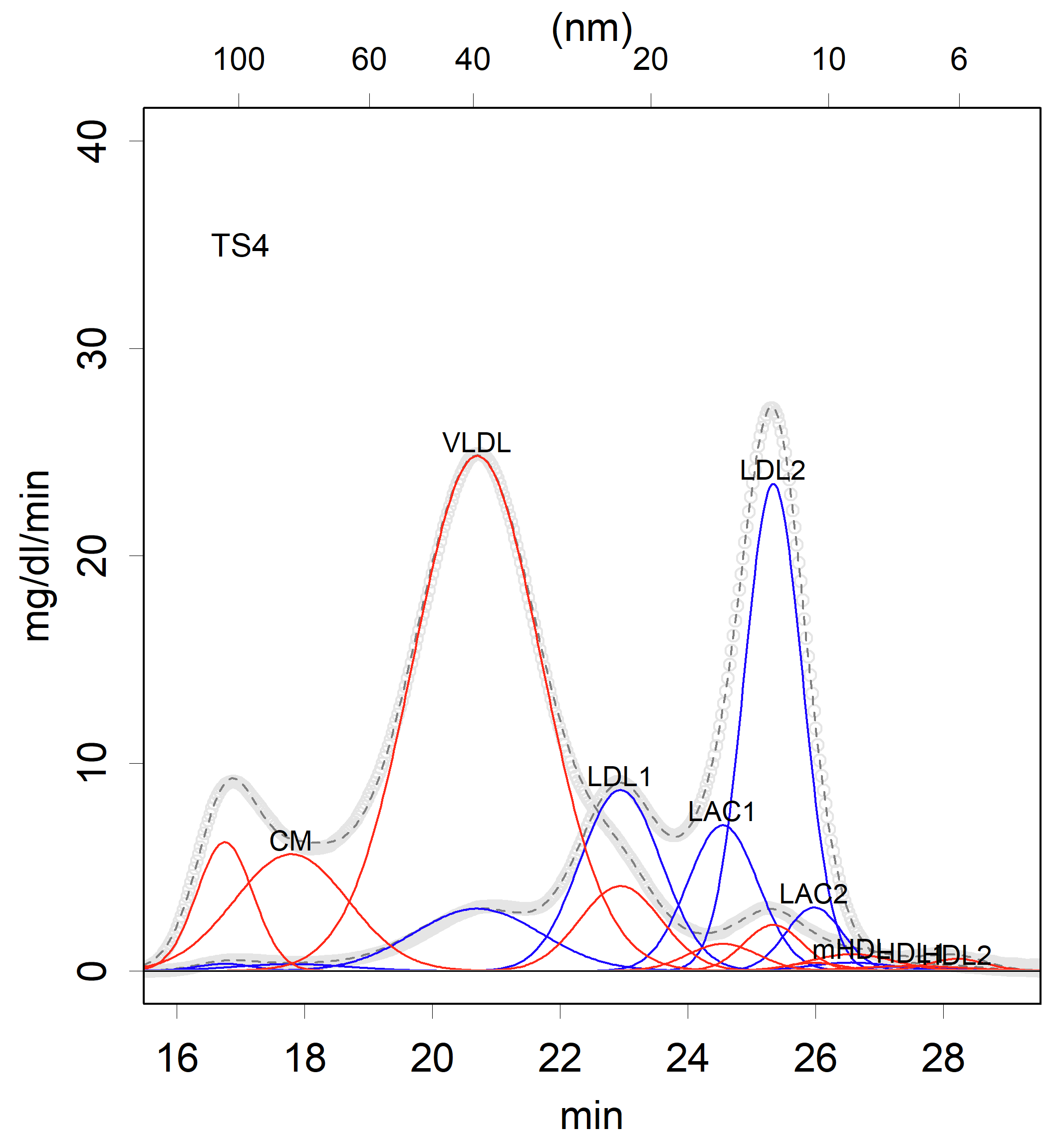

Supplement: S1 Fig — (ZIP) [file pone.0210950.s001.zip › S1_Fig/box/TS4.png]

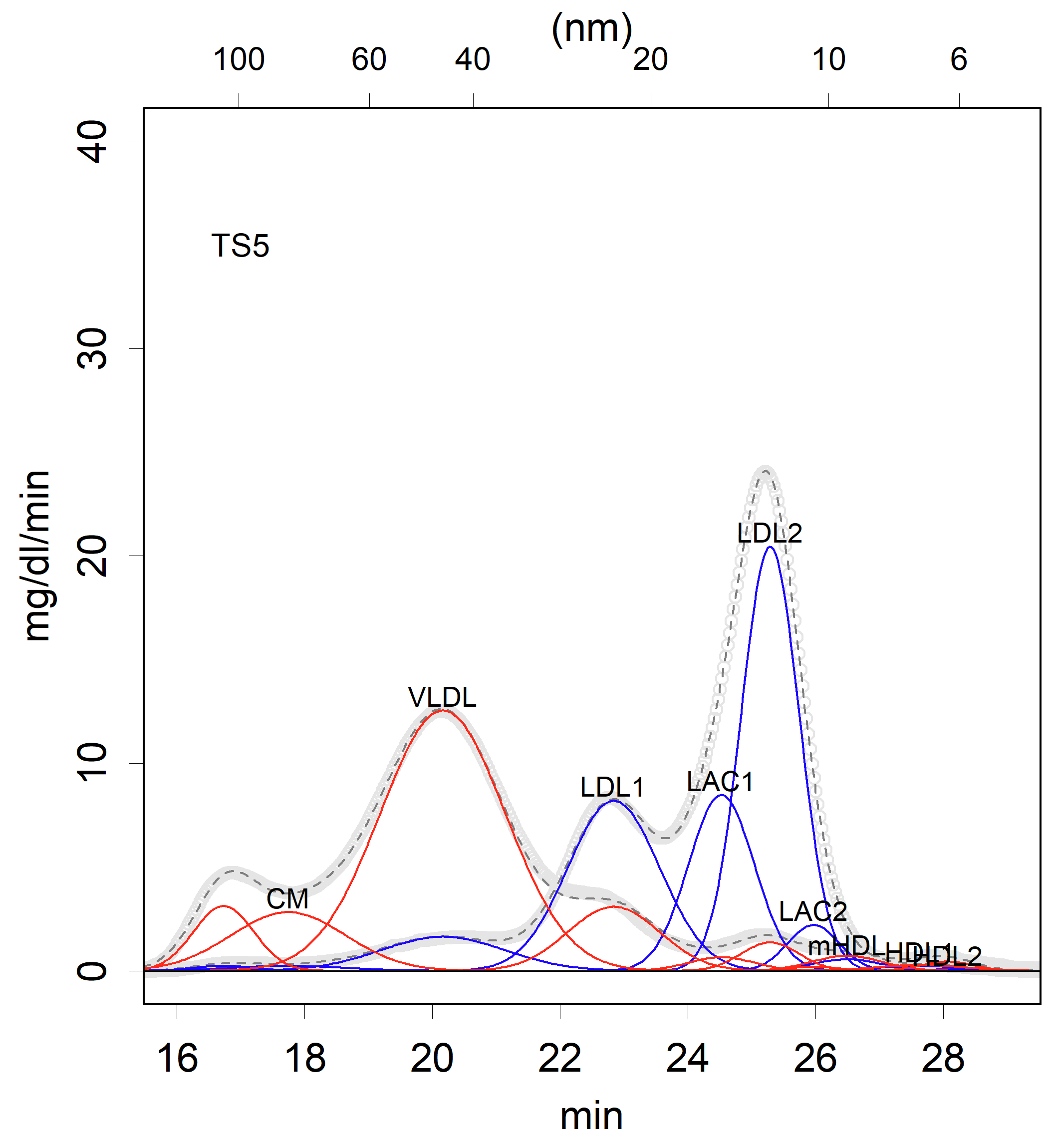

Supplement: S1 Fig — (ZIP) [file pone.0210950.s001.zip › S1_Fig/box/TS5.png]

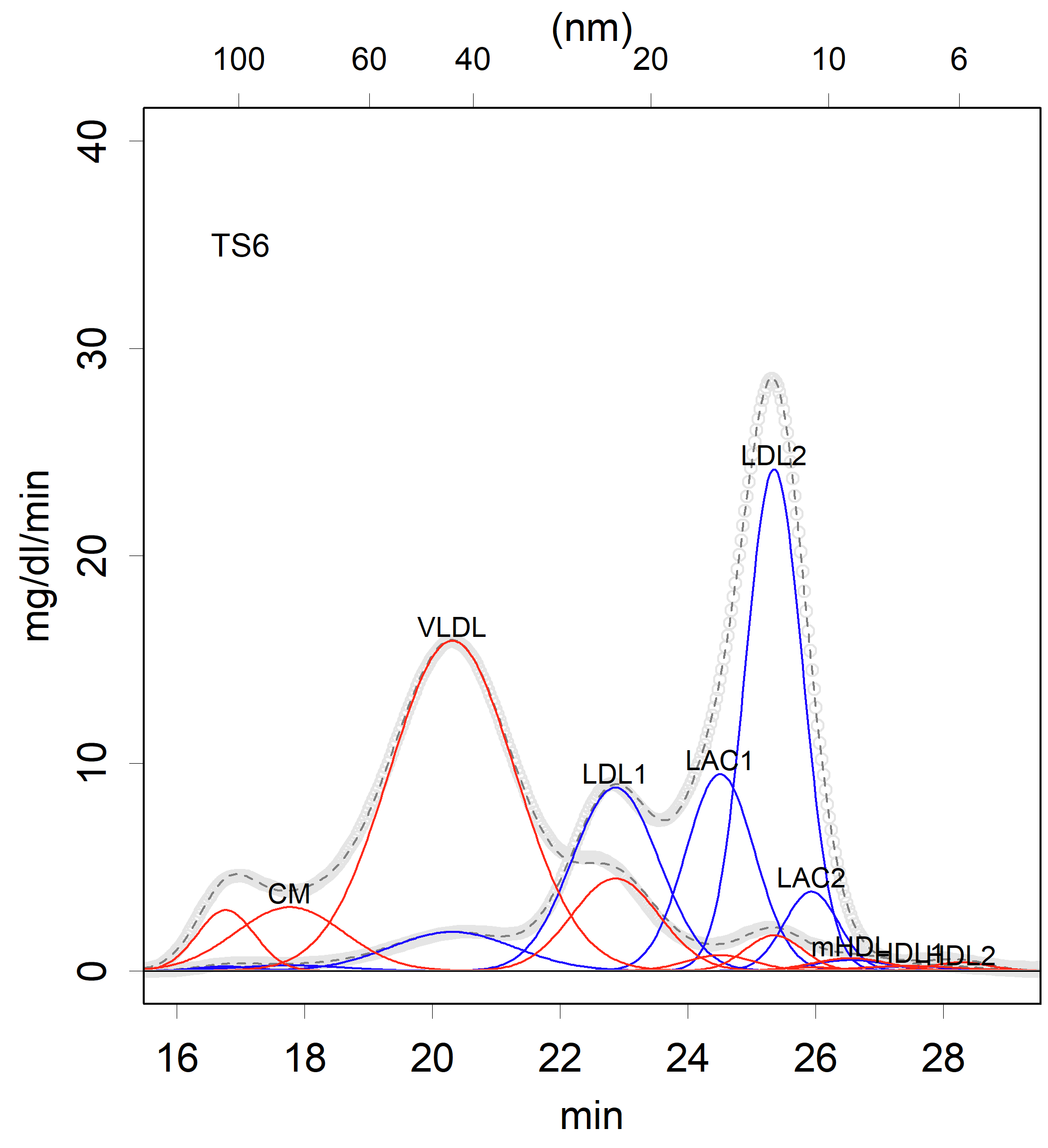

Supplement: S1 Fig — (ZIP) [file pone.0210950.s001.zip › S1_Fig/box/TS6.png]

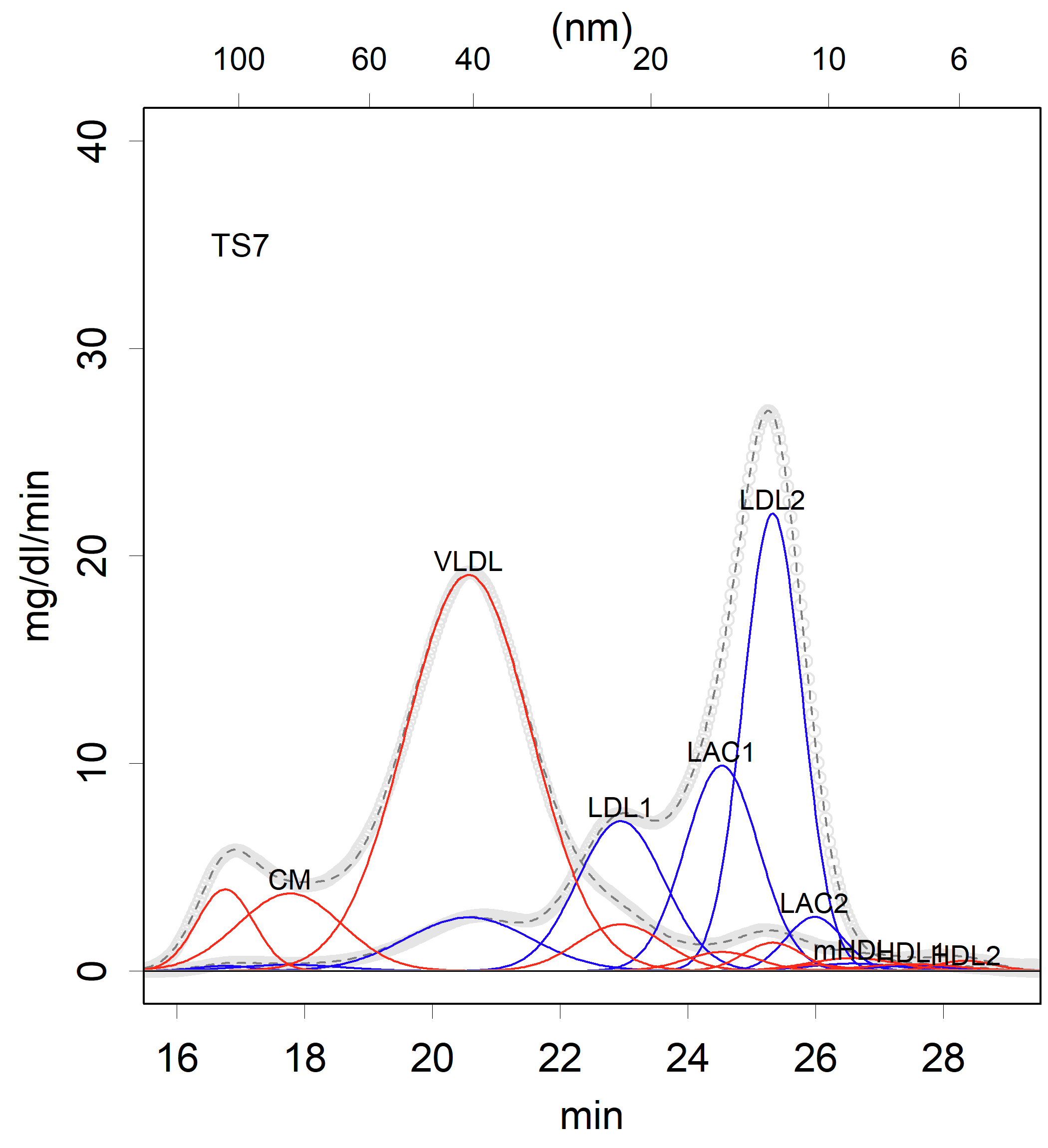

Supplement: S1 Fig — (ZIP) [file pone.0210950.s001.zip › S1_Fig/box/TS7.png]

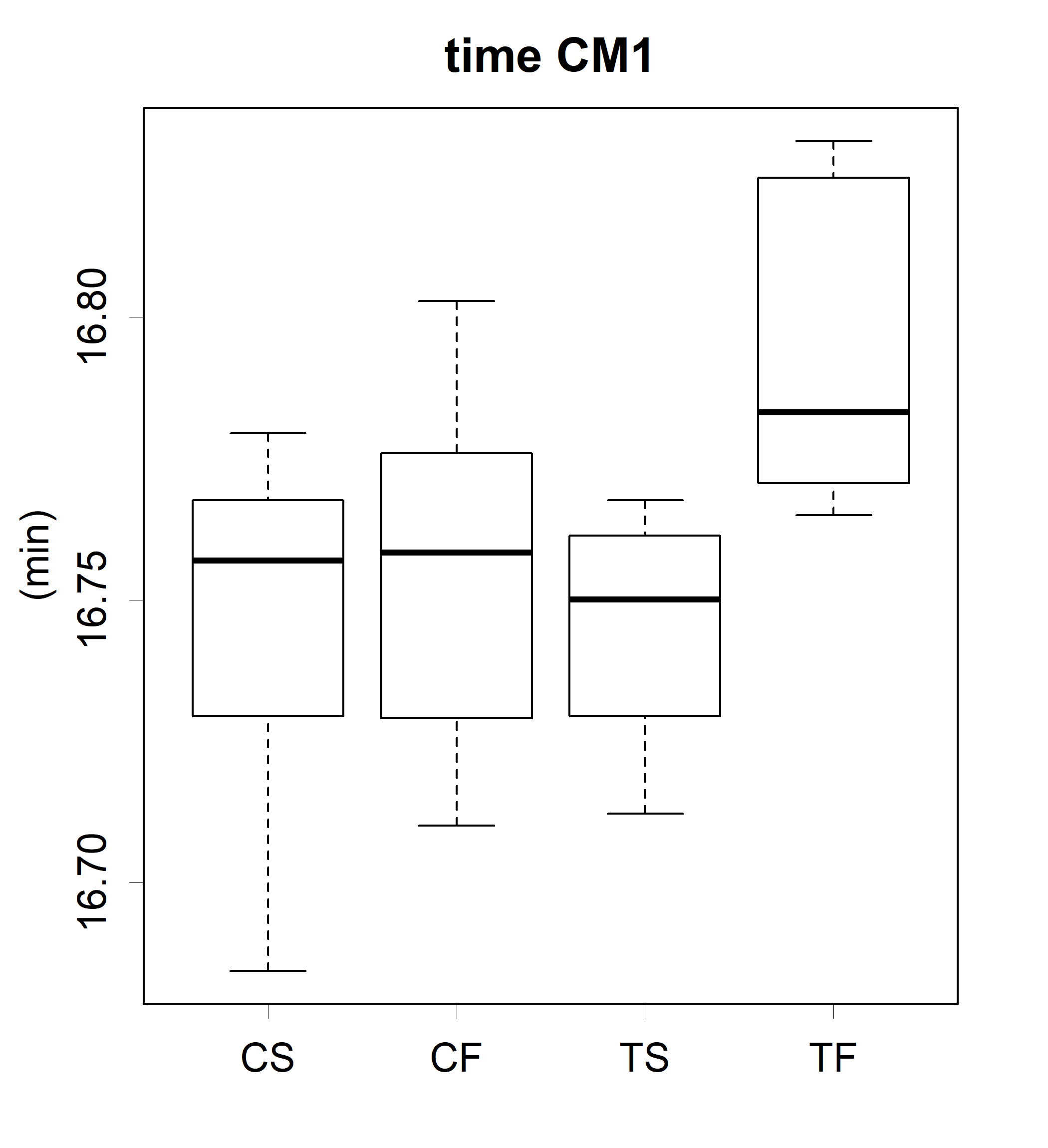

Supplement: S2 Fig — (ZIP) [file pone.0210950.s002.zip › S2_Fig/time/time_CM1.png]

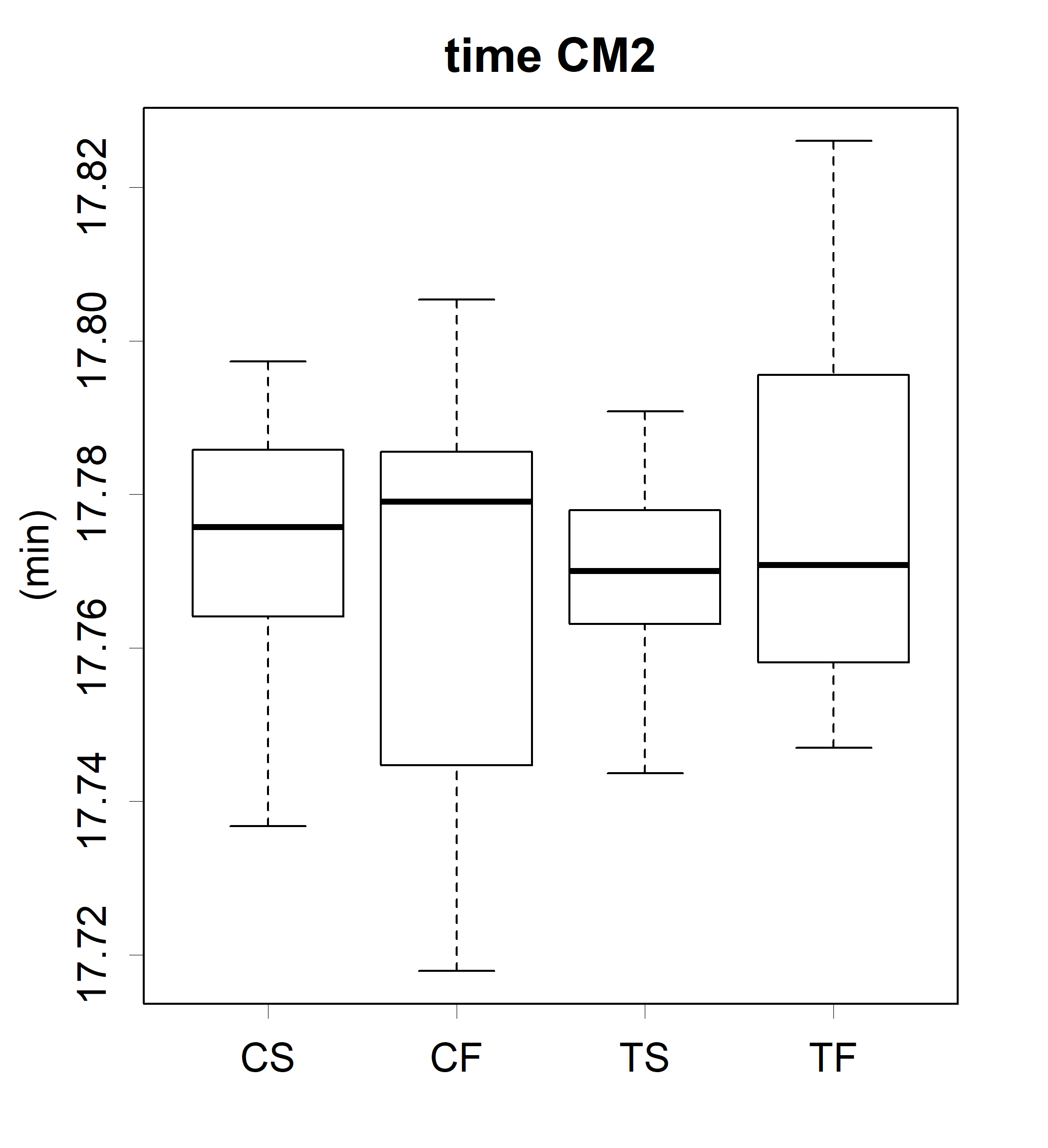

Supplement: S2 Fig — (ZIP) [file pone.0210950.s002.zip › S2_Fig/time/time_CM2.png]

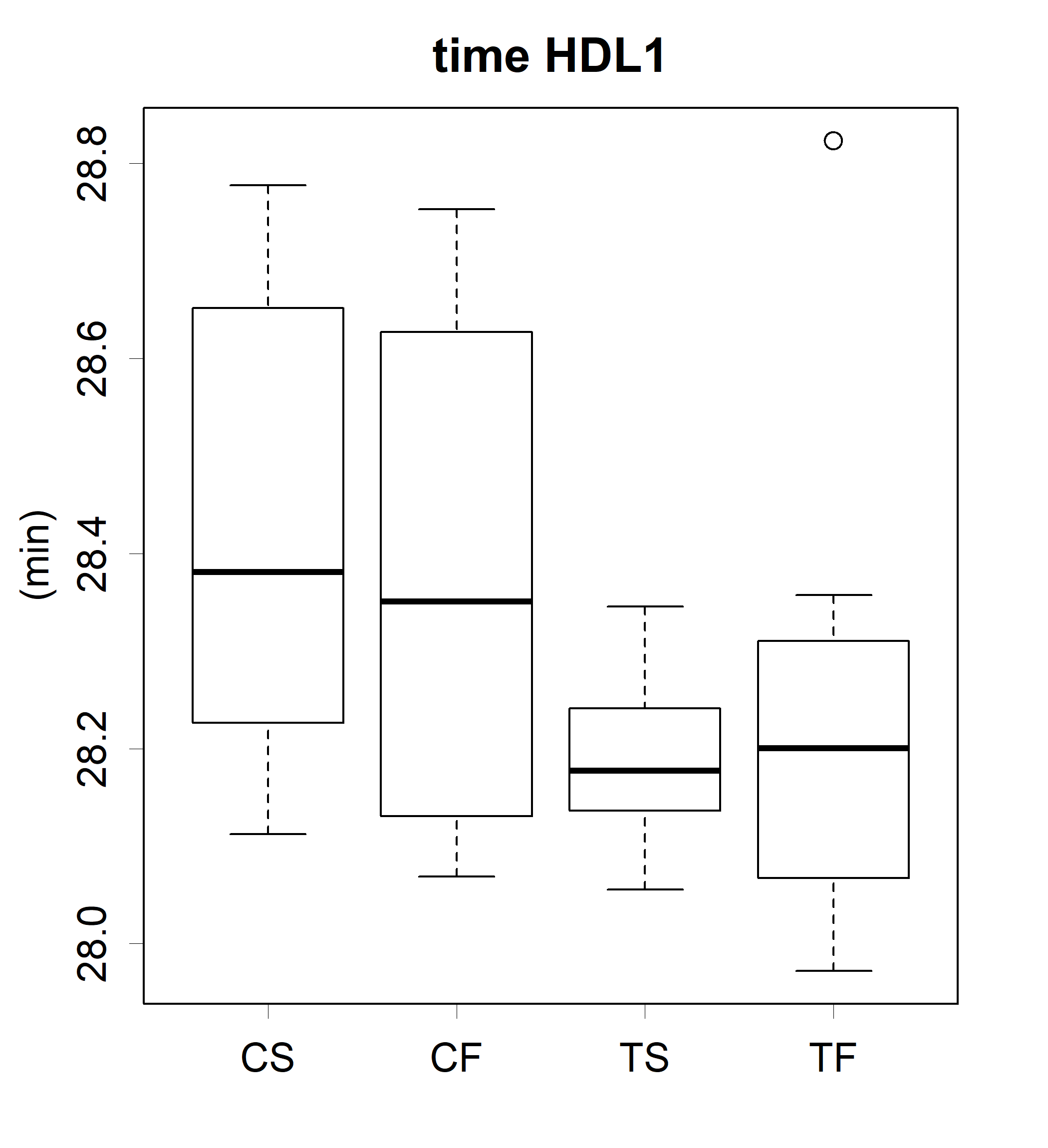

Supplement: S2 Fig — (ZIP) [file pone.0210950.s002.zip › S2_Fig/time/time_HDL1.png]

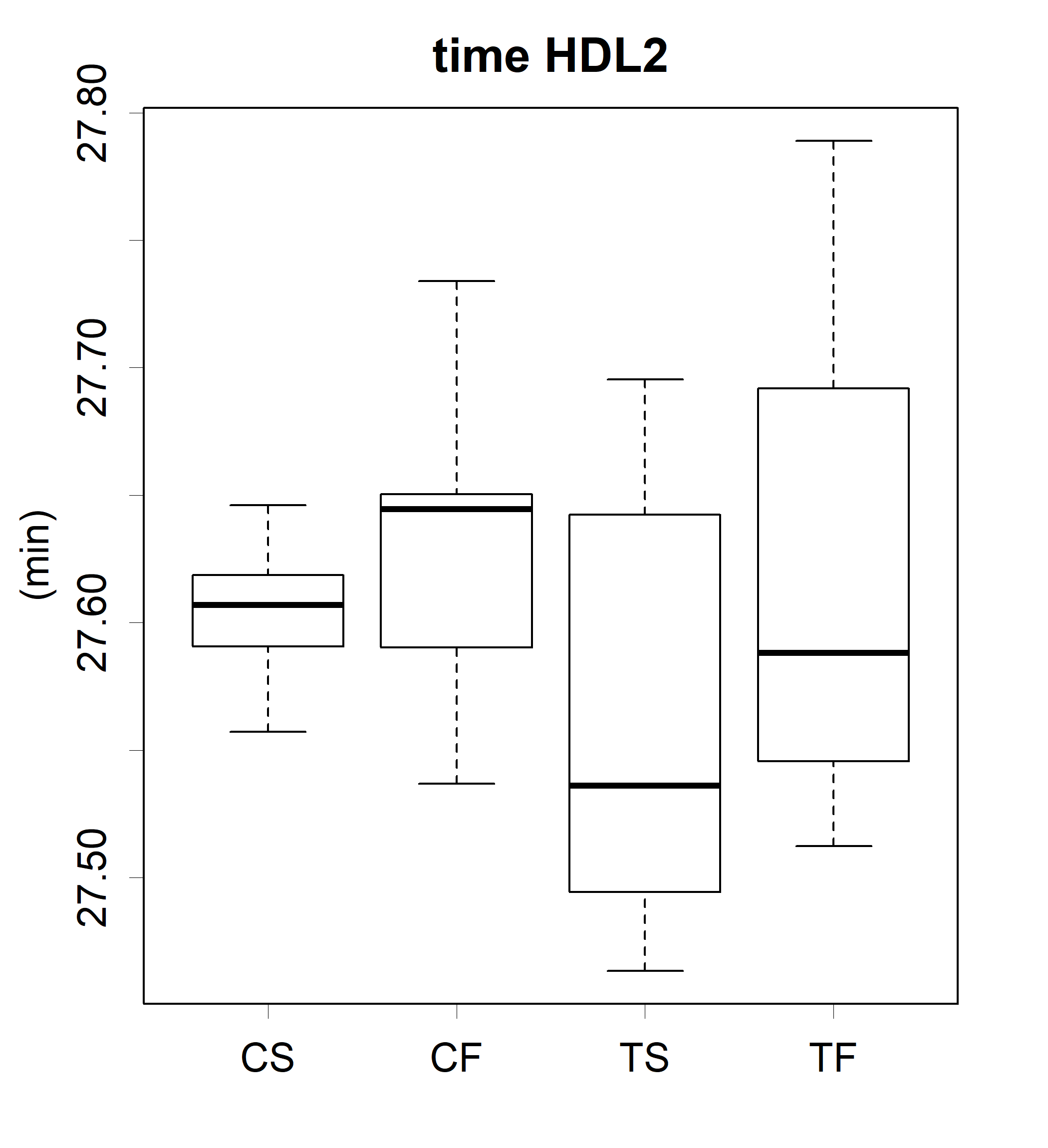

Supplement: S2 Fig — (ZIP) [file pone.0210950.s002.zip › S2_Fig/time/time_HDL2.png]

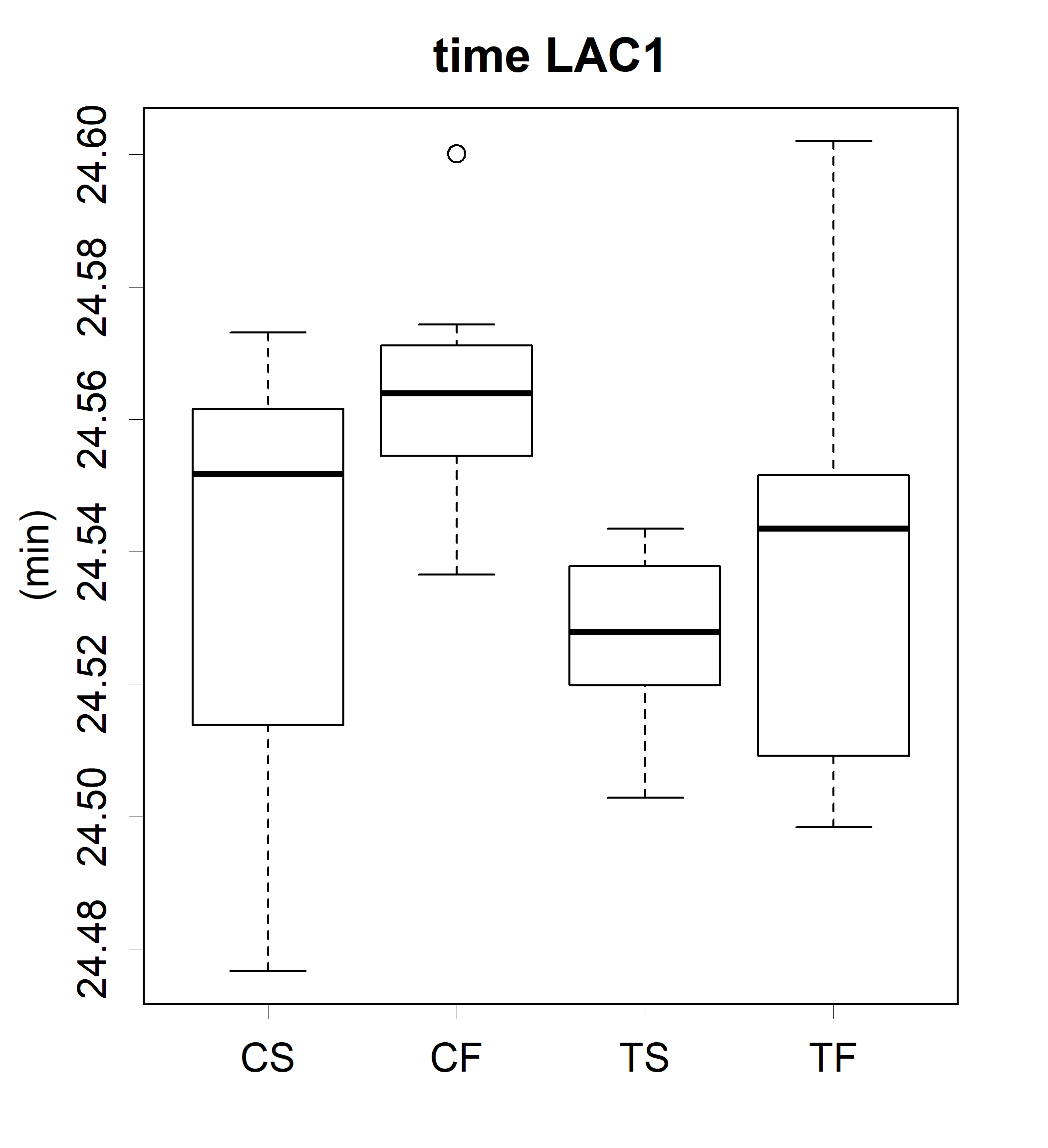

Supplement: S2 Fig — (ZIP) [file pone.0210950.s002.zip › S2_Fig/time/time_LAC1.png]

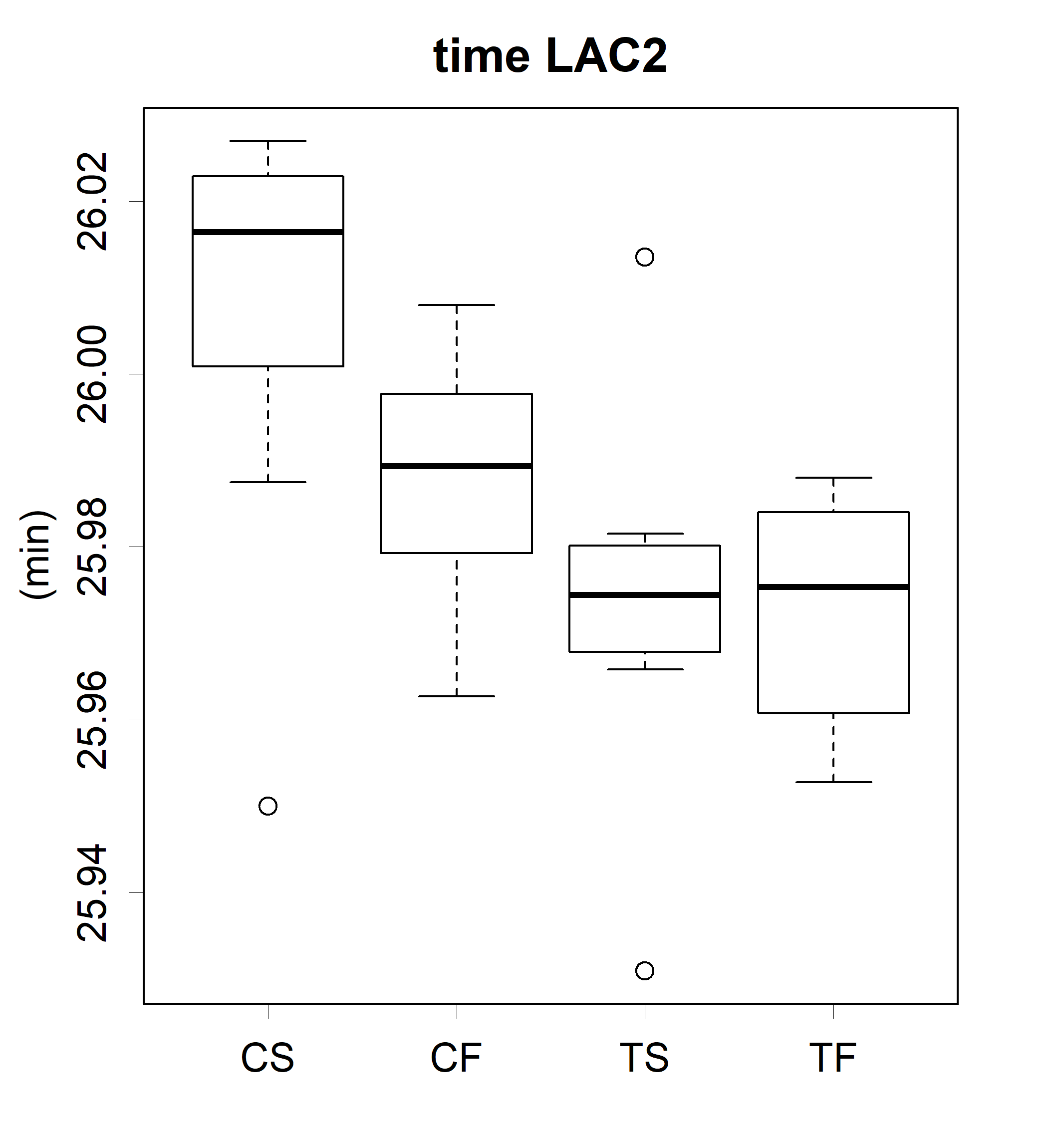

Supplement: S2 Fig — (ZIP) [file pone.0210950.s002.zip › S2_Fig/time/time_LAC2.png]

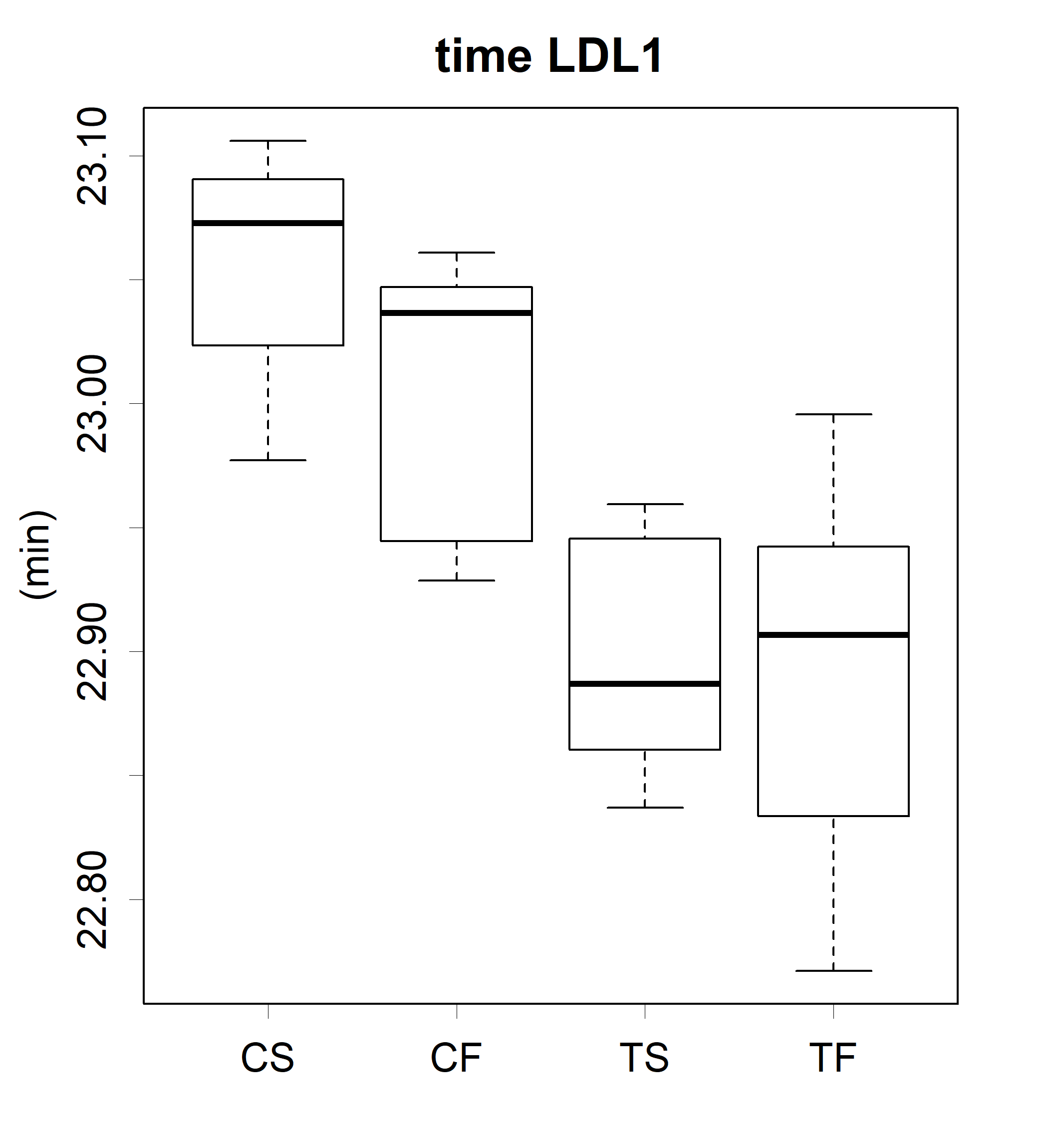

Supplement: S2 Fig — (ZIP) [file pone.0210950.s002.zip › S2_Fig/time/time_LDL1.png]

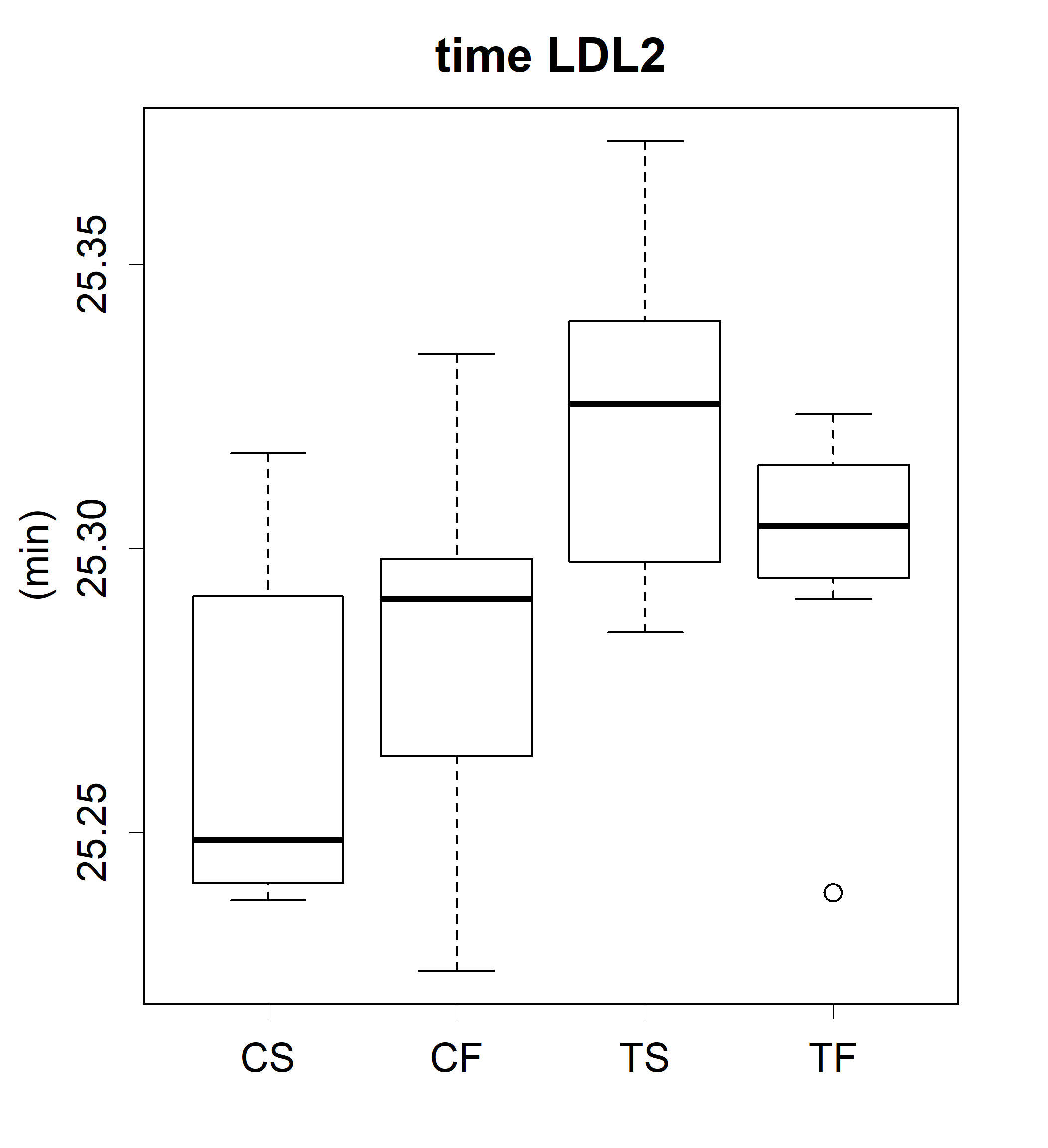

Supplement: S2 Fig — (ZIP) [file pone.0210950.s002.zip › S2_Fig/time/time_LDL2.png]

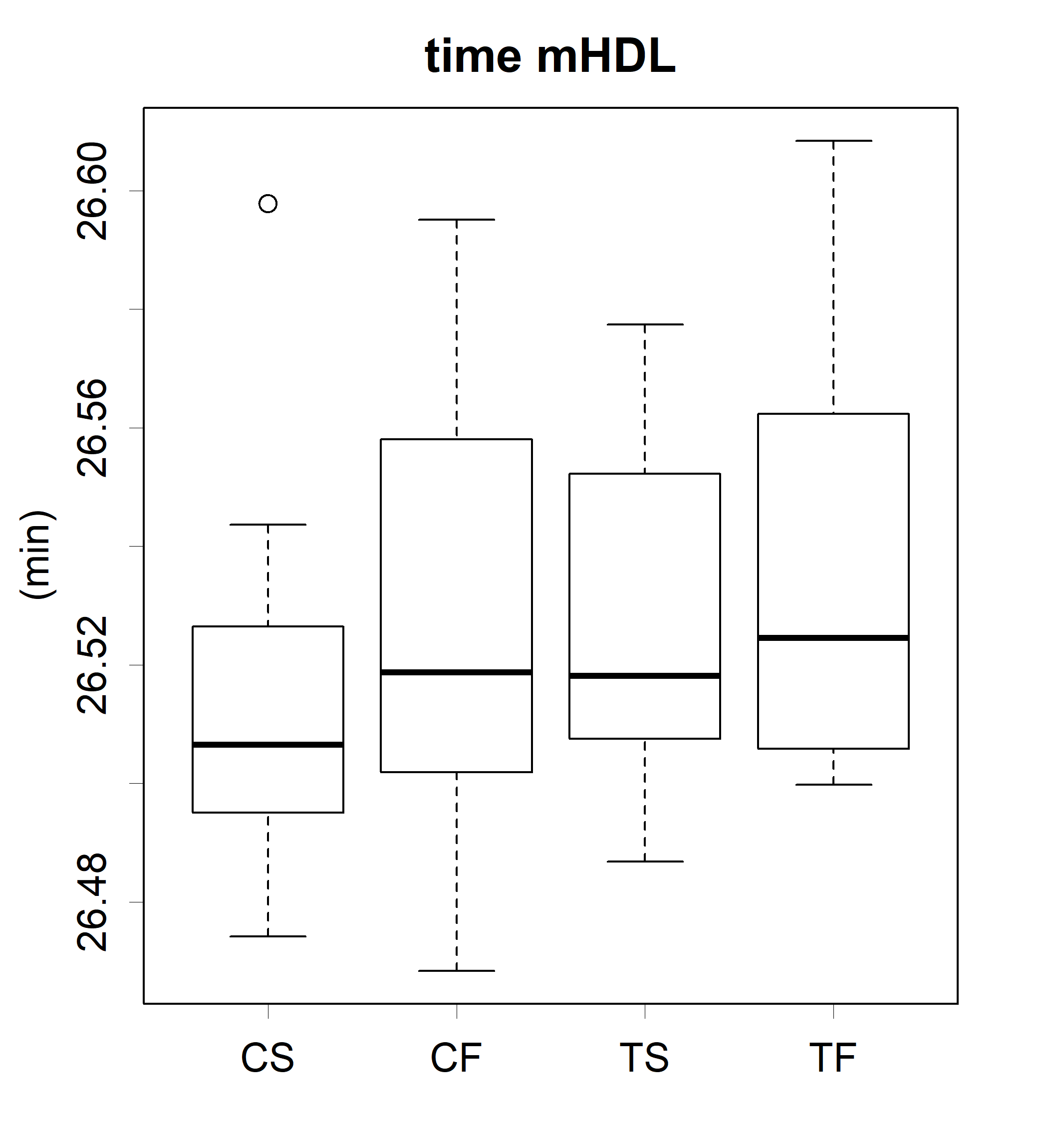

Supplement: S2 Fig — (ZIP) [file pone.0210950.s002.zip › S2_Fig/time/time_mHDL.png]

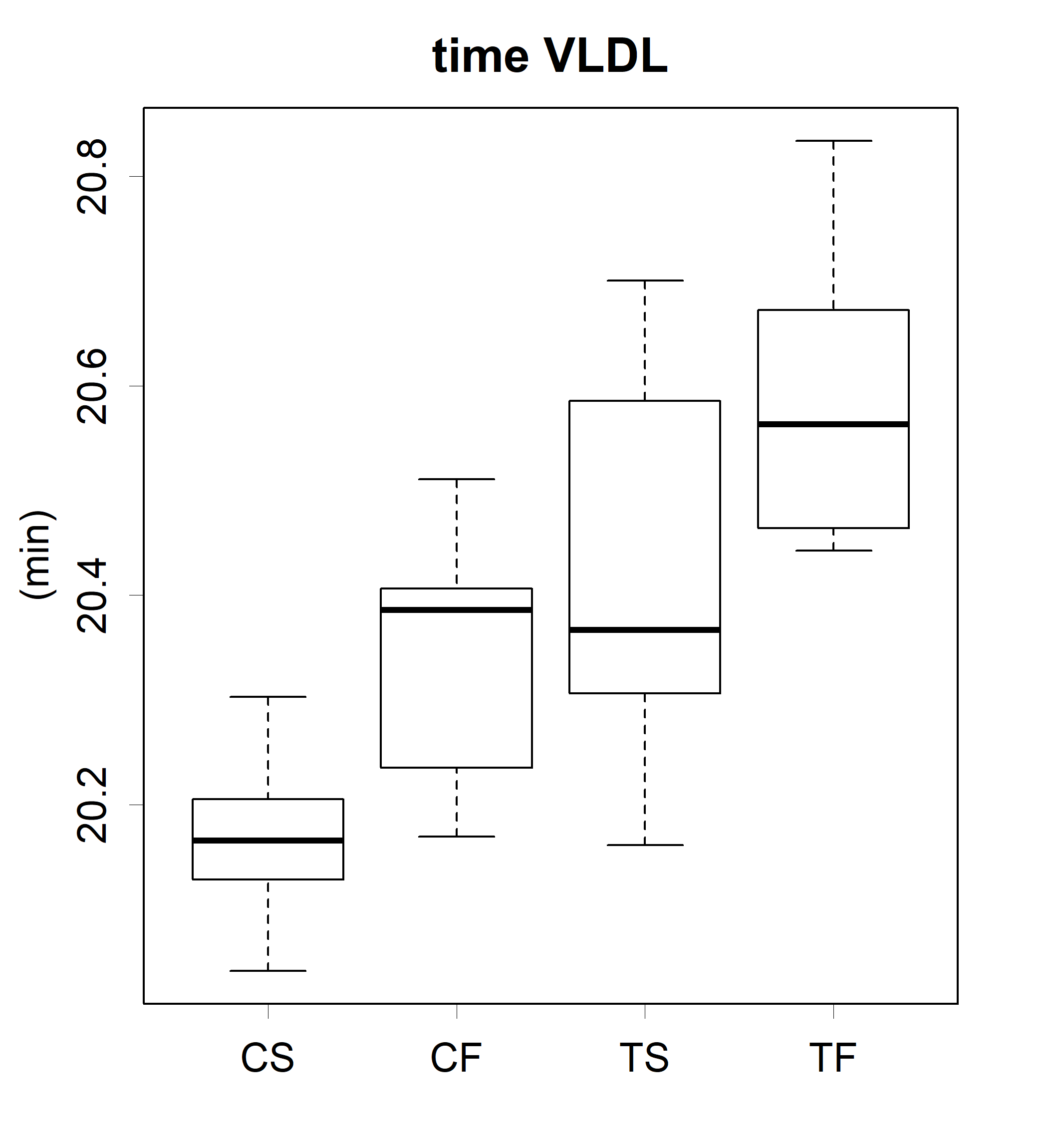

Supplement: S2 Fig — (ZIP) [file pone.0210950.s002.zip › S2_Fig/time/time_VLDL.png]

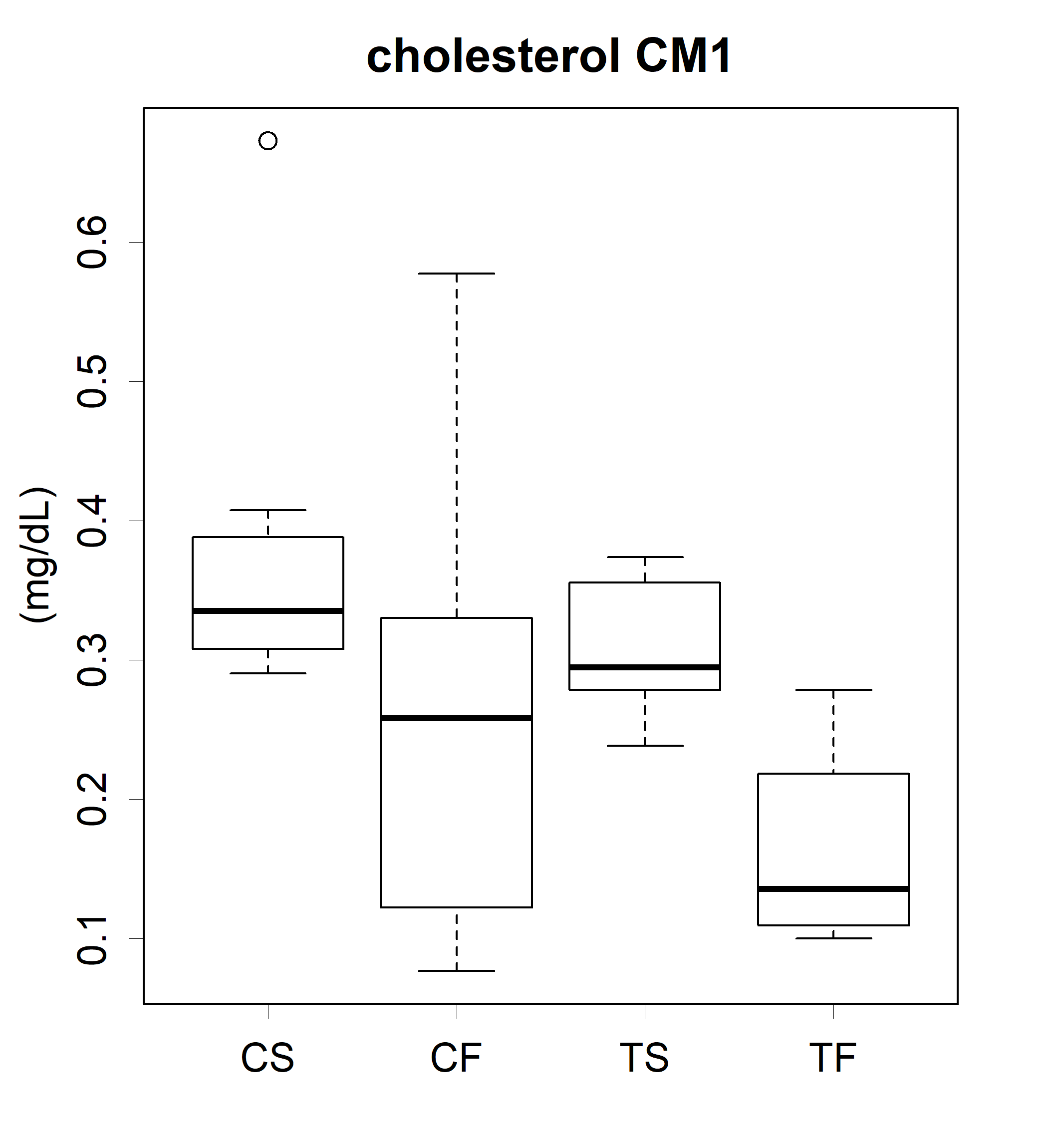

Supplement: S3 Fig — (ZIP) [file pone.0210950.s003.zip › S3_Fig/Ch/Ch_CM1.png]

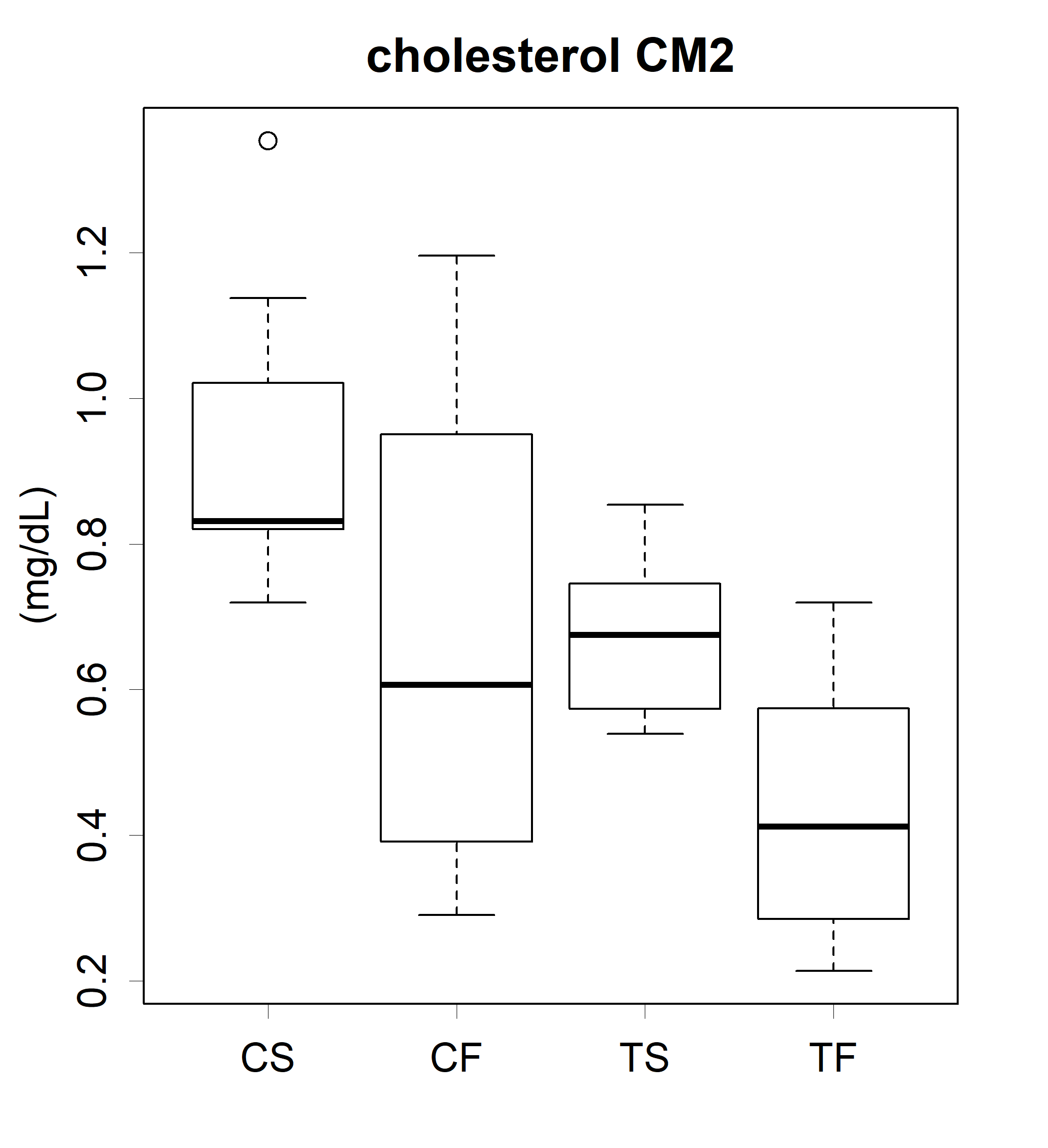

Supplement: S3 Fig — (ZIP) [file pone.0210950.s003.zip › S3_Fig/Ch/Ch_CM2.png]

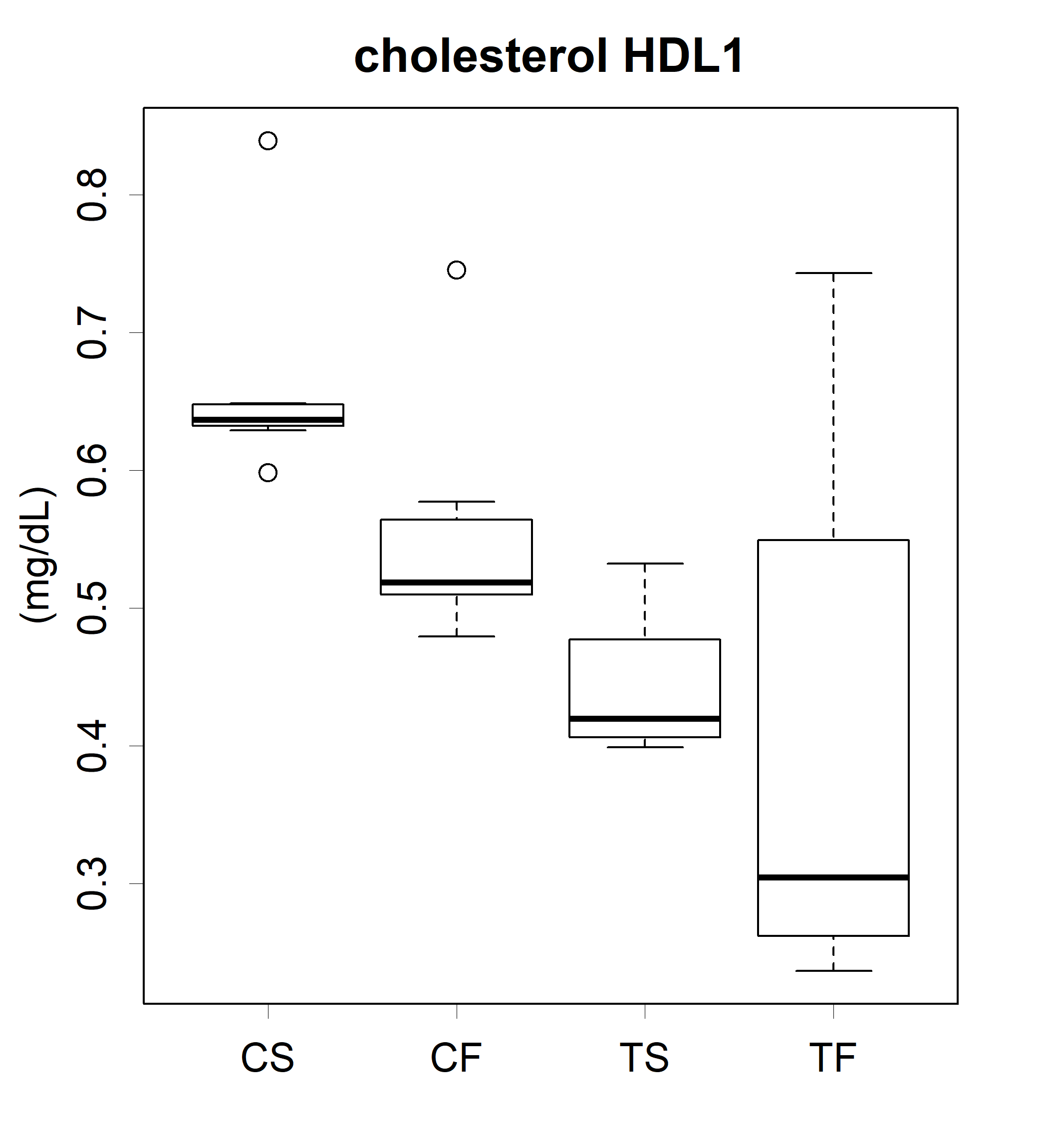

Supplement: S3 Fig — (ZIP) [file pone.0210950.s003.zip › S3_Fig/Ch/Ch_HDL1.png]

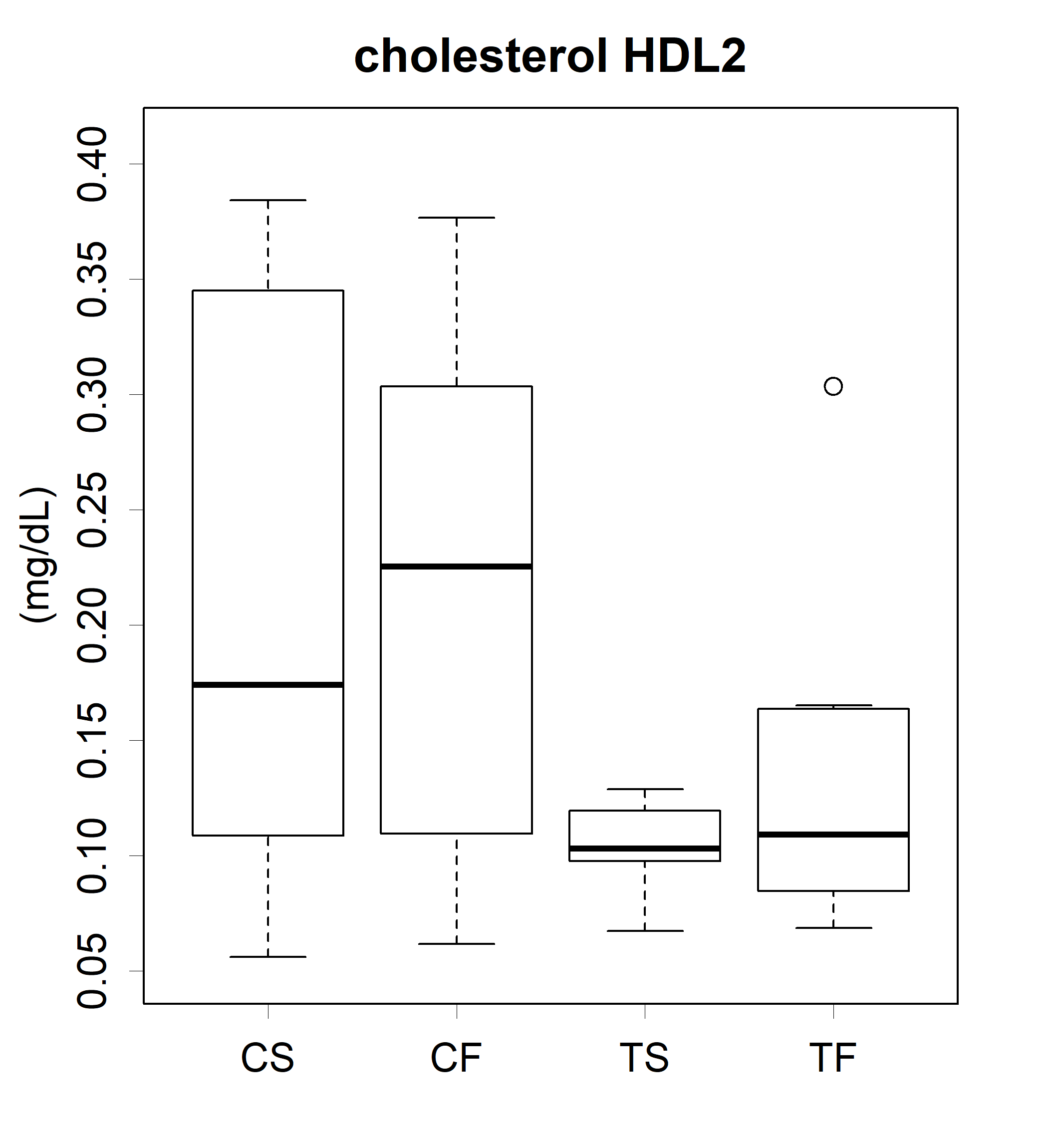

Supplement: S3 Fig — (ZIP) [file pone.0210950.s003.zip › S3_Fig/Ch/Ch_HDL2.png]

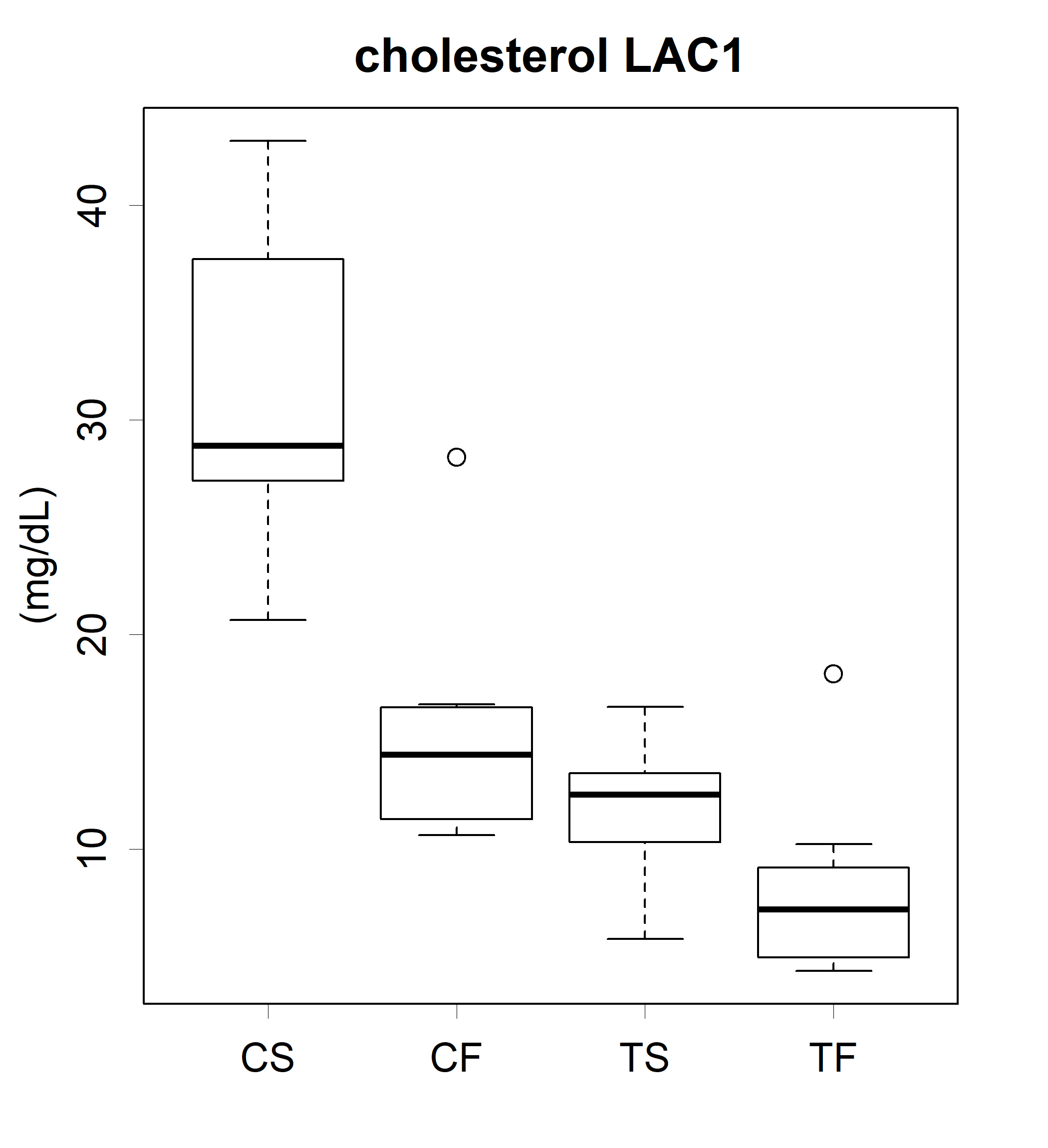

Supplement: S3 Fig — (ZIP) [file pone.0210950.s003.zip › S3_Fig/Ch/Ch_LAC1.png]

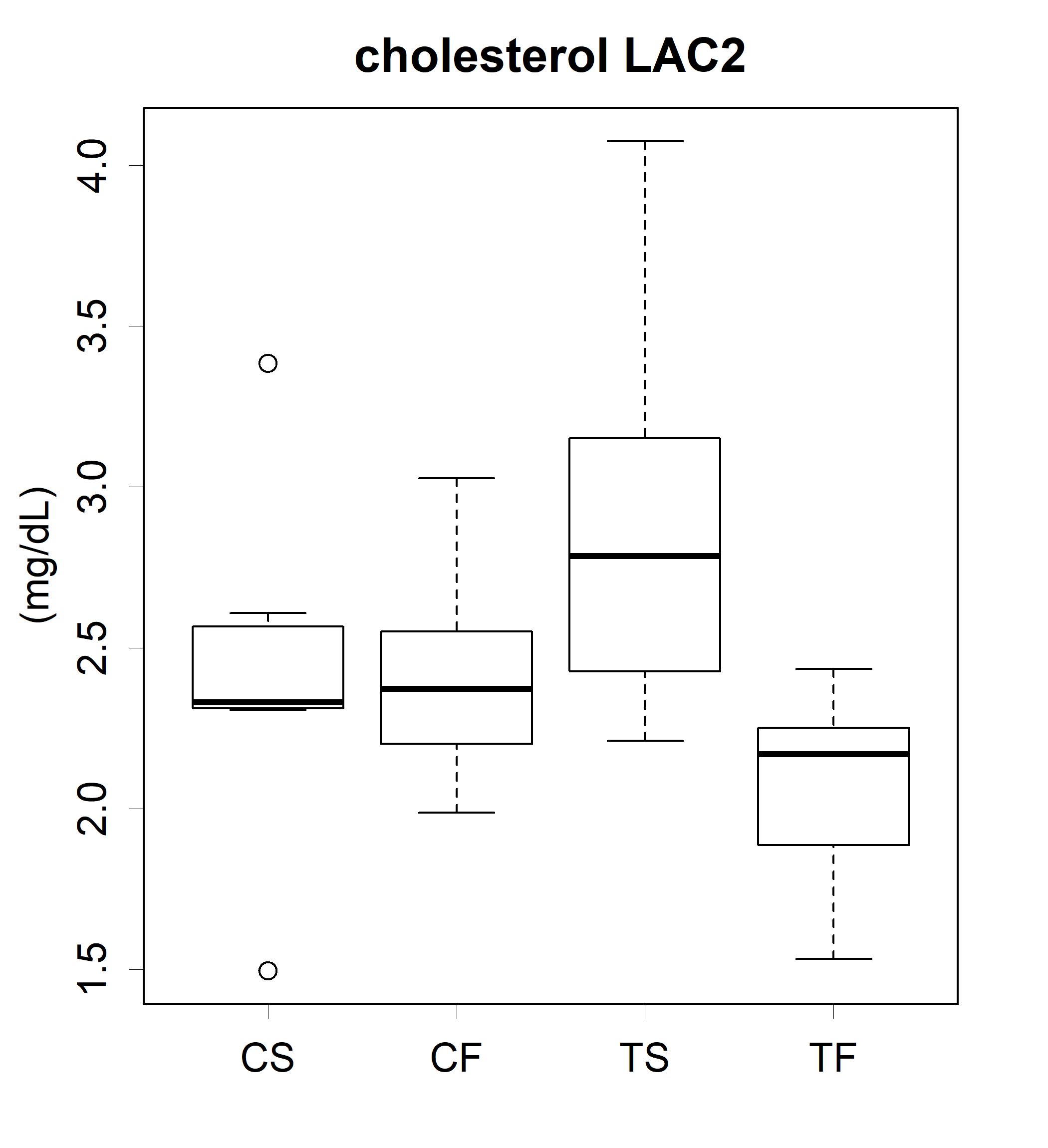

Supplement: S3 Fig — (ZIP) [file pone.0210950.s003.zip › S3_Fig/Ch/Ch_LAC2.png]

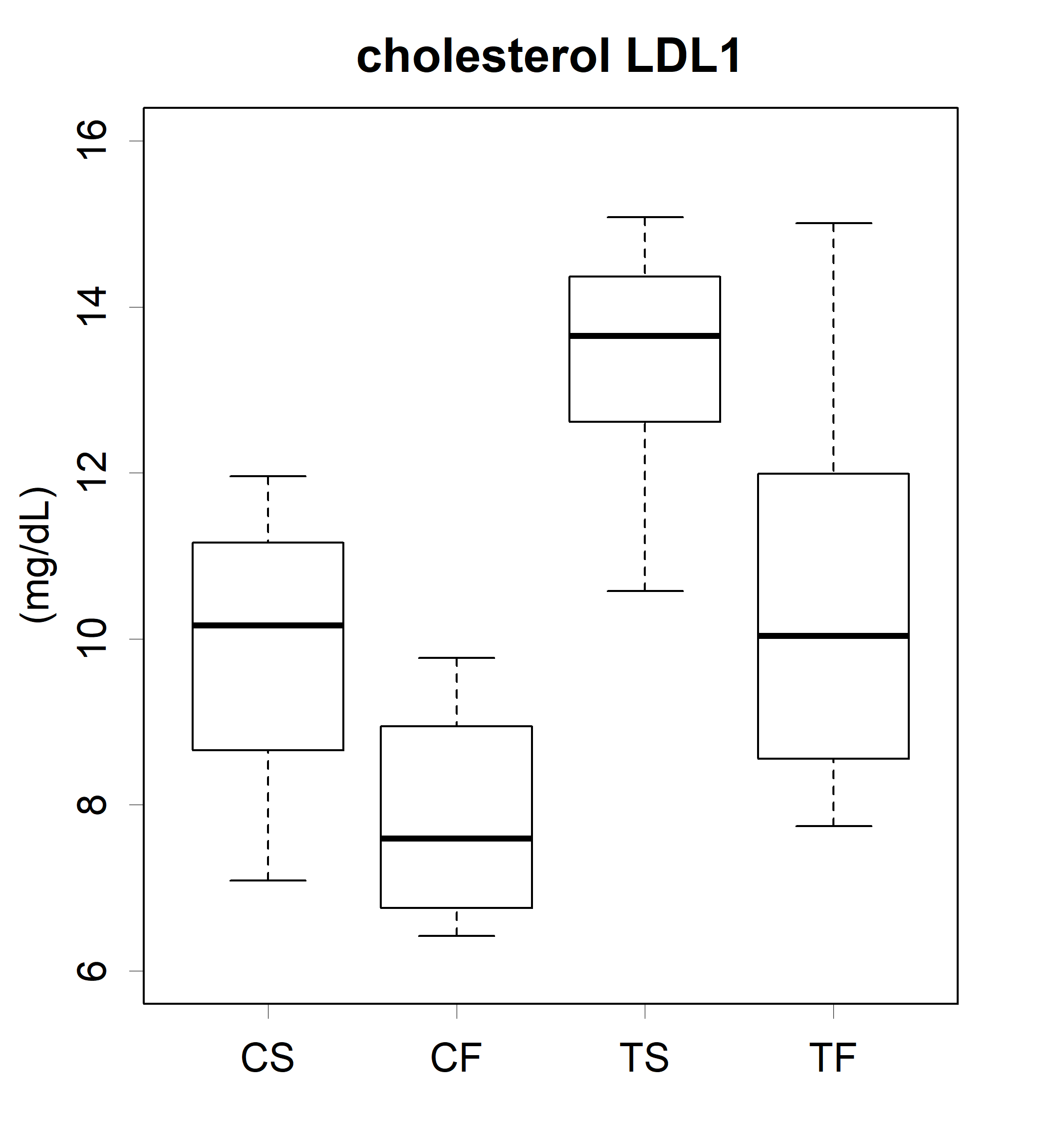

Supplement: S3 Fig — (ZIP) [file pone.0210950.s003.zip › S3_Fig/Ch/Ch_LDL1.png]

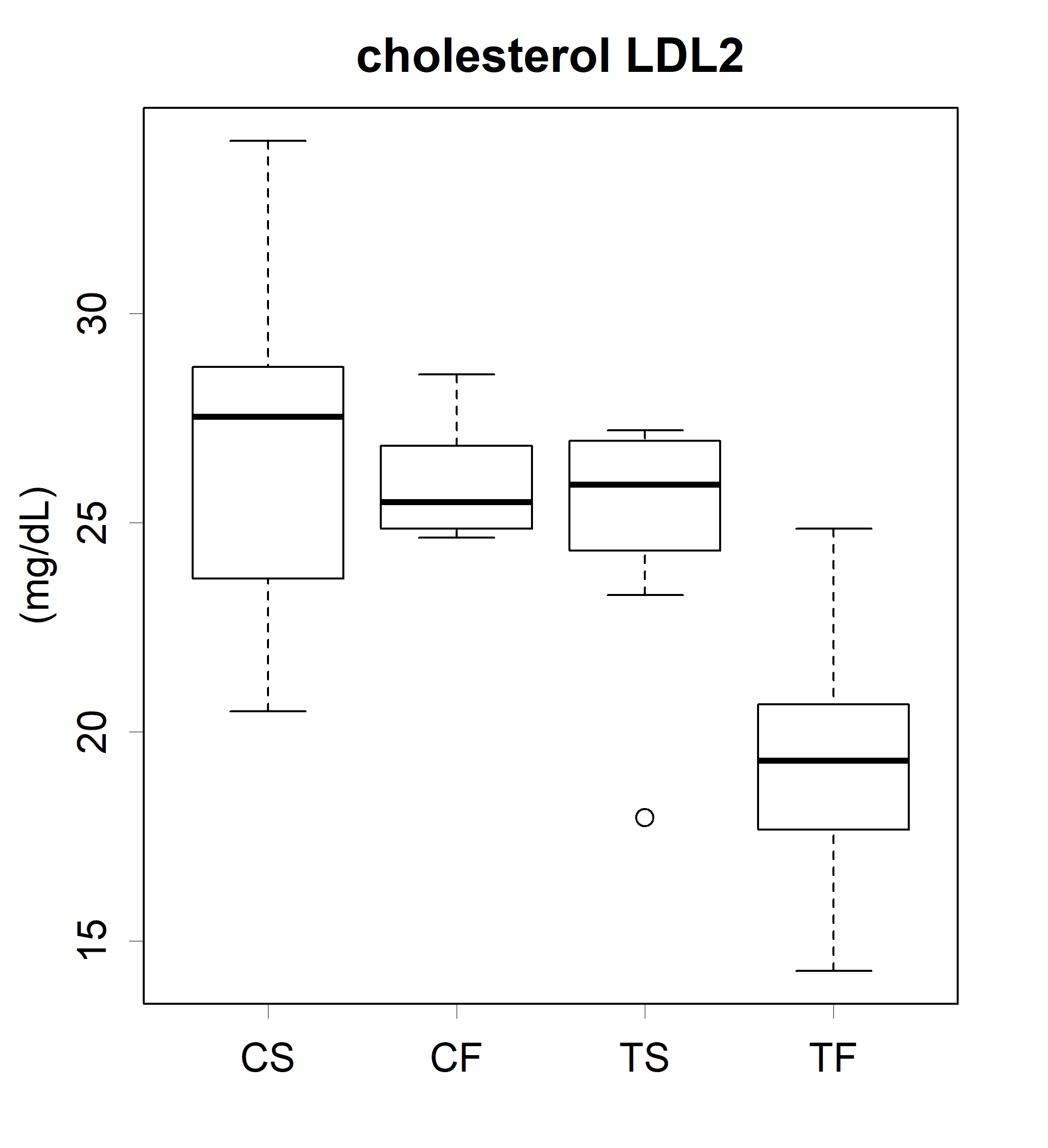

Supplement: S3 Fig — (ZIP) [file pone.0210950.s003.zip › S3_Fig/Ch/Ch_LDL2.png]

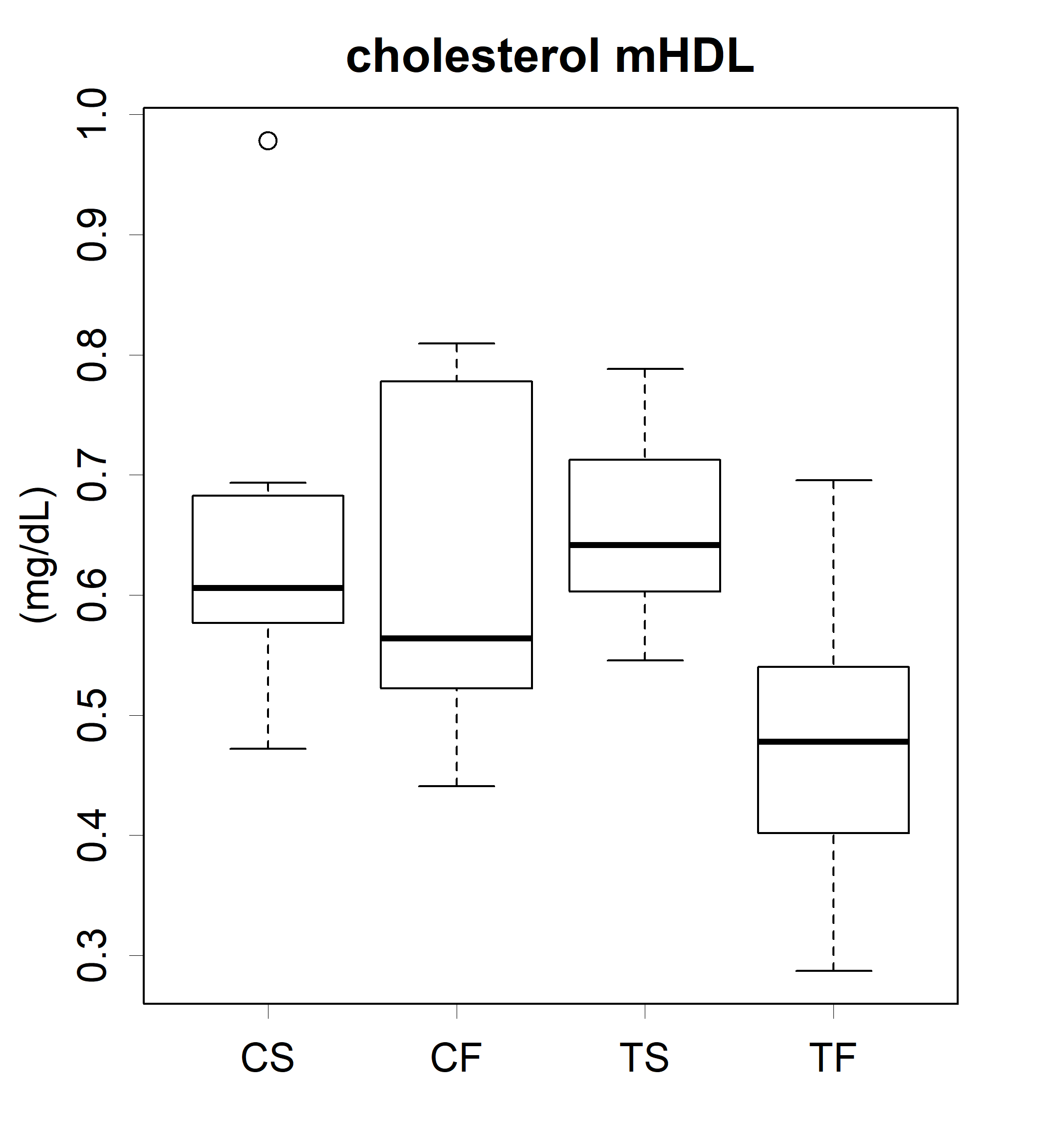

Supplement: S3 Fig — (ZIP) [file pone.0210950.s003.zip › S3_Fig/Ch/Ch_mHDL.png]

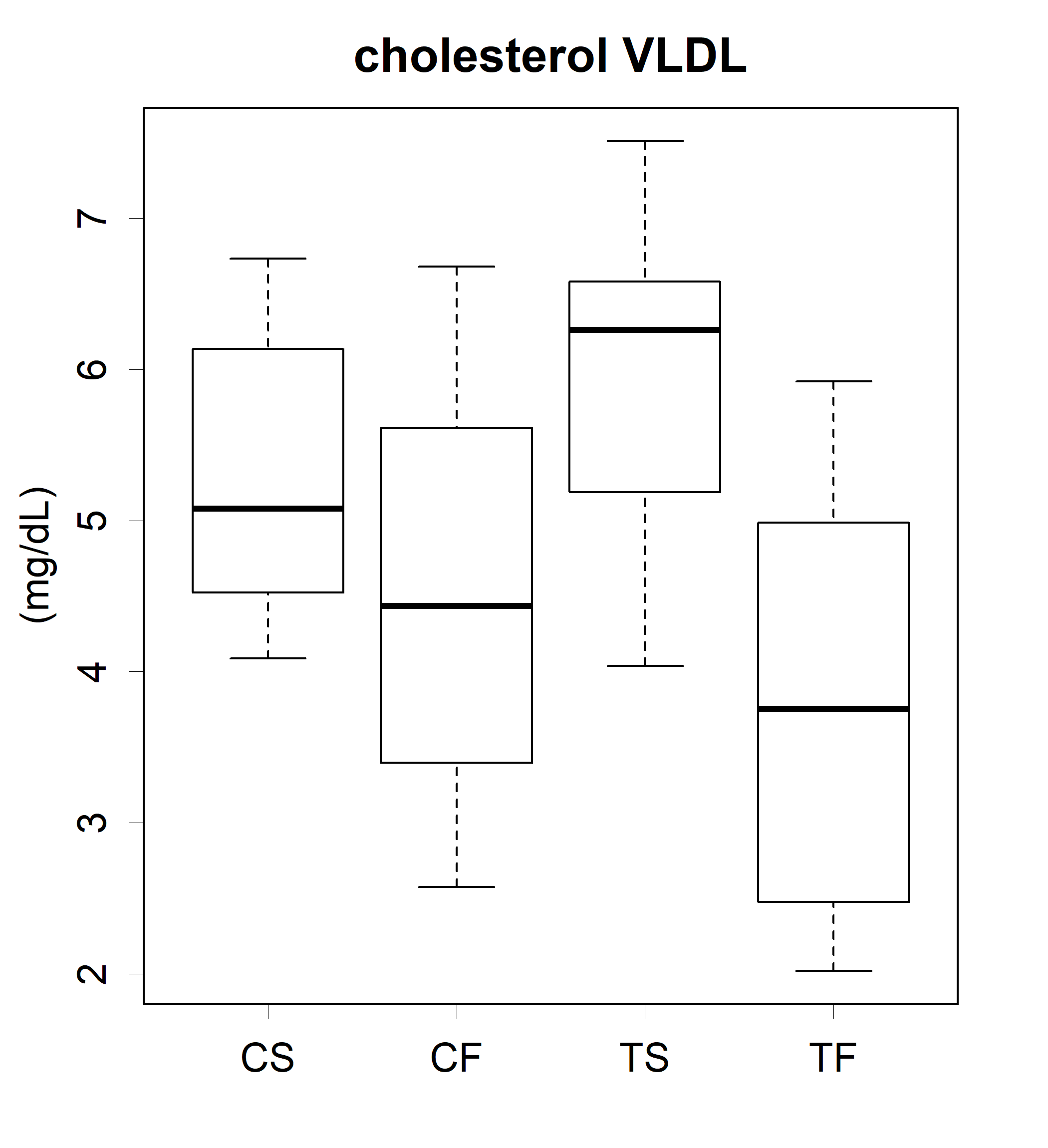

Supplement: S3 Fig — (ZIP) [file pone.0210950.s003.zip › S3_Fig/Ch/Ch_VLDL.png]

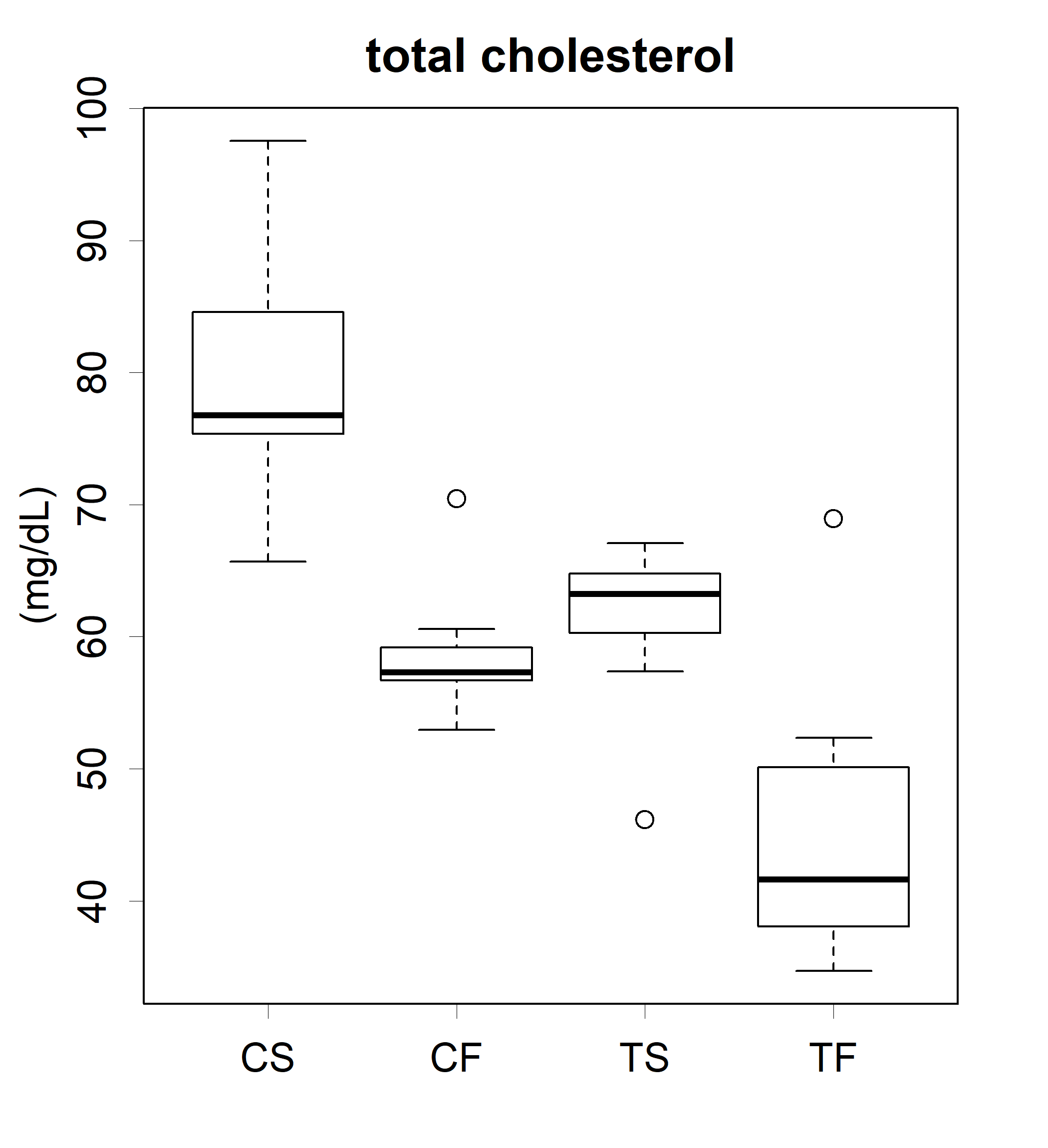

Supplement: S3 Fig — (ZIP) [file pone.0210950.s003.zip › S3_Fig/Ch/cholesterol.png]

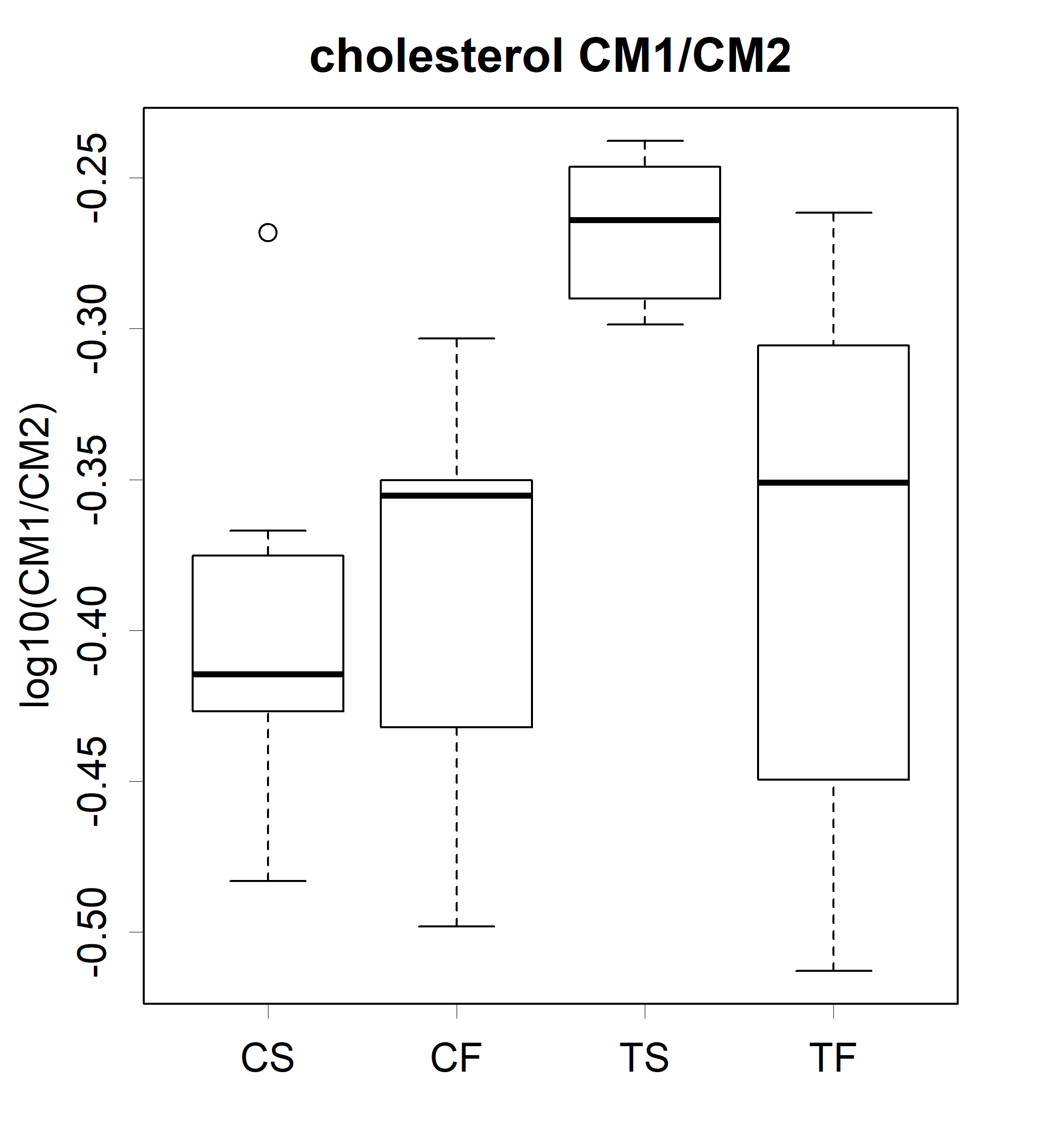

Supplement: S3 Fig — (ZIP) [file pone.0210950.s003.zip › S3_Fig/Ch/CM1_CM2.png]

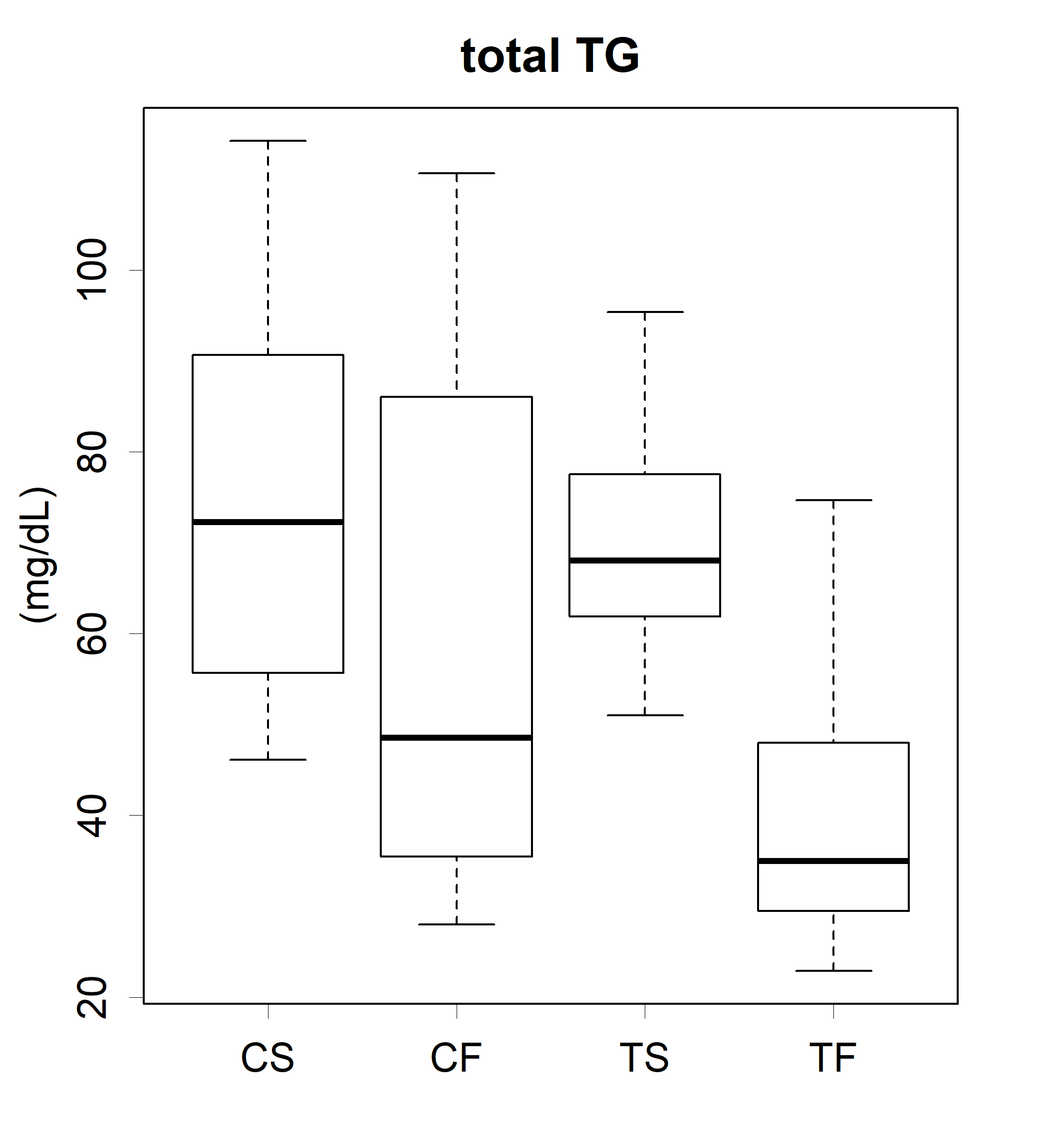

Supplement: S4 Fig — (ZIP) [file pone.0210950.s004.zip › S4_Fig/TG/TG.png]

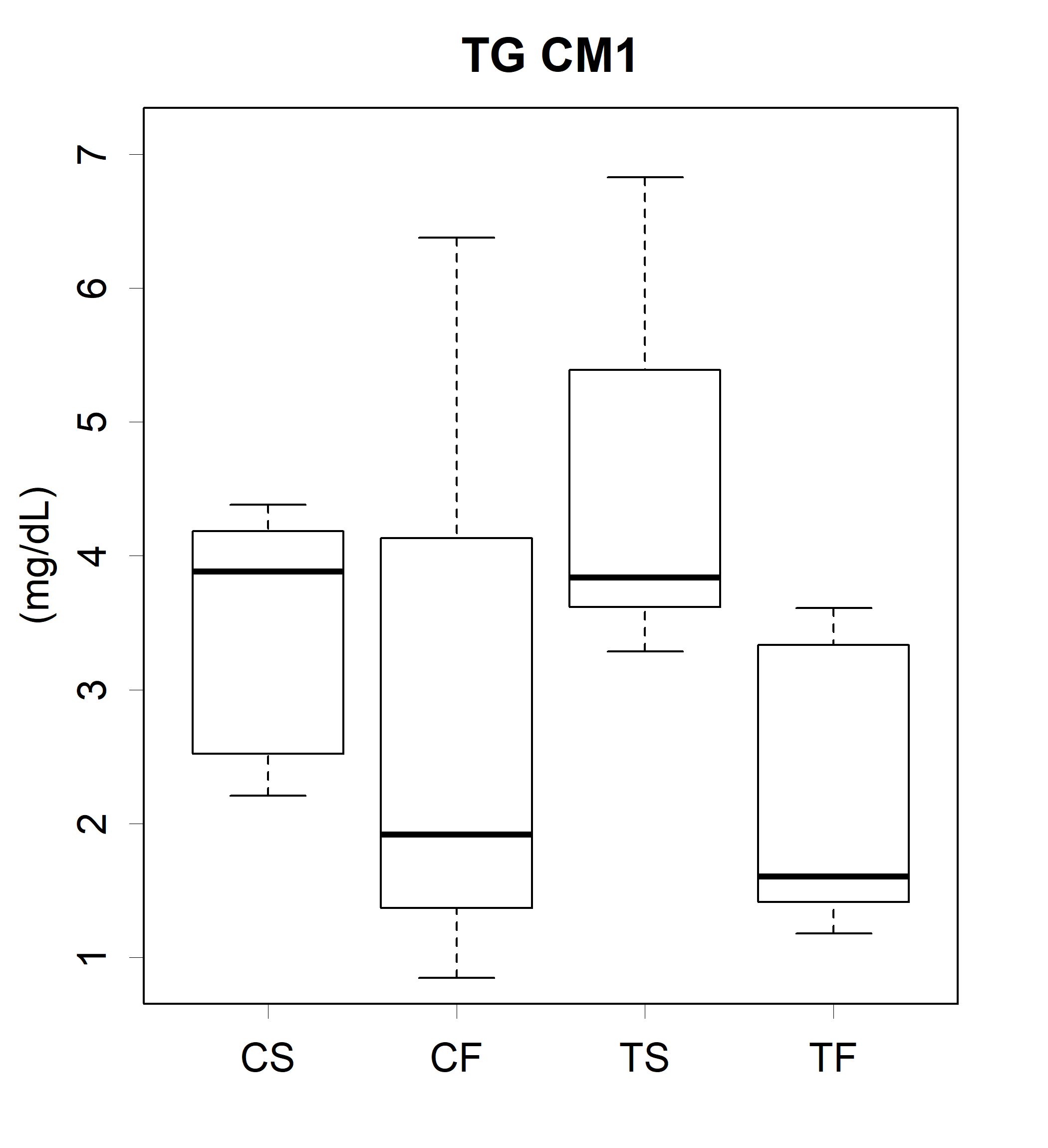

Supplement: S4 Fig — (ZIP) [file pone.0210950.s004.zip › S4_Fig/TG/TG_CM1.png]

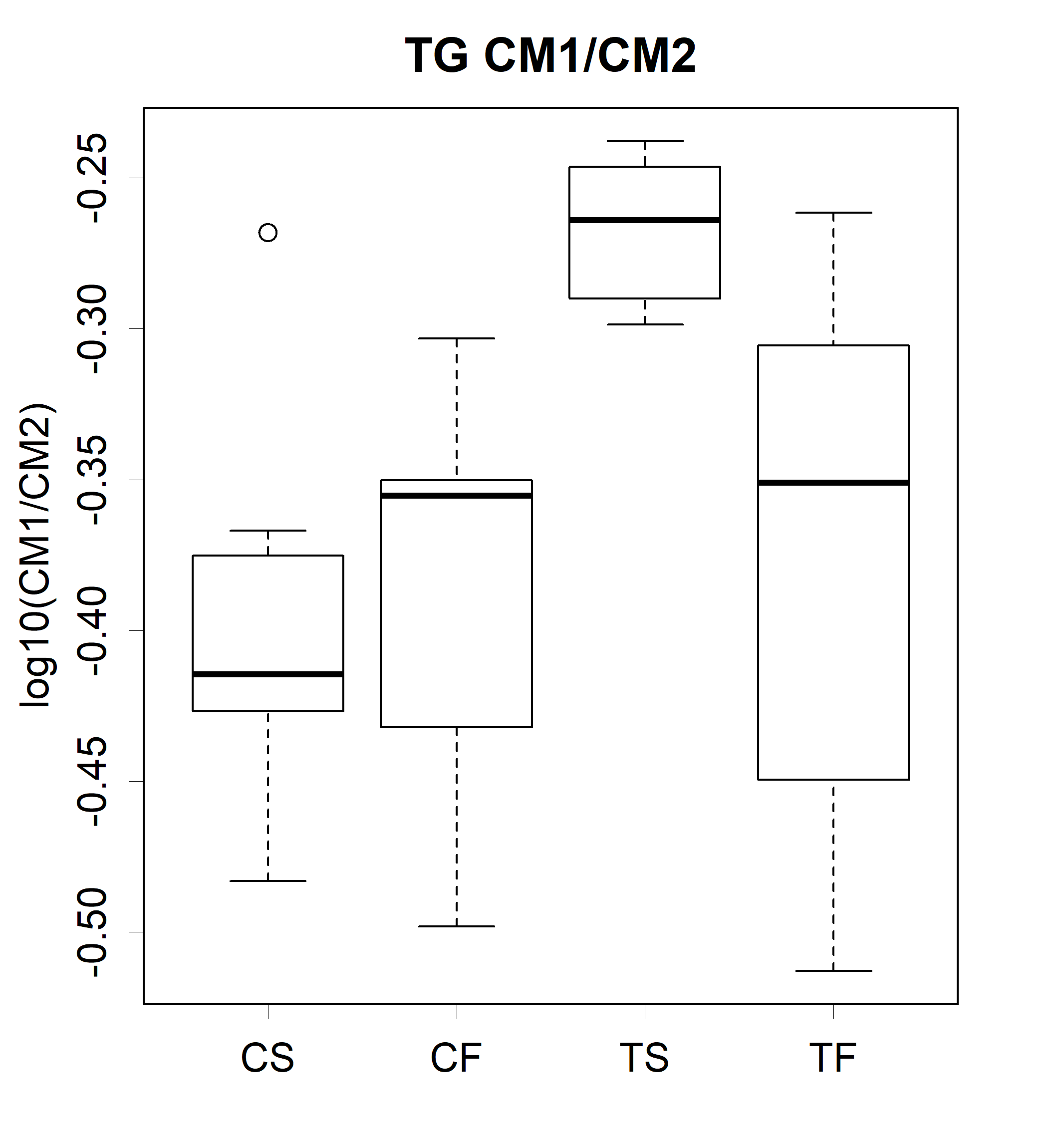

Supplement: S4 Fig — (ZIP) [file pone.0210950.s004.zip › S4_Fig/TG/TG_CM1_CM2.png]

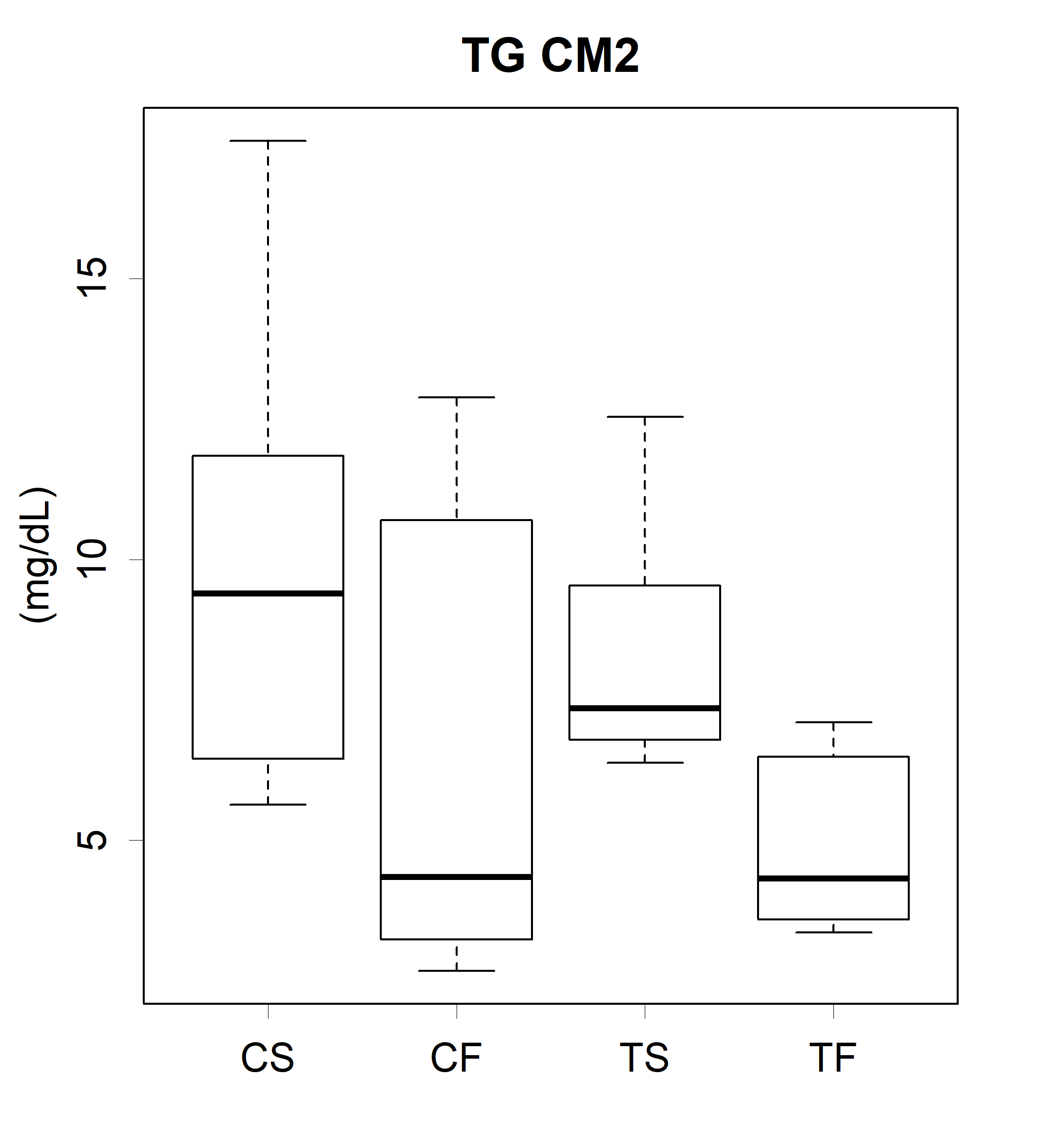

Supplement: S4 Fig — (ZIP) [file pone.0210950.s004.zip › S4_Fig/TG/TG_CM2.png]

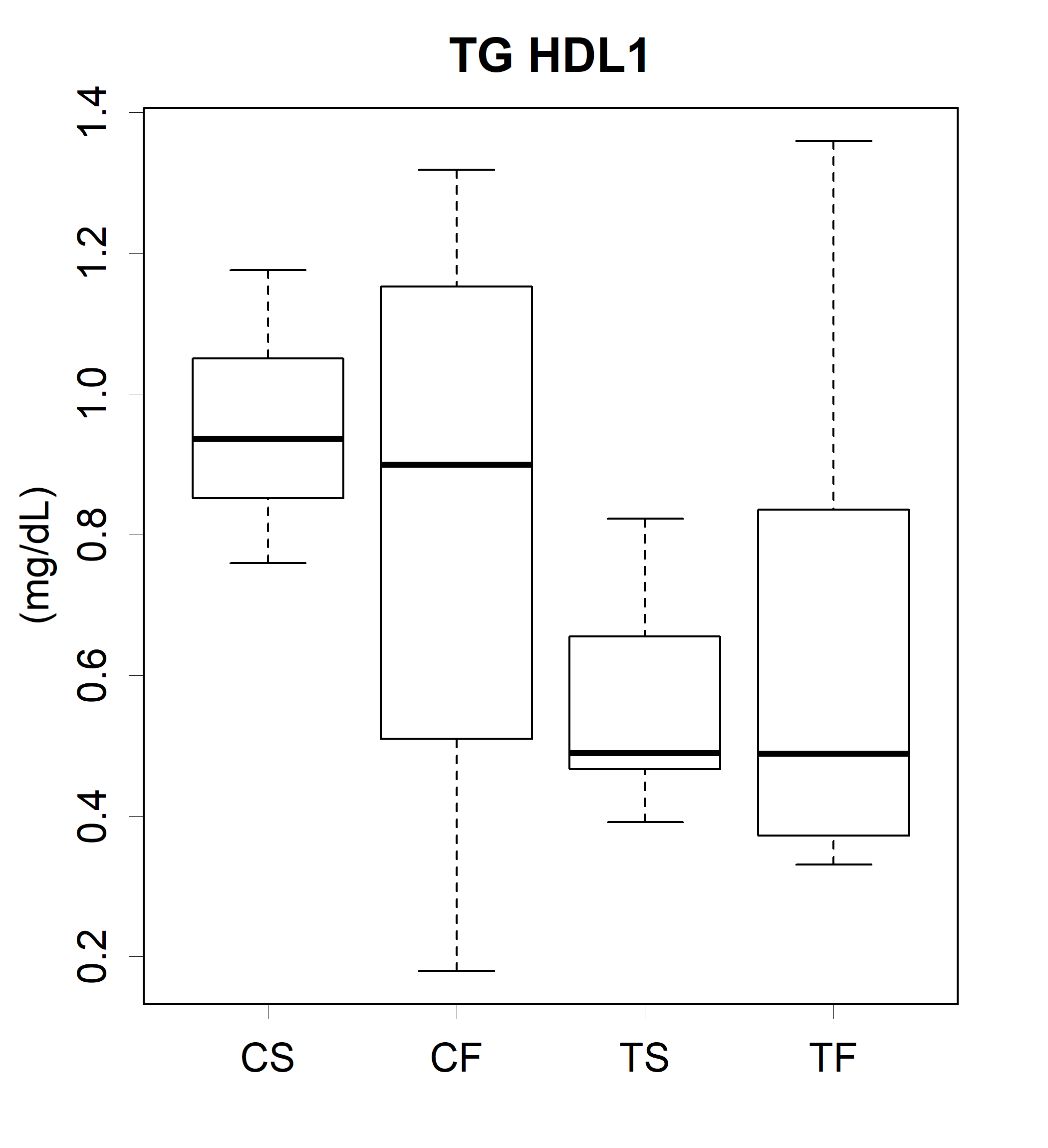

Supplement: S4 Fig — (ZIP) [file pone.0210950.s004.zip › S4_Fig/TG/TG_HDL1.png]

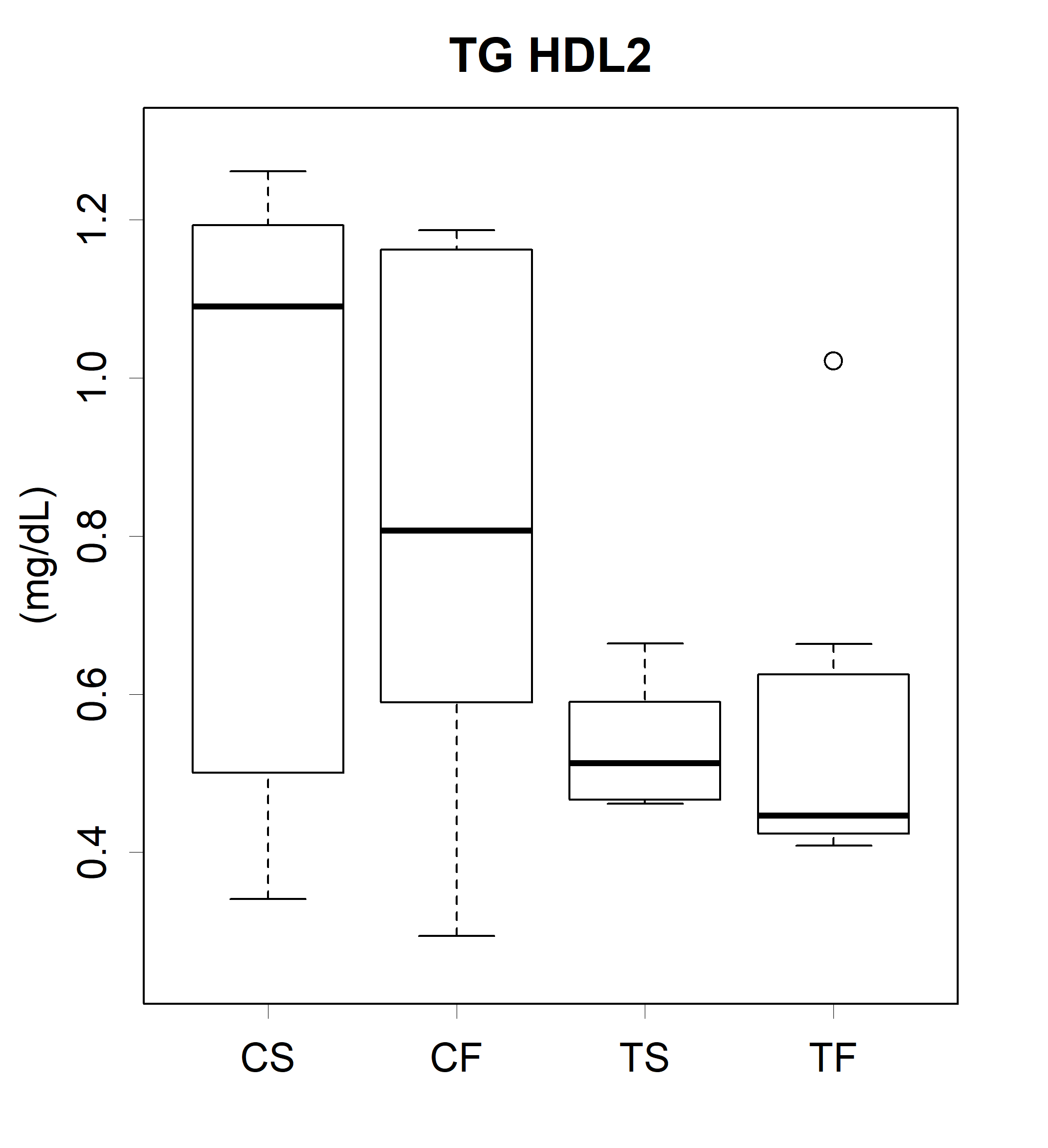

Supplement: S4 Fig — (ZIP) [file pone.0210950.s004.zip › S4_Fig/TG/TG_HDL2.png]

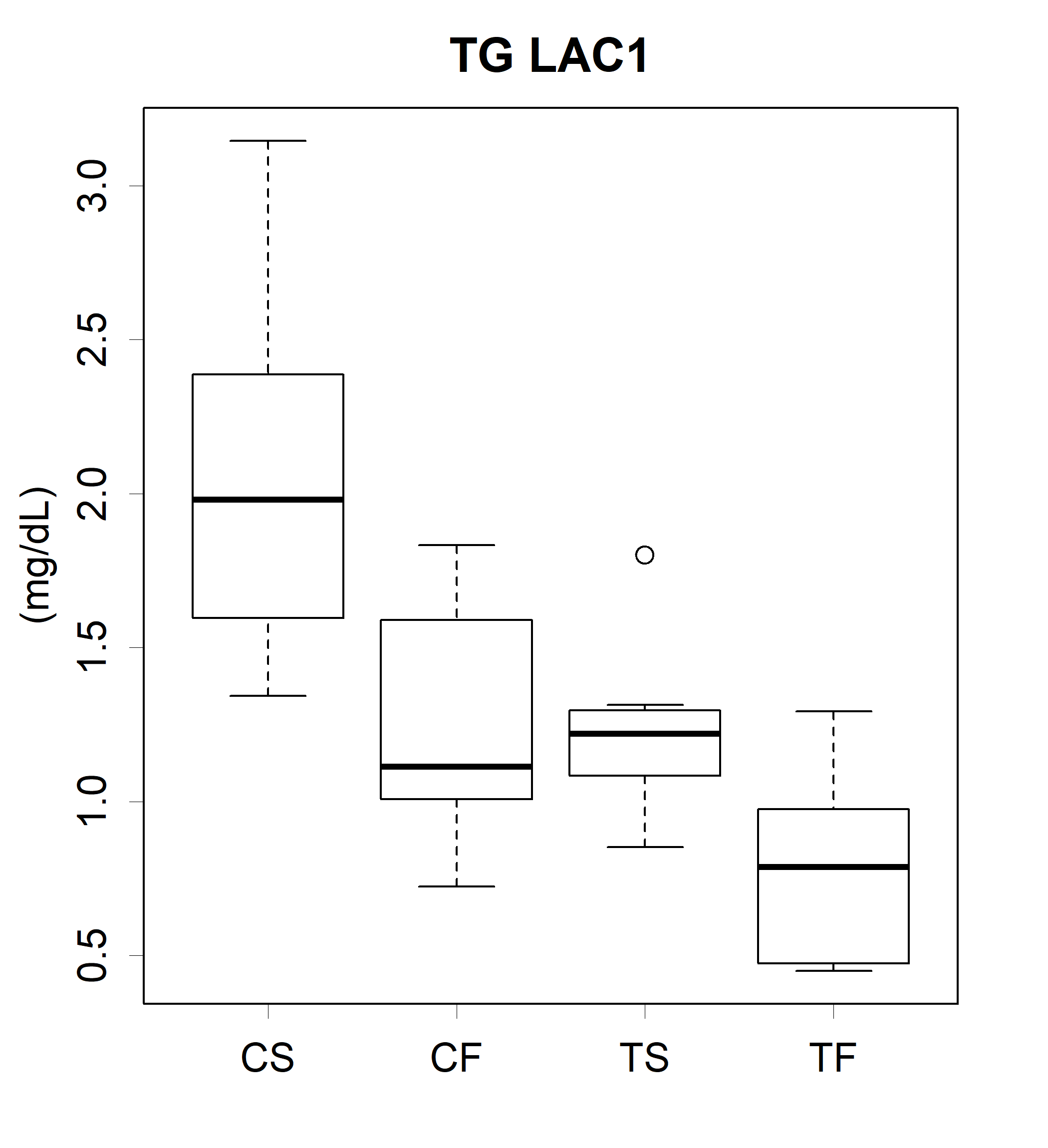

Supplement: S4 Fig — (ZIP) [file pone.0210950.s004.zip › S4_Fig/TG/TG_LAC1.png]

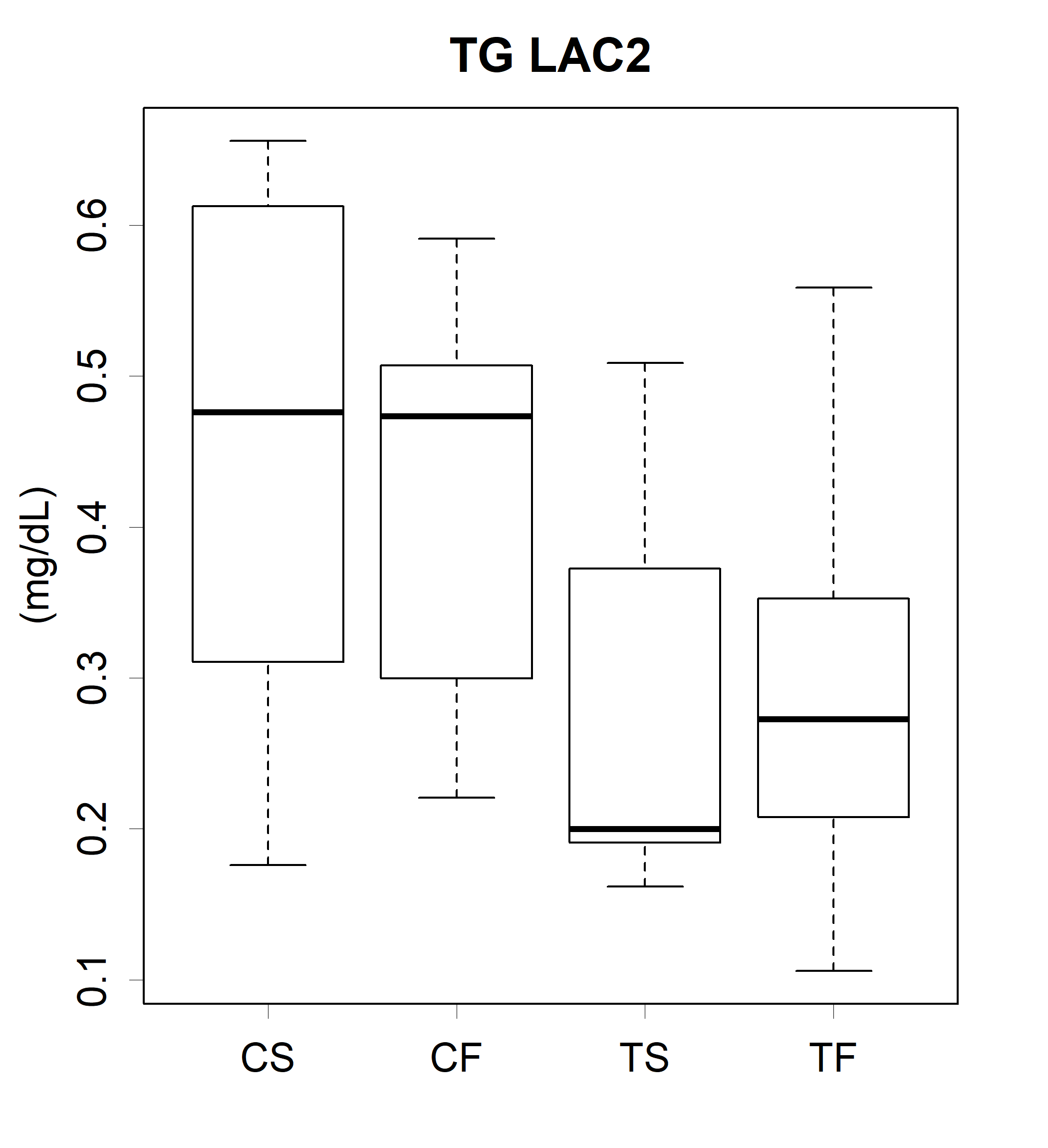

Supplement: S4 Fig — (ZIP) [file pone.0210950.s004.zip › S4_Fig/TG/TG_LAC2.png]

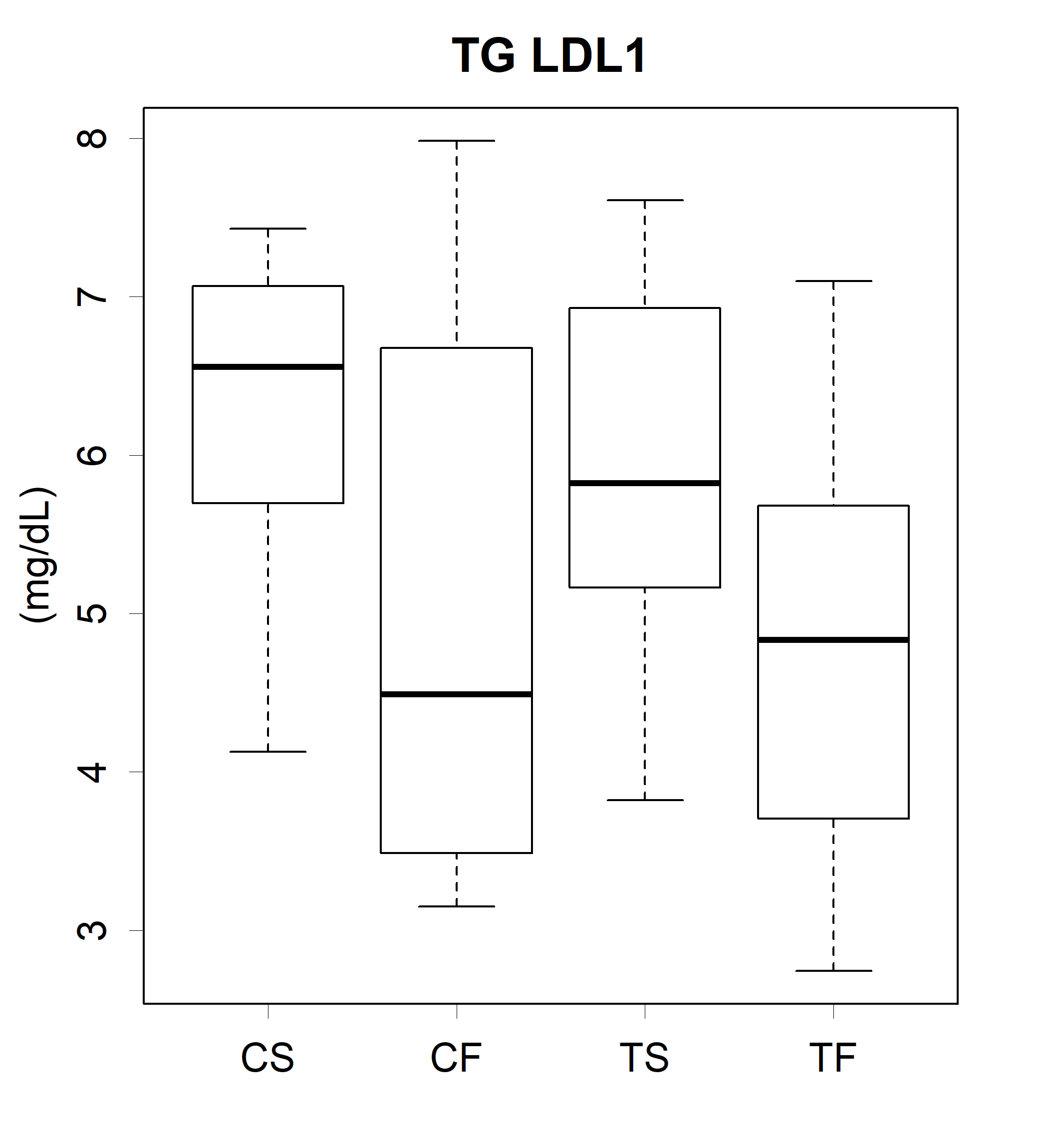

Supplement: S4 Fig — (ZIP) [file pone.0210950.s004.zip › S4_Fig/TG/TG_LDL1.png]

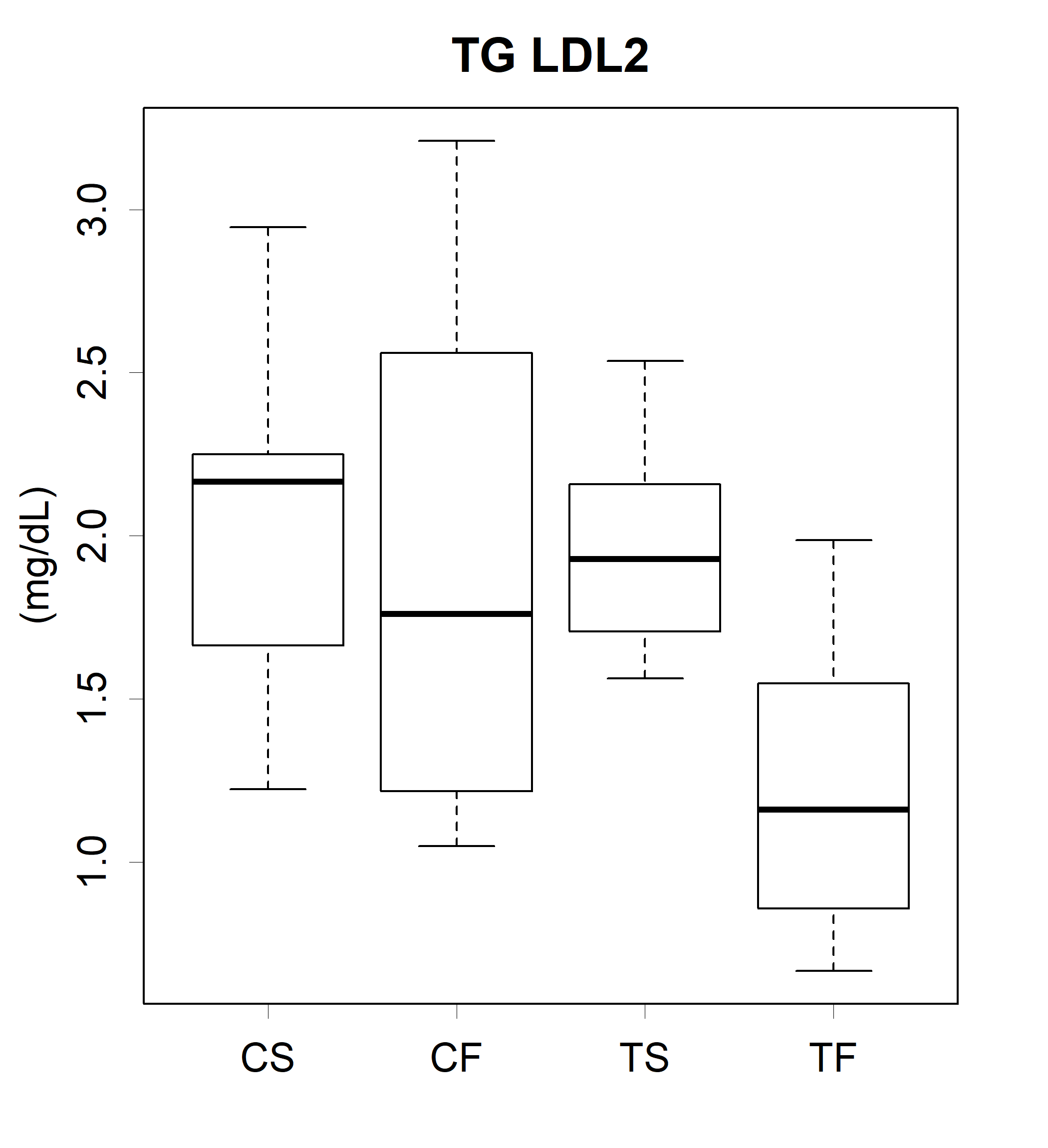

Supplement: S4 Fig — (ZIP) [file pone.0210950.s004.zip › S4_Fig/TG/TG_LDL2.png]

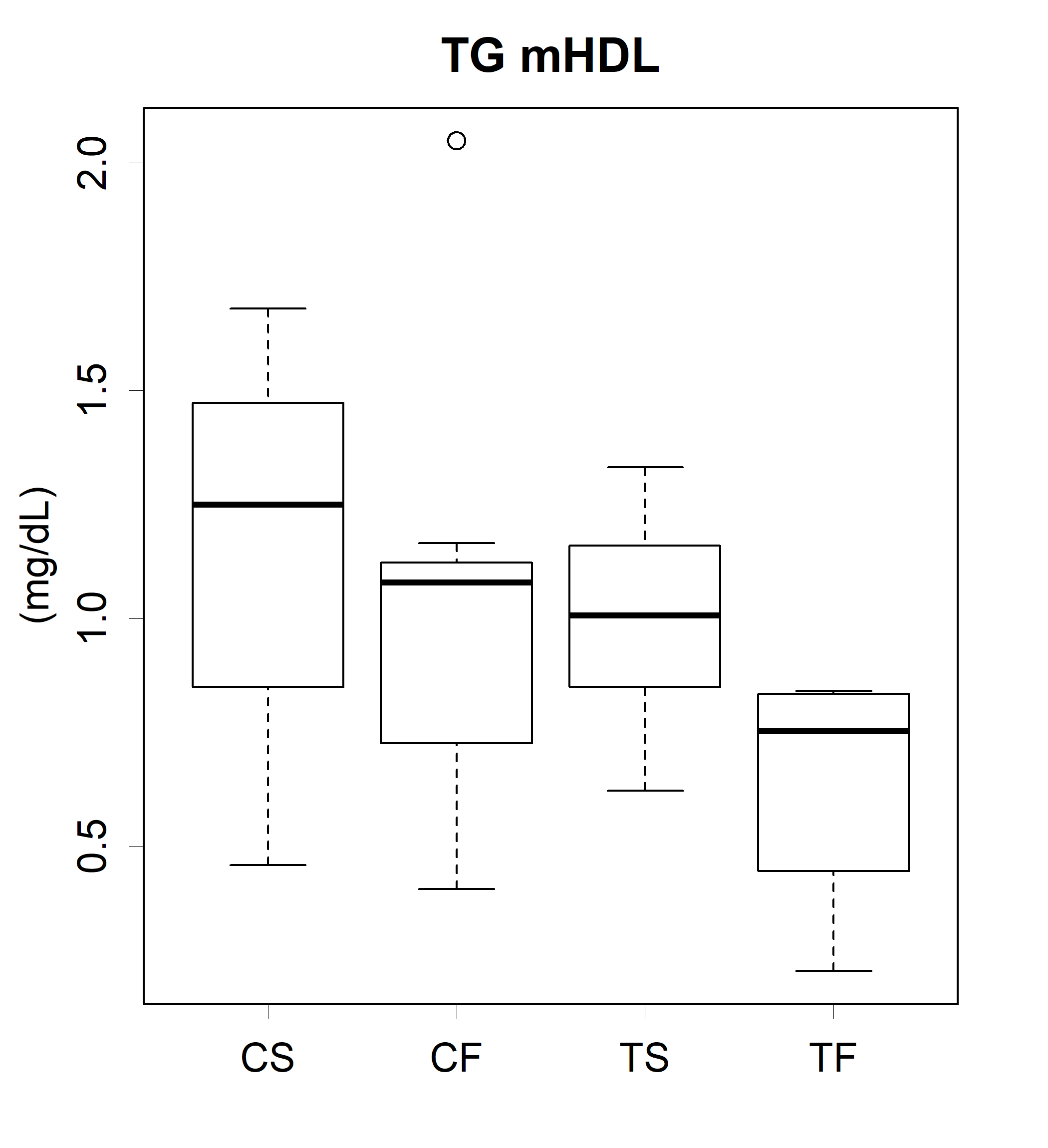

Supplement: S4 Fig — (ZIP) [file pone.0210950.s004.zip › S4_Fig/TG/TG_mHDL.png]

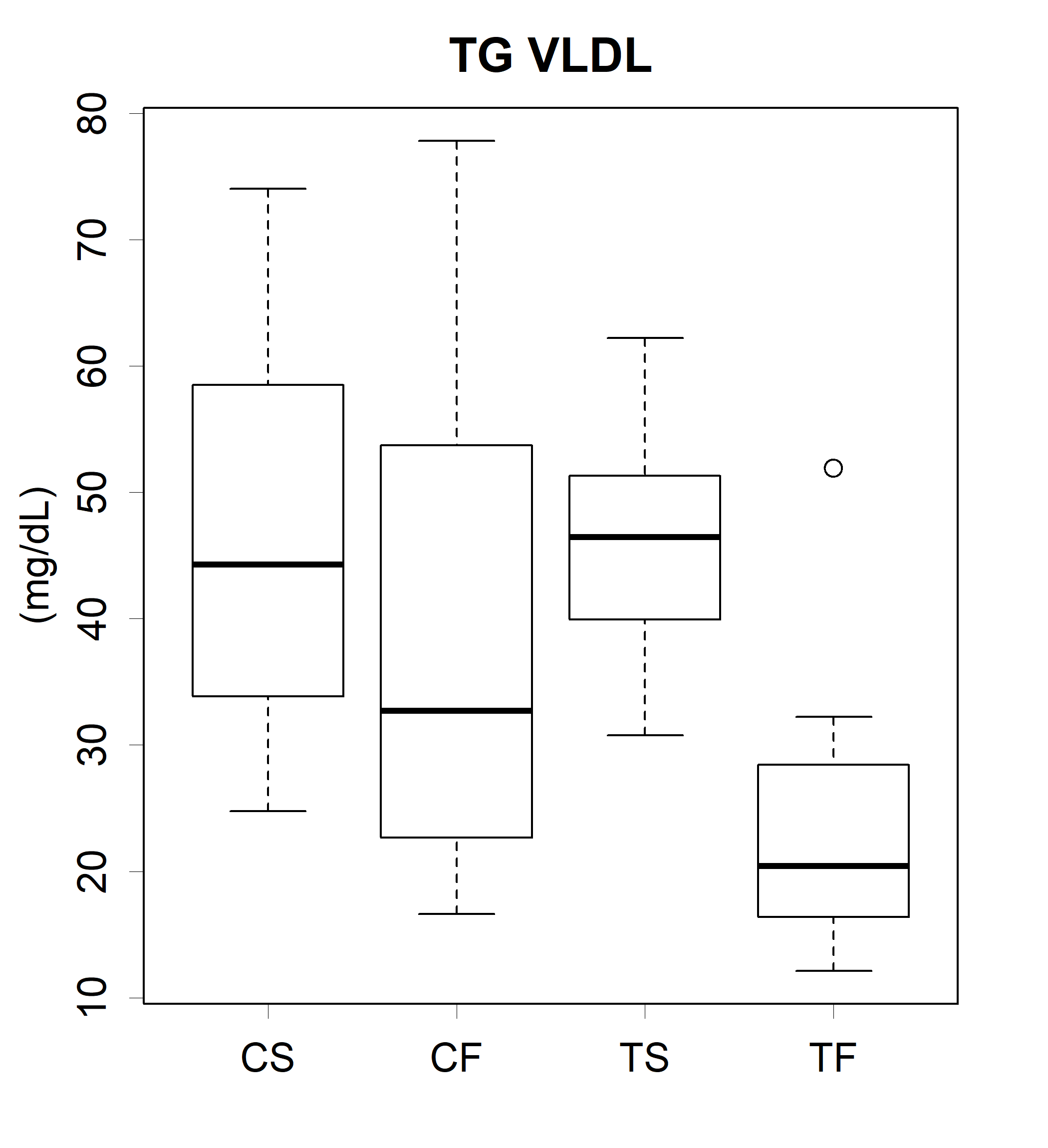

Supplement: S4 Fig — (ZIP) [file pone.0210950.s004.zip › S4_Fig/TG/TG_VLDL.png]

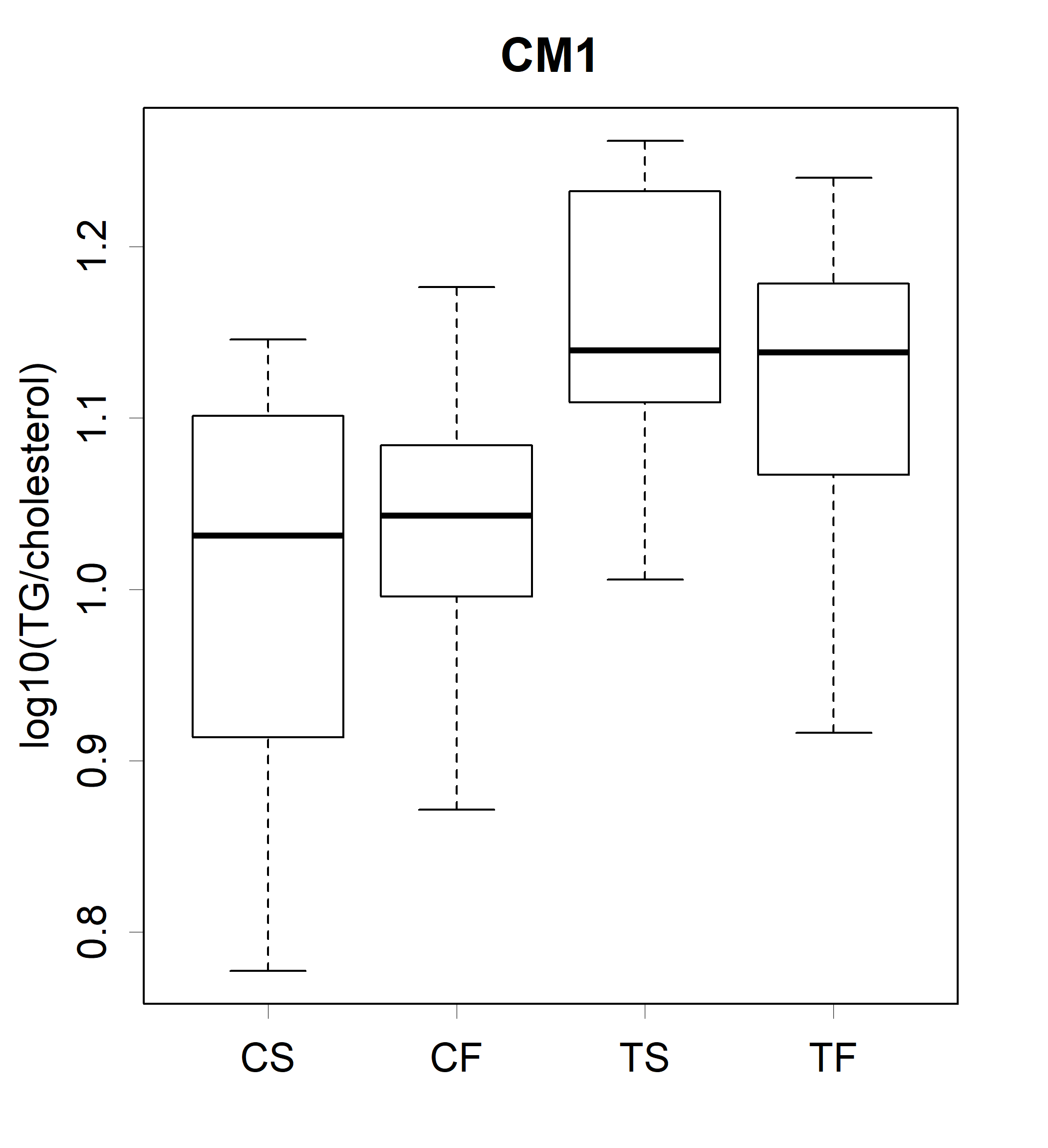

Supplement: S5 Fig — (ZIP) [file pone.0210950.s005.zip › S5_Fig/TG_cho/TG_cho_CM1.png]

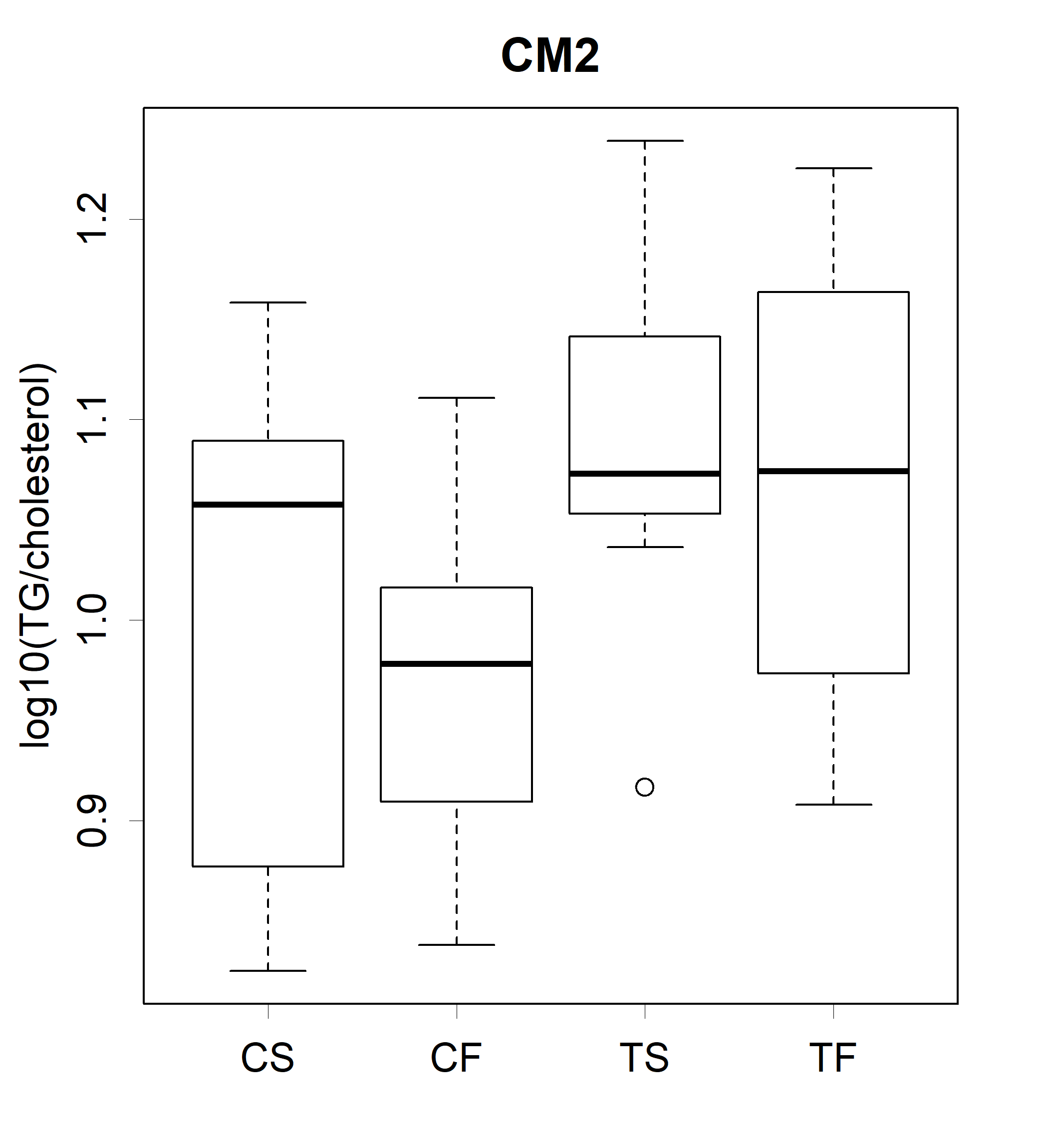

Supplement: S5 Fig — (ZIP) [file pone.0210950.s005.zip › S5_Fig/TG_cho/TG_cho_CM2.png]

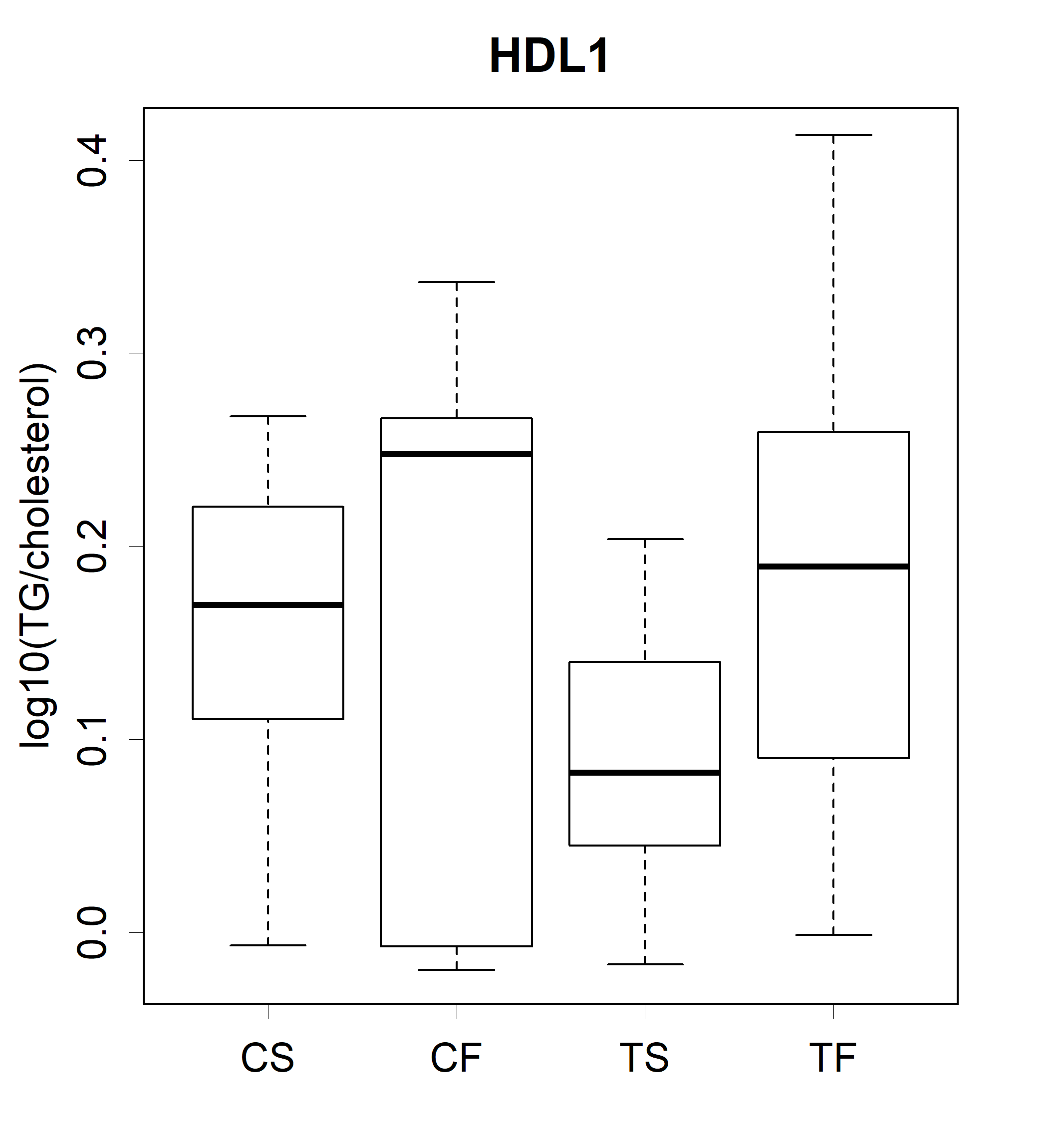

Supplement: S5 Fig — (ZIP) [file pone.0210950.s005.zip › S5_Fig/TG_cho/TG_cho_HDL1.png]

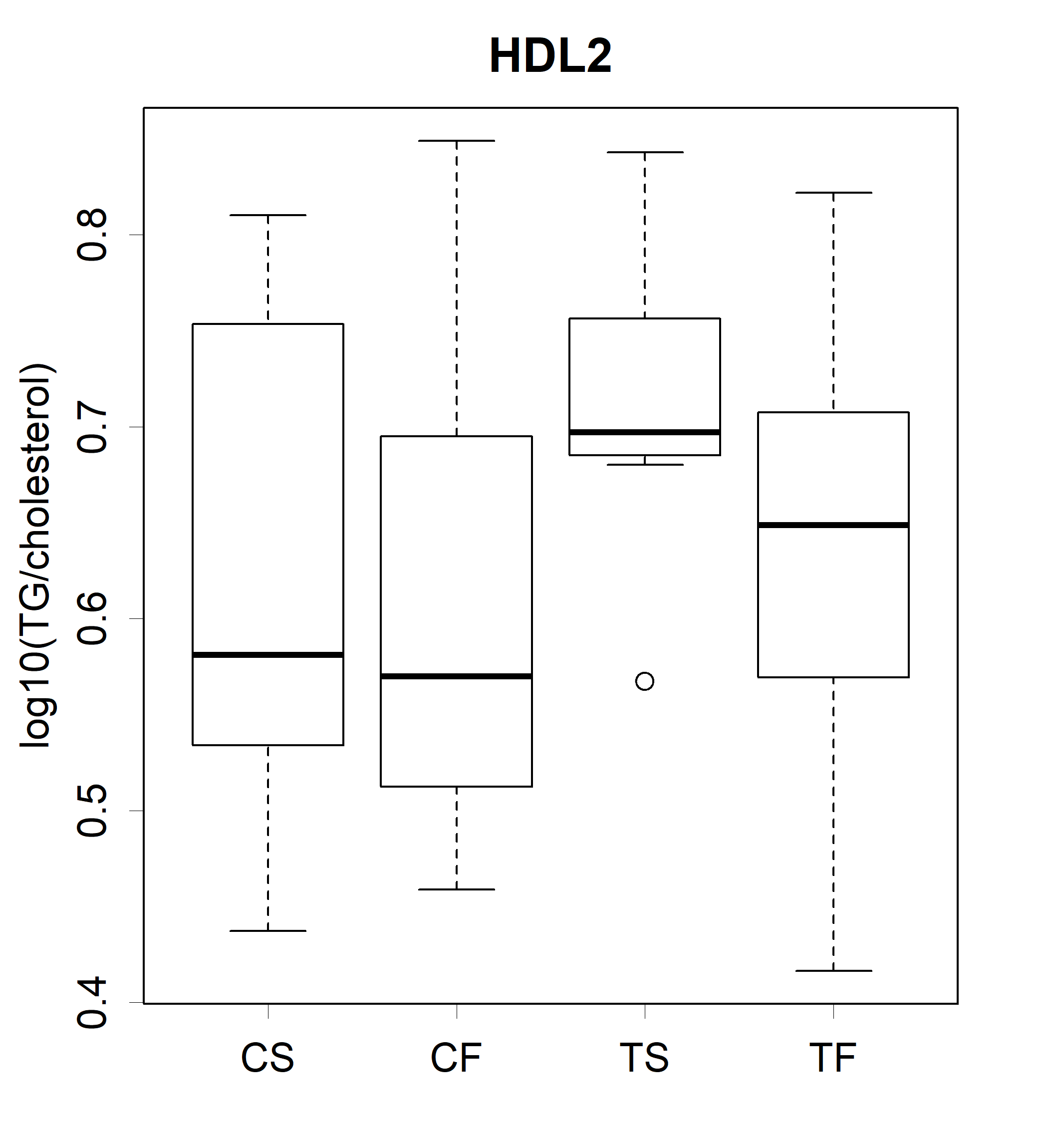

Supplement: S5 Fig — (ZIP) [file pone.0210950.s005.zip › S5_Fig/TG_cho/TG_cho_HDL2.png]

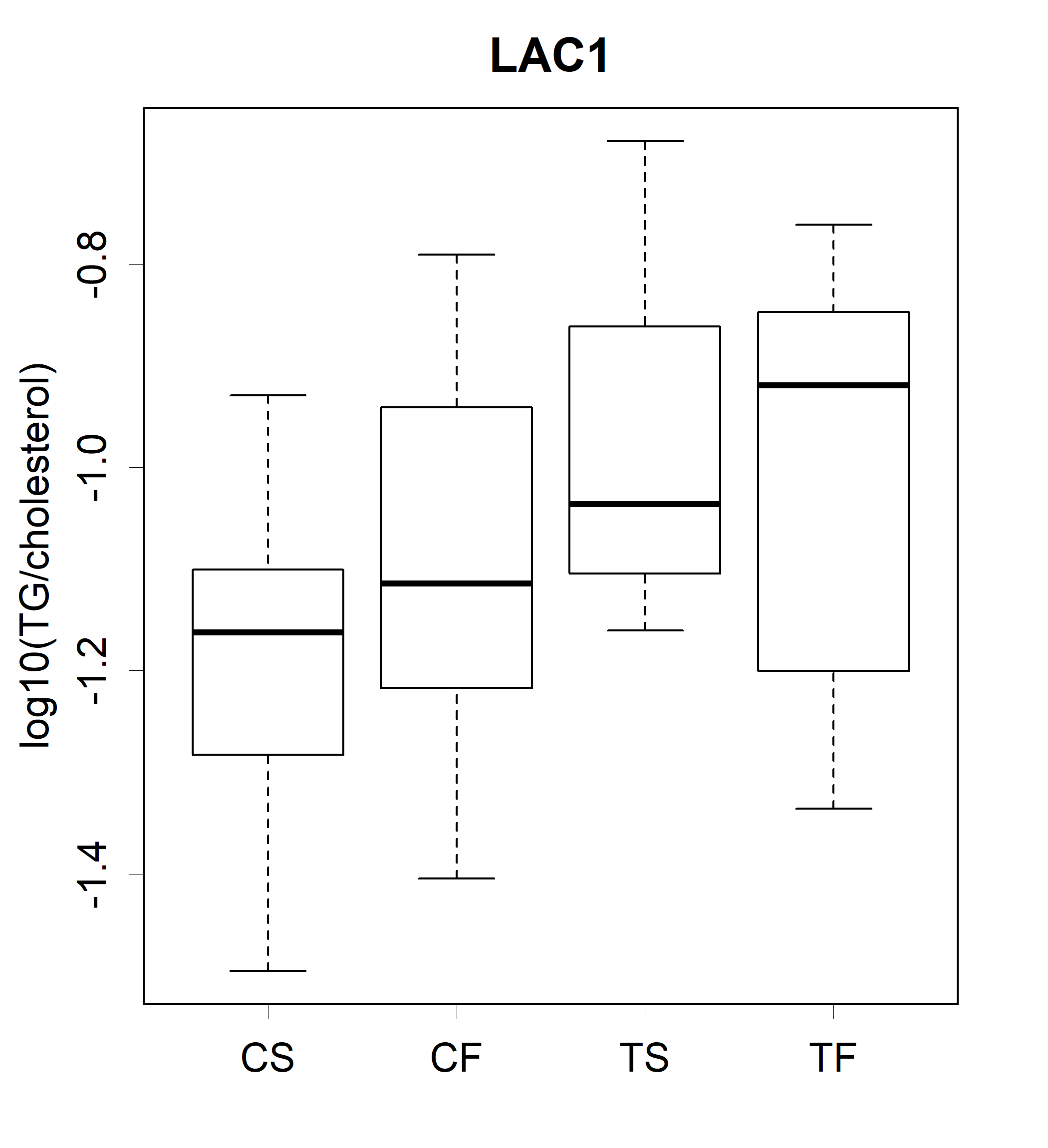

Supplement: S5 Fig — (ZIP) [file pone.0210950.s005.zip › S5_Fig/TG_cho/TG_cho_LAC1.png]

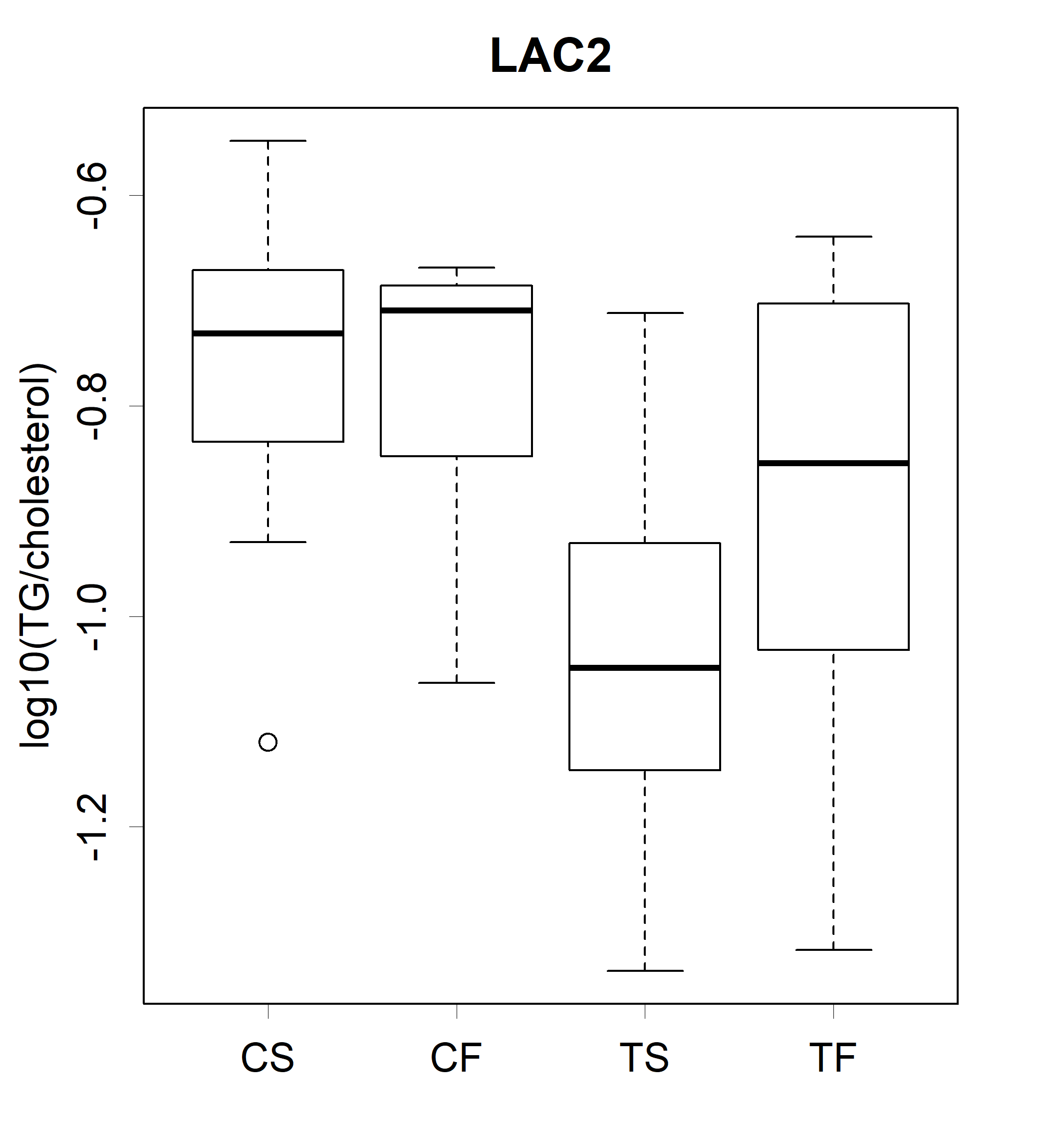

Supplement: S5 Fig — (ZIP) [file pone.0210950.s005.zip › S5_Fig/TG_cho/TG_cho_LAC2.png]

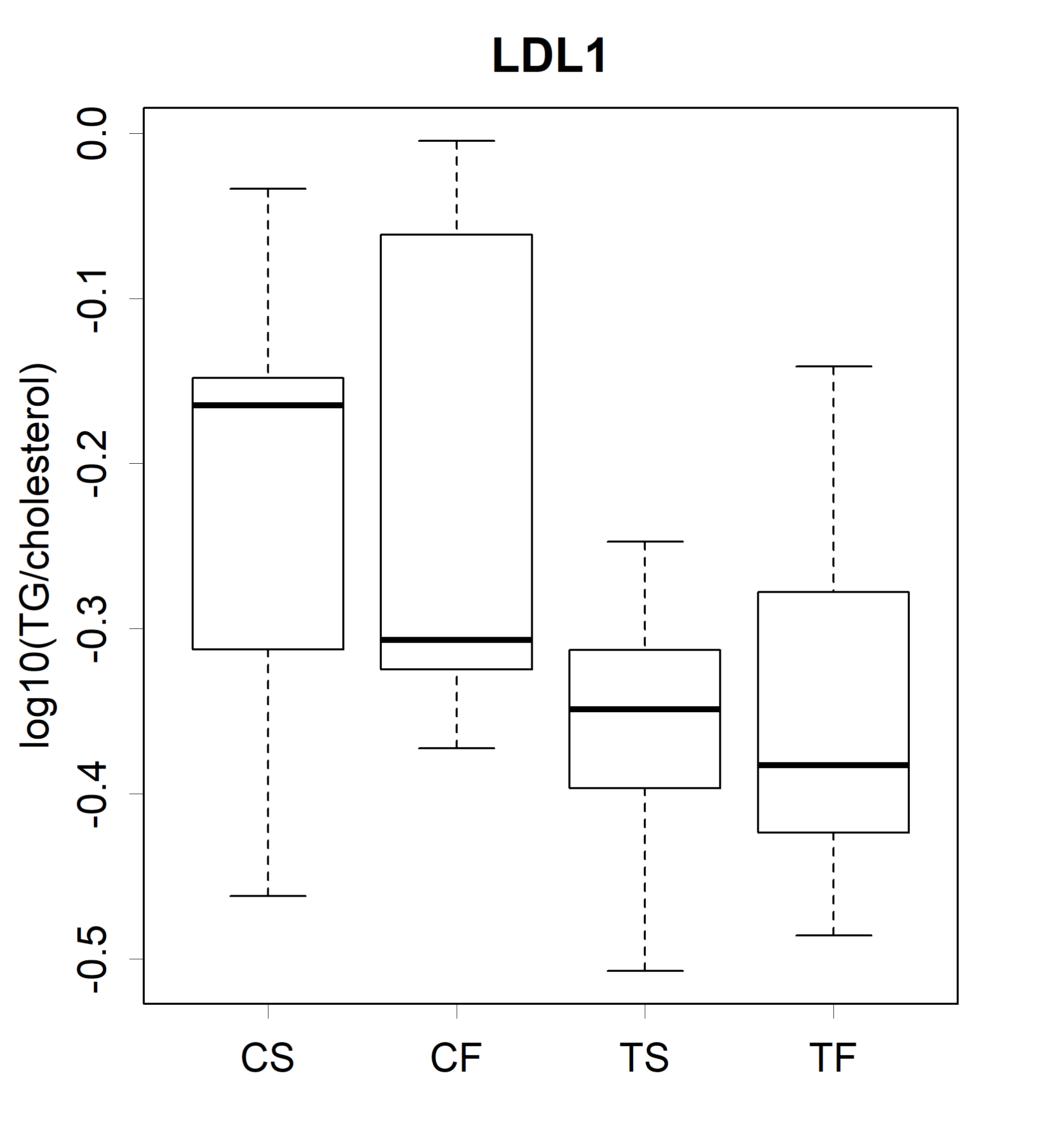

Supplement: S5 Fig — (ZIP) [file pone.0210950.s005.zip › S5_Fig/TG_cho/TG_cho_LDL1.png]

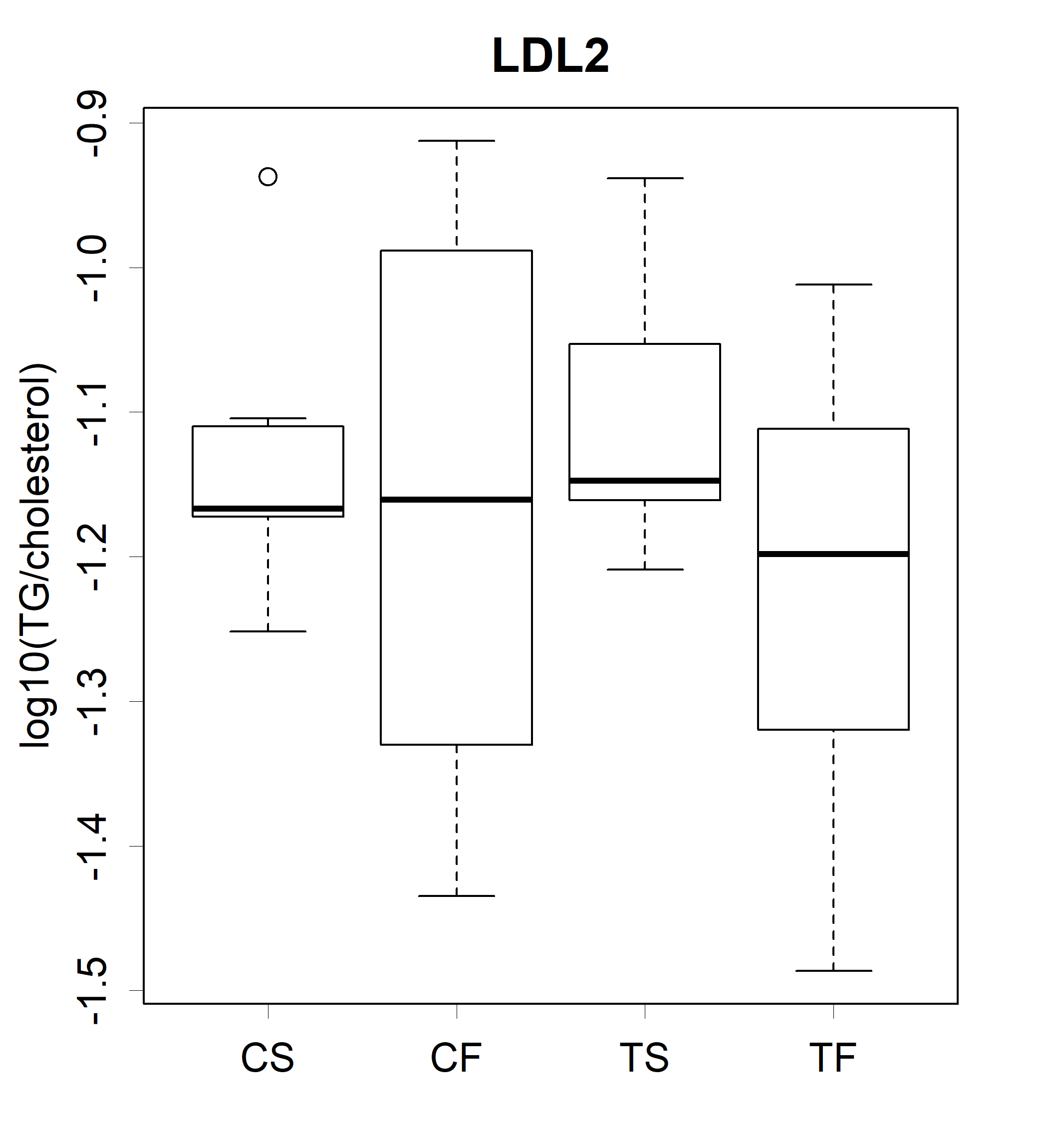

Supplement: S5 Fig — (ZIP) [file pone.0210950.s005.zip › S5_Fig/TG_cho/TG_cho_LDL2.png]

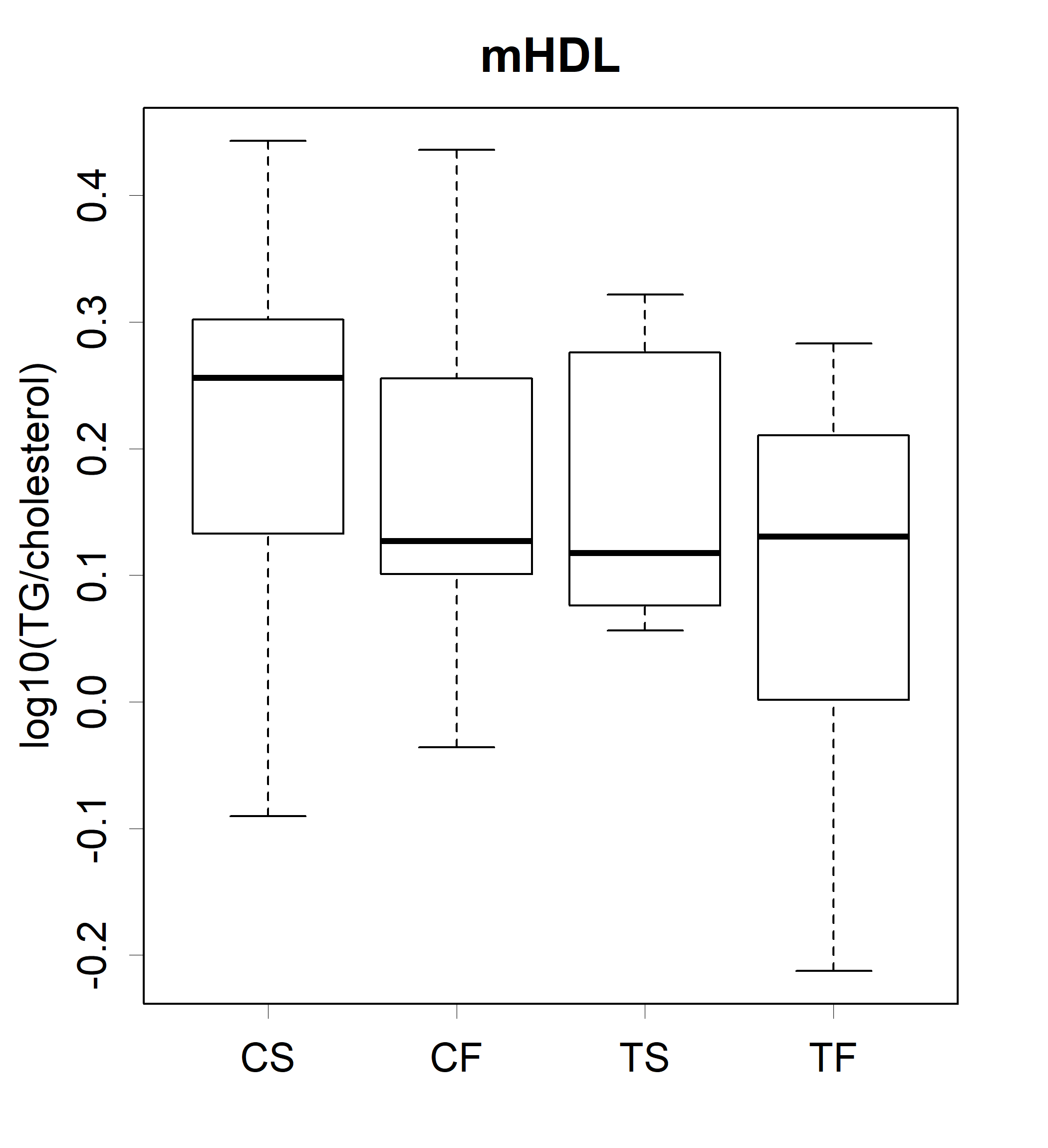

Supplement: S5 Fig — (ZIP) [file pone.0210950.s005.zip › S5_Fig/TG_cho/TG_cho_mHDL.png]

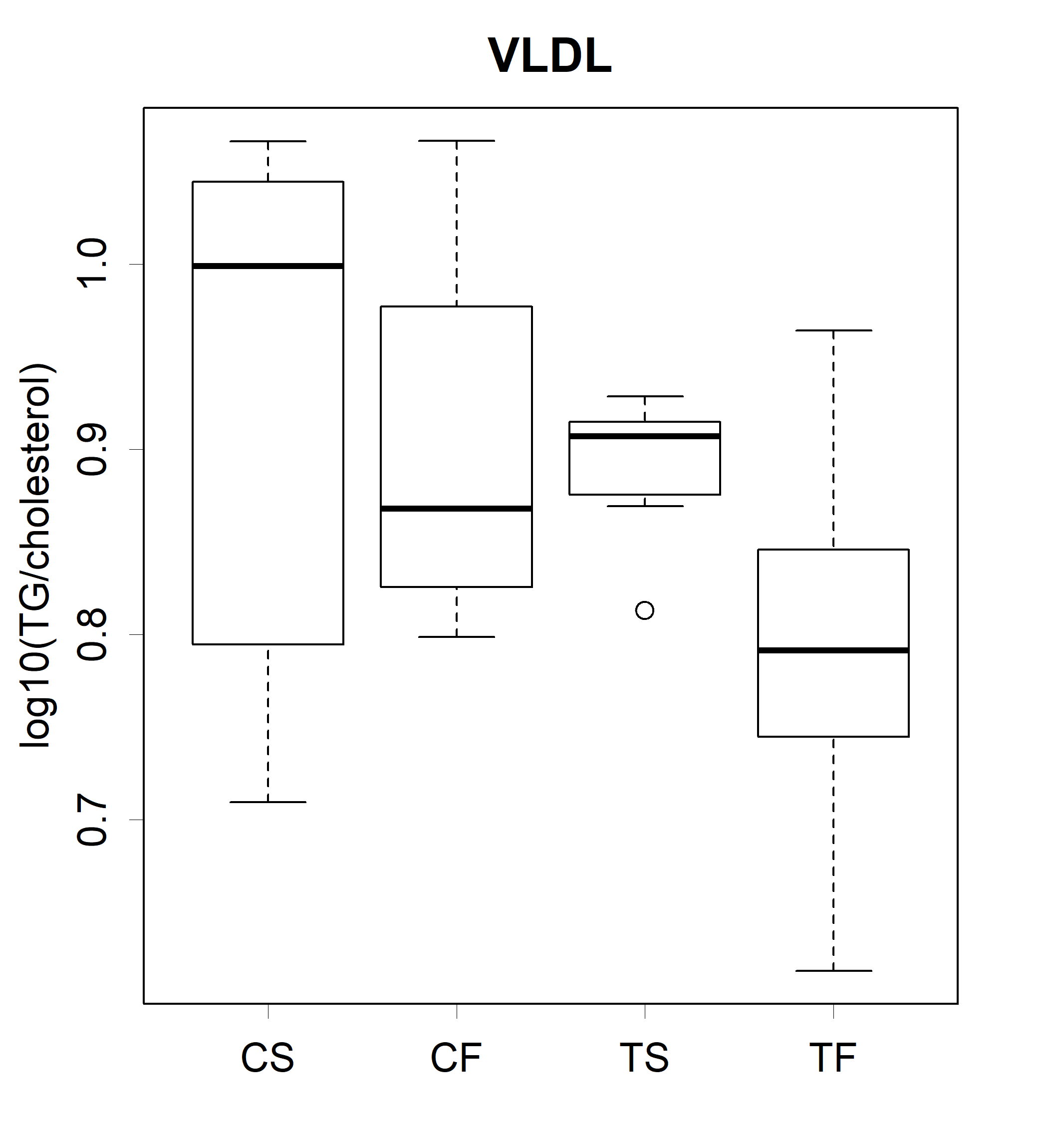

Supplement: S5 Fig — (ZIP) [file pone.0210950.s005.zip › S5_Fig/TG_cho/TG_cho_VLDL.png]

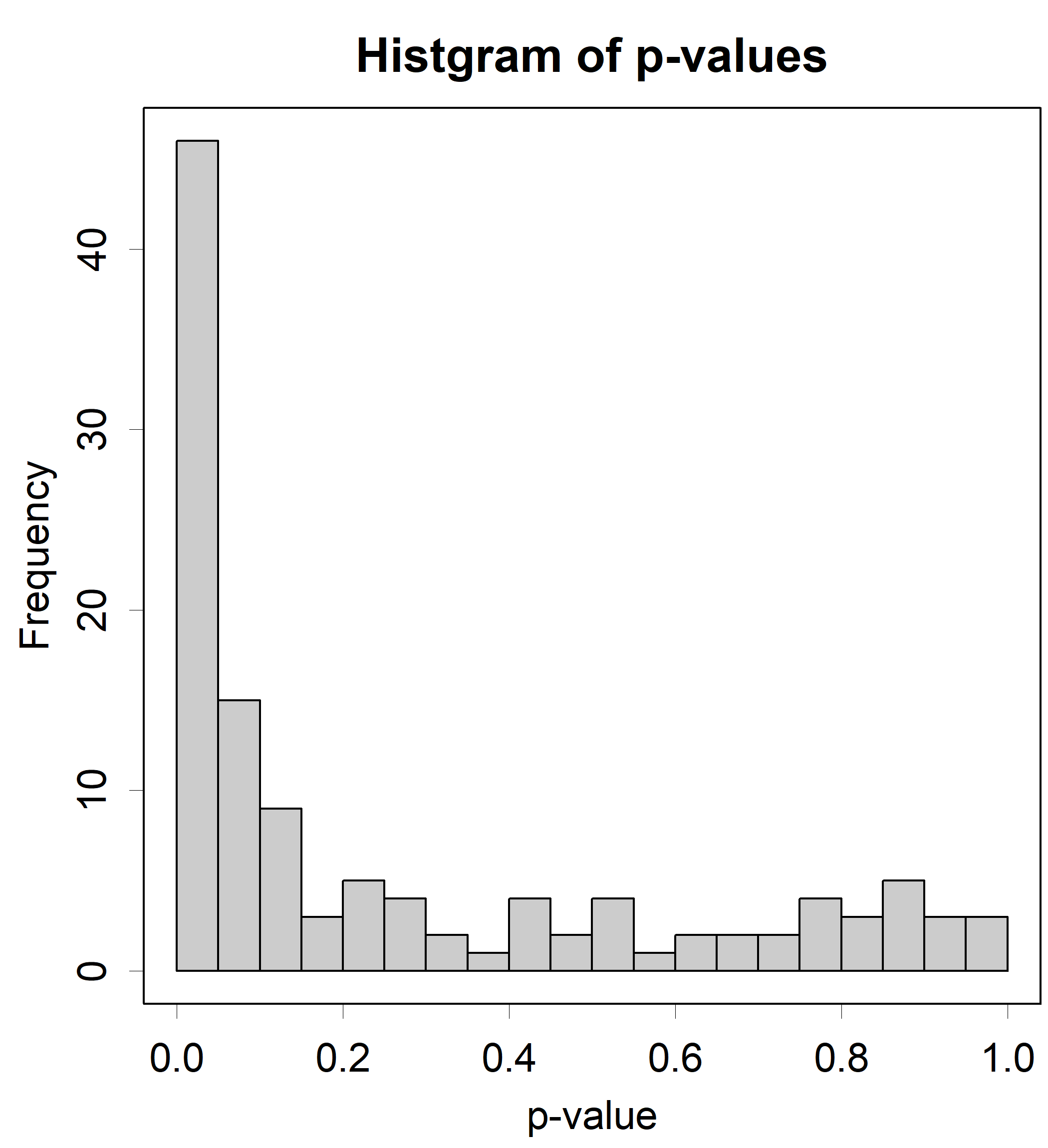

Supplement: S1 Appendix — (ZIP) [file pone.0210950.s014.zip › S1 Appendix/PvalHist.png]

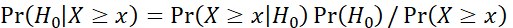

Supplement: S1 Appendix — (ZIP) [file pone.0210950.s014.zip › S1 Appendix/S1 Appendix.files/image001.png]

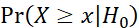

Supplement: S1 Appendix — (ZIP) [file pone.0210950.s014.zip › S1 Appendix/S1 Appendix.files/image003.png]

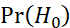

Supplement: S1 Appendix — (ZIP) [file pone.0210950.s014.zip › S1 Appendix/S1 Appendix.files/image005.png]

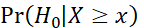

Supplement: S1 Appendix — (ZIP) [file pone.0210950.s014.zip › S1 Appendix/S1 Appendix.files/image007.png]

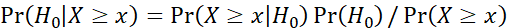

Supplement: S1 Appendix — (ZIP) [file pone.0210950.s014.zip › S1 Appendix/S1 Appendix.files/image010.png]

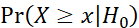

Supplement: S1 Appendix — (ZIP) [file pone.0210950.s014.zip › S1 Appendix/S1 Appendix.files/image012.png]

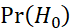

Supplement: S1 Appendix — (ZIP) [file pone.0210950.s014.zip › S1 Appendix/S1 Appendix.files/image013.png]

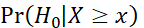

Supplement: S1 Appendix — (ZIP) [file pone.0210950.s014.zip › S1 Appendix/S1 Appendix.files/image014.png]

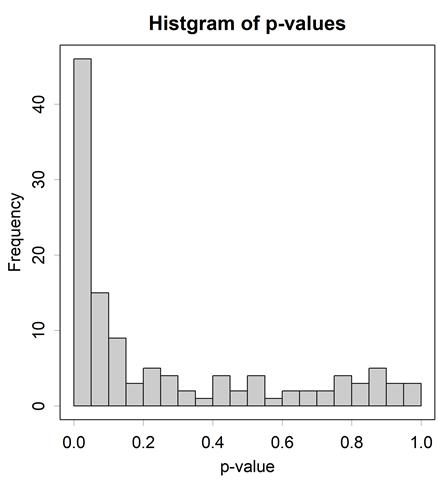

Supplement: S1 Appendix — (ZIP) [file pone.0210950.s014.zip › S1 Appendix/S1 Appendix.files/image015.jpg]
