# Supplementary material for: Tofu and fish oil independently modulate serum lipid profiles in rats: Analyses of 10 class lipoprotein profiles and the global hepatic transcriptome
Source: PLoS One. 2019 Jan 17;14(1):e0210950. doi: 10.1371/journal.pone.0210950 (PMC6336308; doi:10.1371/journal.pone.0210950)
Supplement: S2 Fig — (ZIP) [file pone.0210950.s002.zip › S2_Fig/time/CM1.htm]

# CM1

**ANOVA p-value**: 0.00831   
  
Tukey multiple comparisons of means   
95% family-wise confidence level

| combinations | diff | lwr | upr | p adj |
| --- | --- | --- | --- | --- |
| 2-1 | 0.0094878407 | -0.033965783 | 0.05294146 | 0.9309114 |
| 3-1 | -0.0004924358 | -0.043946060 | 0.04296119 | 0.9999888 |
| 4-1 | 0.0491677402 | 0.007093950 | 0.09124153 | 0.0176372 |
| 3-2 | -0.0099802766 | -0.053433900 | 0.03347335 | 0.9208131 |
| 4-2 | 0.0396798994 | -0.002393891 | 0.08175369 | 0.0696017 |
| 4-3 | 0.0496601760 | 0.007586386 | 0.09173397 | 0.0163594 |

**Groups** 1: CS, 2: CF, 3: TS, 4: TF   
  
back to the summary page
