# Supplementary material for: Tofu and fish oil independently modulate serum lipid profiles in rats: Analyses of 10 class lipoprotein profiles and the global hepatic transcriptome
Source: PLoS One. 2019 Jan 17;14(1):e0210950. doi: 10.1371/journal.pone.0210950 (PMC6336308; doi:10.1371/journal.pone.0210950)
Supplement: S2 Fig — (ZIP) [file pone.0210950.s002.zip › S2_Fig/time/LAC2.htm]

# LAC2

**ANOVA p-value**:0.003055   
  
Tukey multiple comparisons of means   
95% family-wise confidence level

| combinations | diff | lwr | upr | p adj |
| --- | --- | --- | --- | --- |
| 2-1 | -0.0181208737 | -0.04895062 | 0.012708868 | 0.3877928 |
| 3-1 | -0.0322814382 | -0.06311118 | -0.001451697 | 0.0376990 |
| 4-1 | -0.0332076676 | -0.06305844 | -0.003356899 | 0.0251809 |
| 3-2 | -0.0141605645 | -0.04499031 | 0.016669177 | 0.5937995 |
| 4-2 | -0.0150867940 | -0.04493756 | 0.014763975 | 0.5168870 |
| 4-3 | -0.0009262295 | -0.03077700 | 0.028924539 | 0.9997708 |

**Groups** 1: CS, 2: CF, 3: TS, 4: TF   
  
back to the summary page
