# Supplementary material for: Tofu and fish oil independently modulate serum lipid profiles in rats: Analyses of 10 class lipoprotein profiles and the global hepatic transcriptome
Source: PLoS One. 2019 Jan 17;14(1):e0210950. doi: 10.1371/journal.pone.0210950 (PMC6336308; doi:10.1371/journal.pone.0210950)
Supplement: S2 Fig — (ZIP) [file pone.0210950.s002.zip › S2_Fig/time/LDL1.htm]

# LDL1

**ANOVA p-value**:0.00004652   
  
Tukey multiple comparisons of means   
95% family-wise confidence level

| combinations | diff | lwr | upr | p adj |
| --- | --- | --- | --- | --- |
| 2-1 | -0.053503356 | -0.14445871 | 0.03745200 | 0.3871108 |
| 3-1 | -0.155510723 | -0.24646608 | -0.06455537 | 0.0004409 |
| 4-1 | -0.163317844 | -0.25138499 | -0.07525070 | 0.0001595 |
| 3-2 | -0.102007367 | -0.19296272 | -0.01105201 | 0.0237890 |
| 4-2 | -0.109814488 | -0.19788163 | -0.02174734 | 0.0106002 |
| 4-3 | -0.007807121 | -0.09587427 | 0.08026002 | 0.9947697 |

**Groups** 1: CS, 2: CF, 3: TS, 4: TF   
  
back to the summary page
