# Supplementary material for: Tofu and fish oil independently modulate serum lipid profiles in rats: Analyses of 10 class lipoprotein profiles and the global hepatic transcriptome
Source: PLoS One. 2019 Jan 17;14(1):e0210950. doi: 10.1371/journal.pone.0210950 (PMC6336308; doi:10.1371/journal.pone.0210950)
Supplement: S2 Fig — (ZIP) [file pone.0210950.s002.zip › S2_Fig/time/LDL2.htm]

# LDL2

**ANOVA p-value**:0.02158   
  
Tukey multiple comparisons of means   
95% family-wise confidence level

| combinations | diff | lwr | upr | p adj |
| --- | --- | --- | --- | --- |
| 2-1 | 0.01505972 | -0.031861085 | 0.06198052 | 0.8136878 |
| 3-1 | 0.05559229 | 0.008671488 | 0.10251309 | 0.0158918 |
| 4-1 | 0.03176666 | -0.013664206 | 0.07719754 | 0.2441331 |
| 3-2 | 0.04053257 | -0.006388228 | 0.08745337 | 0.1079817 |
| 4-2 | 0.01670695 | -0.028723922 | 0.06213782 | 0.7442523 |
| 4-3 | -0.02382562 | -0.069256495 | 0.02160525 | 0.4858201 |

**Groups** 1: CS, 2: CF, 3: TS, 4: TF   
  
back to the summary page
