# Supplementary material for: Tofu and fish oil independently modulate serum lipid profiles in rats: Analyses of 10 class lipoprotein profiles and the global hepatic transcriptome
Source: PLoS One. 2019 Jan 17;14(1):e0210950. doi: 10.1371/journal.pone.0210950 (PMC6336308; doi:10.1371/journal.pone.0210950)
Supplement: S2 Fig — (ZIP) [file pone.0210950.s002.zip › S2_Fig/time/VLDL.htm]

# VLDL

**ANOVA p-value**: 0.00006365   
  
Tukey multiple comparisons of means   
95% family-wise confidence level

| combinations | diff | lwr | upr | p adj |
| --- | --- | --- | --- | --- |
| 2-1 | 0.16737864 | -0.03891216 | 0.3736694 | 0.1422006 |
| 3-1 | 0.26206037 | 0.05576957 | 0.4683512 | 0.0090822 |
| 4-1 | 0.41611112 | 0.21637091 | 0.6158513 | 0.0000322 |
| 3-2 | 0.09468173 | -0.11160907 | 0.3009725 | 0.5943760 |
| 4-2 | 0.24873248 | 0.04899228 | 0.4484727 | 0.0107167 |
| 4-3 | 0.15405075 | -0.04568946 | 0.3537910 | 0.1738935 |

**Groups** 1: CS, 2: CF, 3: TS, 4: TF   
  
back to the summary page
