# Supplementary material for: Tofu and fish oil independently modulate serum lipid profiles in rats: Analyses of 10 class lipoprotein profiles and the global hepatic transcriptome
Source: PLoS One. 2019 Jan 17;14(1):e0210950. doi: 10.1371/journal.pone.0210950 (PMC6336308; doi:10.1371/journal.pone.0210950)
Supplement: S3 Fig — (ZIP) [file pone.0210950.s003.zip › S3_Fig/Ch/CM2.htm]

# CM2

**ANOVA p-value**:0.00393
  
  
Tukey multiple comparisons of means   
95% family-wise confidence level

| combinations | diff | lwr | upr | p adj |
| --- | --- | --- | --- | --- |
| 2-1 | -0.57117176 | -1.3429124 | 0.2005689 | 0.2020985 |
| 3-1 | -0.59742038 | -1.3691610 | 0.1743203 | 0.1714604 |
| 4-1 | -1.12686076 | -1.8740954 | -0.3796261 | 0.0018046 |
| 3-2 | -0.02624863 | -0.7979893 | 0.7454920 | 0.9996983 |
| 4-2 | -0.55568900 | -1.3029237 | 0.1915457 | 0.1987173 |
| 4-3 | -0.52944037 | -1.2766751 | 0.2177943 | 0.2340583 |

**Groups** 1: CS, 2: CF, 3: TS, 4: TF   
  
back to the summary page
