# Supplementary material for: Tofu and fish oil independently modulate serum lipid profiles in rats: Analyses of 10 class lipoprotein profiles and the global hepatic transcriptome
Source: PLoS One. 2019 Jan 17;14(1):e0210950. doi: 10.1371/journal.pone.0210950 (PMC6336308; doi:10.1371/journal.pone.0210950)
Supplement: S3 Fig — (ZIP) [file pone.0210950.s003.zip › S3_Fig/Ch/HDL1.htm]

# HDL1

**ANOVA p-value**:0.02691
  
  
Tukey multiple comparisons of means   
95% family-wise confidence level

| combinations | diff | lwr | upr | p adj |
| --- | --- | --- | --- | --- |
| 2-1 | -0.23337454 | -0.6535635 | 0.18681443 | 0.4367010 |
| 3-1 | -0.48198177 | -0.9021707 | -0.06179280 | 0.0202389 |
| 4-1 | -0.57775514 | -0.9846014 | -0.17090891 | 0.0033082 |
| 3-2 | -0.24860723 | -0.6687962 | 0.17158174 | 0.3821175 |
| 4-2 | -0.34438060 | -0.7512268 | 0.06246563 | 0.1184649 |
| 4-3 | -0.09577337 | -0.5026196 | 0.31107286 | 0.9154224 |

**Groups** 1: CS, 2: CF, 3: TS, 4: TF   
  
back to the summary page
