# Supplementary material for: Tofu and fish oil independently modulate serum lipid profiles in rats: Analyses of 10 class lipoprotein profiles and the global hepatic transcriptome
Source: PLoS One. 2019 Jan 17;14(1):e0210950. doi: 10.1371/journal.pone.0210950 (PMC6336308; doi:10.1371/journal.pone.0210950)
Supplement: S3 Fig — (ZIP) [file pone.0210950.s003.zip › S3_Fig/Ch/LAC1.htm]

# LAC1

**ANOVA p-value**:0.0000001482   
  
Tukey multiple comparisons of means   
95% family-wise confidence level

| combinations | diff | lwr | upr | p adj |
| --- | --- | --- | --- | --- |
| 2-1 | -35.742757 | -54.72134 | -16.764172 | 0.0001302 |
| 3-1 | -44.226924 | -63.20551 | -25.248339 | 0.0000059 |
| 4-1 | -52.382518 | -70.75845 | -34.006582 | 0.0000002 |
| 3-2 | -8.484167 | -27.46275 | 10.494418 | 0.6144359 |
| 4-2 | -16.639761 | -35.01570 | 1.736175 | 0.0860026 |
| 4-3 | -8.155594 | -26.53153 | 10.220342 | 0.6198441 |

**Groups** 1: CS, 2: CF, 3: TS, 4: TF   
  
back to the summary page
