# Supplementary material for: Tofu and fish oil independently modulate serum lipid profiles in rats: Analyses of 10 class lipoprotein profiles and the global hepatic transcriptome
Source: PLoS One. 2019 Jan 17;14(1):e0210950. doi: 10.1371/journal.pone.0210950 (PMC6336308; doi:10.1371/journal.pone.0210950)
Supplement: S3 Fig — (ZIP) [file pone.0210950.s003.zip › S3_Fig/Ch/LAC2.htm]

# LAC2

**ANOVA p-value**:0.02531
  
  
Tukey multiple comparisons of means   
95% family-wise confidence level

| combinations | diff | lwr | upr | p adj |
| --- | --- | --- | --- | --- |
| 2-1 | -0.007864993 | -1.5798153 | 1.5640853 | 0.9999990 |
| 3-1 | 1.042209780 | -0.5297405 | 2.6141601 | 0.2862443 |
| 4-1 | -0.768535015 | -2.2905693 | 0.7534993 | 0.5176576 |
| 3-2 | 1.050074773 | -0.5218755 | 2.6220251 | 0.2801568 |
| 4-2 | -0.760670022 | -2.2827043 | 0.7613643 | 0.5261875 |
| 4-3 | -1.810744795 | -3.3327791 | -0.2887105 | 0.0153994 |

**Groups** 1: CS, 2: CF, 3: TS, 4: TF   
  
back to the summary page
