# Supplementary material for: Tofu and fish oil independently modulate serum lipid profiles in rats: Analyses of 10 class lipoprotein profiles and the global hepatic transcriptome
Source: PLoS One. 2019 Jan 17;14(1):e0210950. doi: 10.1371/journal.pone.0210950 (PMC6336308; doi:10.1371/journal.pone.0210950)
Supplement: S3 Fig — (ZIP) [file pone.0210950.s003.zip › S3_Fig/Ch/LDL1.htm]

# LDL1

**ANOVA p-value**:0.004074   
  
Tukey multiple comparisons of means   
95% family-wise confidence level

| combinations | diff | lwr | upr | p adj |
| --- | --- | --- | --- | --- |
| 2-1 | -6.4793488 | -14.801531 | 1.842834 | 0.1677399 |
| 3-1 | 5.5887679 | -2.733414 | 13.910950 | 0.2758983 |
| 4-1 | -0.6778477 | -8.735766 | 7.380071 | 0.9955198 |
| 3-2 | 12.0681167 | 3.745934 | 20.390299 | 0.0026921 |
| 4-2 | 5.8015010 | -2.256417 | 13.859419 | 0.2220788 |
| 4-3 | -6.2666157 | -14.324534 | 1.791303 | 0.1684609 |

**Groups** 1: CS, 2: CF, 3: TS, 4: TF   
  
back to the summary page
