# Supplementary material for: Tofu and fish oil independently modulate serum lipid profiles in rats: Analyses of 10 class lipoprotein profiles and the global hepatic transcriptome
Source: PLoS One. 2019 Jan 17;14(1):e0210950. doi: 10.1371/journal.pone.0210950 (PMC6336308; doi:10.1371/journal.pone.0210950)
Supplement: S3 Fig — (ZIP) [file pone.0210950.s003.zip › S3_Fig/Ch/LDL2.htm]

# LDL2

**ANOVA p-value**:0.0007101
  
  
Tukey multiple comparisons of means   
95% family-wise confidence level

| combinations | diff | lwr | upr | p adj |
| --- | --- | --- | --- | --- |
| 2-1 | -1.409207 | -12.11166 | 9.293244 | 0.9833642 |
| 3-1 | -4.193428 | -14.89588 | 6.509023 | 0.7059504 |
| 4-1 | -16.382163 | -26.74477 | -6.019559 | 0.0010872 |
| 3-2 | -2.784221 | -13.48667 | 7.918230 | 0.8899688 |
| 4-2 | -14.972956 | -25.33556 | -4.610352 | 0.0027903 |
| 4-3 | -12.188735 | -22.55134 | -1.826131 | 0.0167955 |

**Groups** 1: CS, 2: CF, 3: TS, 4: TF   
  
back to the summary page
