# Supplementary material for: Tofu and fish oil independently modulate serum lipid profiles in rats: Analyses of 10 class lipoprotein profiles and the global hepatic transcriptome
Source: PLoS One. 2019 Jan 17;14(1):e0210950. doi: 10.1371/journal.pone.0210950 (PMC6336308; doi:10.1371/journal.pone.0210950)
Supplement: S3 Fig — (ZIP) [file pone.0210950.s003.zip › S3_Fig/Ch/VLDL.htm]

# VLDL

**ANOVA p-value**: 0.03194
  
  
Tukey multiple comparisons of means   
95% family-wise confidence level

| combinations | diff | lwr | upr | p adj |
| --- | --- | --- | --- | --- |
| 2-1 | -1.720618 | -6.136175 | 2.6949399 | 0.7094212 |
| 3-1 | 1.324756 | -3.090802 | 5.7403133 | 0.8419807 |
| 4-1 | -3.363405 | -7.638750 | 0.9119403 | 0.1610929 |
| 3-2 | 3.045373 | -1.370184 | 7.4609311 | 0.2547782 |
| 4-2 | -1.642787 | -5.918133 | 2.6325580 | 0.7181449 |
| 4-3 | -4.688161 | -8.963506 | -0.4128154 | 0.0278096 |

**Groups** 1: CS, 2: CF, 3: TS, 4: TF   
  
back to the summary page
