# Supplementary material for: Tofu and fish oil independently modulate serum lipid profiles in rats: Analyses of 10 class lipoprotein profiles and the global hepatic transcriptome
Source: PLoS One. 2019 Jan 17;14(1):e0210950. doi: 10.1371/journal.pone.0210950 (PMC6336308; doi:10.1371/journal.pone.0210950)
Supplement: S4 Fig — (ZIP) [file pone.0210950.s004.zip › S4_Fig/TG/LAC1.htm]

# LAC1

**ANOVA p-value**:0.0000233   
  
Tukey multiple comparisons of means   
95% family-wise confidence level

| combinations | diff | lwr | upr | p adj |
| --- | --- | --- | --- | --- |
| 2-1 | -0.6115226 | -1.1040310 | -0.11901417 | 0.0109756 |
| 3-1 | -0.6443618 | -1.1368702 | -0.15185338 | 0.0070524 |
| 4-1 | -0.9904333 | -1.4673025 | -0.51356405 | 0.0000337 |
| 3-2 | -0.0328392 | -0.5253476 | 0.45966922 | 0.9977504 |
| 4-2 | -0.3789107 | -0.8557799 | 0.09795855 | 0.1548524 |
| 4-3 | -0.3460715 | -0.8229407 | 0.13079775 | 0.2162397 |

**Groups** 1: CS, 2: CF, 3: TS, 4: TF   
  
back to the summary page
