# Supplementary material for: Tofu and fish oil independently modulate serum lipid profiles in rats: Analyses of 10 class lipoprotein profiles and the global hepatic transcriptome
Source: PLoS One. 2019 Jan 17;14(1):e0210950. doi: 10.1371/journal.pone.0210950 (PMC6336308; doi:10.1371/journal.pone.0210950)
Supplement: S5 Fig — (ZIP) [file pone.0210950.s005.zip › S5_Fig/S5 Fig.htm]

# lipoproteins

Click image to enlarge. Click title for the test results.

## TG/Cholesterol

|  |  |  |  |  |
| --- | --- | --- | --- | --- |
| CM1 | CM2 | VLDL | LDL1 | LDL2 |
|  |  |  |  |  |
|  |
| LAC1 | LAC2 | mHDL | HDL1 | HDL2 |
|  |  |  |  |  |
