# Supplementary material for: Tofu and fish oil independently modulate serum lipid profiles in rats: Analyses of 10 class lipoprotein profiles and the global hepatic transcriptome
Source: PLoS One. 2019 Jan 17;14(1):e0210950. doi: 10.1371/journal.pone.0210950 (PMC6336308; doi:10.1371/journal.pone.0210950)
Supplement: S5 Fig — (ZIP) [file pone.0210950.s005.zip › S5_Fig/TG_cho/CM1.htm]

# CM1

**ANOVA p-value**: 0.03534
  
  
Tukey multiple comparisons of means   
95% family-wise confidence level

| combinations | diff | lwr | upr | p adj |
| --- | --- | --- | --- | --- |
| 2-1 | 0.03818860 | -0.114764460 | 0.1911417 | 0.9011659 |
| 3-1 | 0.15453676 | 0.001583706 | 0.3074898 | 0.0470198 |
| 4-1 | 0.11965473 | -0.028441431 | 0.2677509 | 0.1447069 |
| 3-2 | 0.11634817 | -0.036604890 | 0.2693012 | 0.1831086 |
| 4-2 | 0.08146613 | -0.066630026 | 0.2295623 | 0.4449835 |
| 4-3 | -0.03488203 | -0.182978192 | 0.1132141 | 0.9152959 |

**Groups** 1: CS, 2: CF, 3: TS, 4: TF   
  
back to the summary page
