# Supplementary material for: Tofu and fish oil independently modulate serum lipid profiles in rats: Analyses of 10 class lipoprotein profiles and the global hepatic transcriptome
Source: PLoS One. 2019 Jan 17;14(1):e0210950. doi: 10.1371/journal.pone.0210950 (PMC6336308; doi:10.1371/journal.pone.0210950)
Supplement: S5 Fig — (ZIP) [file pone.0210950.s005.zip › S5_Fig/TG_cho/LDL1.htm]

# LDL1

**ANOVA p-value**:0.04732
  
  
Tukey multiple comparisons of means   
95% family-wise confidence level

| combinations | diff | lwr | upr | p adj |
| --- | --- | --- | --- | --- |
| 2-1 | 0.01815434 | -0.1664471 | 0.20275578 | 0.9929001 |
| 3-1 | -0.13780869 | -0.3224101 | 0.04679274 | 0.1960265 |
| 4-1 | -0.12152954 | -0.3002691 | 0.05721003 | 0.2660062 |
| 3-2 | -0.15596303 | -0.3405645 | 0.02863840 | 0.1194710 |
| 4-2 | -0.13968388 | -0.3184234 | 0.03905569 | 0.1653210 |
| 4-3 | 0.01627916 | -0.1624604 | 0.19501872 | 0.9943359 |

**Groups** 1: CS, 2: CF, 3: TS, 4: TF   
  
back to the summary page
