# Supplementary material for: Tofu and fish oil independently modulate serum lipid profiles in rats: Analyses of 10 class lipoprotein profiles and the global hepatic transcriptome
Source: PLoS One. 2019 Jan 17;14(1):e0210950. doi: 10.1371/journal.pone.0210950 (PMC6336308; doi:10.1371/journal.pone.0210950)
Supplement: S5 Fig — (ZIP) [file pone.0210950.s005.zip › S5_Fig/TG_cho/LDL2.htm]

# LDL2

**ANOVA p-value**:0.5532
  
  
back to the summary page
