# Supplementary material for: Tofu and fish oil independently modulate serum lipid profiles in rats: Analyses of 10 class lipoprotein profiles and the global hepatic transcriptome
Source: PLoS One. 2019 Jan 17;14(1):e0210950. doi: 10.1371/journal.pone.0210950 (PMC6336308; doi:10.1371/journal.pone.0210950)
Supplement: S1 Table — (DOCX) [file pone.0210950.s006.docx]

| Fatty acids (weight %) | Soy oil | Fish oil |
| --- | --- | --- |
| C14:0 | 0.1 | 2.2 |
| C16:0 | 10.4 | 13.1 |
| C16:1 (*n*-7) | 0.3 | 6.9 |
| C18:0 | 3.9 | 3.0 |
| C18:1 (*n*-9) | 24.5 | 21.5 |
| C18:2 (*n*-6) | 52.1 | 2.1 |
| C18:3 (*n*-3) | 5.8 | 0.7 |
| C20:4 (*n*-6) | - | 3.0 |
| C20:5 (*n*-3) | - | 10.0 |
| C22:4 (*n*-6) | - | 0.7 |
| C22:5 (*n*-6) | - | 1.7 |
| C22:5 (*n*-3) | - | 2.6 |
| C22:6 (*n*-3) | - | 32.6 |
| *n*-3/*n*-6 ratio | 0.11 | 6.12 |

**S1 Table.** **Fatty acid compositions of dietary fat sources**

-, undetectable.
