# Supplementary material for: Tofu and fish oil independently modulate serum lipid profiles in rats: Analyses of 10 class lipoprotein profiles and the global hepatic transcriptome
Source: PLoS One. 2019 Jan 17;14(1):e0210950. doi: 10.1371/journal.pone.0210950 (PMC6336308; doi:10.1371/journal.pone.0210950)
Supplement: S2 Table — (DOCX) [file pone.0210950.s007.docx]

| Ingredients (g/kg diet) | CS | CF | TS | TF |
| --- | --- | --- | --- | --- |
| Casein | 225 | 225 | 60.9 | 60.9 |
| Freeze-dried tofu | 0 | 0 | 290.7 | 290.7 |
| Corn starch | 150 | 150 | 150 | 150 |
| Cellulose | 20 | 20 | 20 | 20 |
| Soybean oil | 150 | 100 | 50 | 0 |
| Fish oil | 0 | 50 | 0 | 50 |
| Mineral mix (AIN-93G)* | 35 | 35 | 35 | 35 |
| Vitamin mix (AIN-93)* | 10 | 10 | 10 | 10 |
| *L*-cystine | 3 | 3 | 3 | 3 |
| Chorine bitartrate | 2.5 | 2.5 | 2.5 | 2.5 |
| Sucrose | 404.5 | 404.5 | 377.9 | 377.9 |
| Total energy (kJ/kg diet) | 18099 | 18099 | 17594 | 17594 |

**S2 Table. Compositions of experimental diets.** CS, casein and soy oil diet; CF, casein and fish oil diet; TS, tofu and soy oil diet; TF, tofu and fish oil diet.

* composition recommended by the American Institute of Nutrition (1993).

Reference: Reeves PG, Nielsen FH, Fahey GC Jr. AIN-93 purified diets for laboratory rodents: final report of the American Institute of Nutrition ad hoc writing committee on the reformulation of the AIN-76A rodent diet. J Nutr. 1993;123(11):1939-51. doi: 10.1093/jn/123.11.1939. PMID: 8229312.
