# Supplementary material for: Tofu and fish oil independently modulate serum lipid profiles in rats: Analyses of 10 class lipoprotein profiles and the global hepatic transcriptome
Source: PLoS One. 2019 Jan 17;14(1):e0210950. doi: 10.1371/journal.pone.0210950 (PMC6336308; doi:10.1371/journal.pone.0210950)
Supplement: S4 Table — (DOCX) [file pone.0210950.s009.docx]

|  | CS | CF | TS | TF |
| --- | --- | --- | --- | --- |
| Food consumption (g/day) | 19.0 ± 1.1 | 20.4 ± 1.6 | 19.4 ± 1.7 | 18.8 ± 2.0 |
| Energy intake (kJ/day) | 344 ± 19 | 370 ± 29 | 342 ± 30 | 331 ± 35 |
| Final body weight (g) | 330 ± 18 | 349 ± 13 | 335 ± 25 | 322 ± 22 |

**S4 Table. Growth parameters of rats.** CS, casein and soy oil diet; CF, casein and fish oil diet; TS, tofu and soy oil diet; TF, tofu and fish oil diet. Data are shown as means ± SD, *n* =7-8 per group.
