# Supplementary material for: Tofu and fish oil independently modulate serum lipid profiles in rats: Analyses of 10 class lipoprotein profiles and the global hepatic transcriptome
Source: PLoS One. 2019 Jan 17;14(1):e0210950. doi: 10.1371/journal.pone.0210950 (PMC6336308; doi:10.1371/journal.pone.0210950)
Supplement: S5 Table — (DOCX) [file pone.0210950.s010.docx]

| Fatty acids (%) | CS | CF | TS | TF |
| --- | --- | --- | --- | --- |
| C14:0 | 0.782 ± 0.131 | 0.679 ± 0.111 | 0.512 ± 0.110 | 0.571 ± 0.117 |
| C16:0 | 27.5 ± 1.4 | 24.4 ± 3.9 | 23.4 ± 1.9 | 22.0 ± 2.0 |
| C16:1 (*ω*-7) | 2.76 ± 0.50 | 2.54 ± 0.56 | 0.934 ± 0.258 | 1.57 ± 0.24 |
| C18:0 | 2.34 ± 0.21 | 2.50 ± 0.17 | 2.11 ± 0.14 | 2.51 ± 0.46 |
| C18:1 (*ω*-9) | 28.1 ± 1.7 | 23.2 ± 2.5 | 19.1 ± 1.0 | 17.4 ± 0.8 |
| C18:2 (*ω*-6) | 31.8 ± 2.0 | 25.0 ± 1.8 | 42.0 ± 3.2 | 30.3 ± 1.6 |
| C18:3 (*ω*-6) | 0.328 ± 0.058 | 0.160 ± 0.022 | 0.251 ± 0.038 | 0.137 ± 0.020 |
| C18:3 (*ω*-3) | 1.48 ± 0.16 | 1.69 ± 0.23 | 2.85 ± 0.56 | 2.45 ± 0.25 |
| C20:3 (*ω*-6) | 0.188 ± 0.048 | 0.295 ± 0.054 | 0.376 ± 0.049 | 0.082 ± 0.008 |
| C20:4 (*ω*-6) | 0.801 ± 0.280 | 0.788 ± 0.099 | 0.984 ± 0.125 | 0.755 ± 0.068 |
| C20:5 (*ω*-3) | 0.088 ± 0.028 | 1.90 ± 0.72 | 0.237 ± 0.206 | 1.85 ± 0.51 |
| C22:5 (*ω*-6) | 0.362 ± 0.375 | 0.424 ± 0.221 | 0.792 ± 1.332 | 0.708 ± 0.269 |
| C22:5 (*ω*-3) | 0.215 ± 0.093 | 2.44 ± 0.63 | 0.773 ± 0.536 | 3.10 ± 0.84 |
| C22:6 (*ω*-3) | 0.354 ± 0.138 | 11.0 ± 3.9 | 1.48 ± 1.21 | 13.7 ± 2.3 |

**S5 Table. Fatty acid compositions of hepatic TG in rats.**

CS, casein and soy oil diet; CF, casein and fish oil diet; TS, tofu and soy oil diet; TF, tofu and fish oil diet. Data are shown as means ± SD, n = 7-8 per group.
