# Supplementary material for: Tofu and fish oil independently modulate serum lipid profiles in rats: Analyses of 10 class lipoprotein profiles and the global hepatic transcriptome
Source: PLoS One. 2019 Jan 17;14(1):e0210950. doi: 10.1371/journal.pone.0210950 (PMC6336308; doi:10.1371/journal.pone.0210950)
Supplement: S8 Table — (DOCX) [file pone.0210950.s013.docx]

|  | CS | CF | TS | TF |
| --- | --- | --- | --- | --- |
| Fatty acid synthesis (nmol/min per mg protein) | | | | |
| Fatty acid synthase | 13.5 ± 4.9^b^ | 4.77 ± 3.54^a^ | 1.80 ± 1.53^a^ | 0.741 ± 1.296^a^ |
| ATP-citrate lyase | 49.9 ± 10.3^c^ | 31.2 ± 10.0^b^ | 19.0 ± 6.4^a^ | 9.29 ± 2.4^a^ |
| Glucose 6-phospate dehydrogenase | 50.9 ±11.3^c^ | 27.7 ± 5.8^b^ | 14.8 ± 3.9^a^ | 7.99 ± 2.0^a^ |
| 6-phosphogluconate dehydrogenase | 70.9 ± 13.2^b^ | 58.9 ± 8.2^b^ | 41.6 ± 6.9^a^ | 32.9 ± 3.7^a^ |
| Malic enzyme | 51.6 ± 12.6^b^ | 64.0 ± 16.4^b^ | 18.8 ± 4.2^a^ | 24.8 ± 6.1^a^ |
| Pyruvate kinase | 289 ± 51^c^ | 171 ± 63^b^ | 163 ± 39^a^ | 67.2 ± 59.6^a^ |
| Fatty acid oxidation (nmol/min per mg protein) | | | | |
| Peroxisomal fatty oxidation | 1.92 ± 0.39^a^ | 3.59 ± 0.40^b^ | 1.56 ± 0.36^a^ | 3.26 ± 0.38^b^ |
| Acyl-CoA oxidase | 1.58 ± 0.17^a^ | 3.32 ± 0.35^b^ | 1.55 ± 0.37^a^ | 3.91 ± 0.45^c^ |
| Carnitine palmitoyltransferase | 4.01 ± 0.55^a^ | 7.02 ± 1.04^b^ | 3.65 ± 0.59^a^ | 6.26 ± 0.84^b^ |
| Enoly-CoA hydratase | 5864 ± 567^a^ | 6387 ± 11.6^b^ | 6086 ± 1035^a^ | 6648 ± 723^b^ |
| 3-Hydroxyacyl-CoA dehydrogenase  (Reverse reaction) | 50.0 ± 8.7^a^ | 68.6 ± 11.6^a^ | 100 ± 32.6^b^ | 97.6 ± 13.7^b^ |
| 3-Hydroxyacyl-CoA dehydrogenase  (Forward reaction) | 7.56 ± 1.07^a^ | 5.93 ± 1.78^a^ | 19.6 ± 9.17^b^ | 9.65 ± 2.77^a^ |
| 3-Ketoacyl-CoA thiolase | 189 ± 26^a^ | 279 ± 39^b^ | 177 ± 37^a^ | 262 ± 38^b^ |
| 2,4-Dienoyl-CoA reductase | 2.20 ± 0.35^a^ | 2.93 ± 0.48^ab^ | 2.51 ± 0.47^a^ | 3.44 ± 0.66^b^ |

**S8 Table. Activities of enzymes involved in hepatic fatty acid synthesis and oxidation.**

CS, casein and soy oil diet; CF, casein and fish oil diet; TS, tofu and soy oil diet; TF, tofu and fish oil diet. Data are shown as means ± SD, n = 7-8 per group. ^abc^Means without a common letter significantly differ, *p* < 0.05.
