# Supplementary material for: Tofu and fish oil independently modulate serum lipid profiles in rats: Analyses of 10 class lipoprotein profiles and the global hepatic transcriptome
Source: PLoS One. 2019 Jan 17;14(1):e0210950. doi: 10.1371/journal.pone.0210950 (PMC6336308; doi:10.1371/journal.pone.0210950)
Supplement: S1 Appendix — (ZIP) [file pone.0210950.s014.zip › S1 Appendix/S1 Appendix.htm]

p


# Statistical tests

## Fig. 3

### Total Cholesterol

**ANOVA p-value**: 1.961e-06   
  
Tukey-Kramer multiple comparisons of means  
95% family-wise confidence level

|  |  |  |  |  |
| --- | --- | --- | --- | --- |
|  | difference | lower | upper | p-value |
| CF-CS | -21.061429 | -34.47646 | -7.6463954 | 0.0011731 |
| TS-CS | -19.040000 | -32.45503 | -5.6249668 | 0.0033259 |
| TF-CS | -34.571071 | -47.56012 | -21.5820214 | 0.0000007 |
| TS-CF | 2.021429 | -11.39360 | 15.4364618 | 0.9754810 |
| TF-CF | -13.509643 | -26.49869 | -0.5205928 | 0.0393354 |
| TF-TS | -15.531071 | -28.52012 | -2.5420214 | 0.0148116 |

 

### Total TG

**ANOVA p-value**: 0.04076   
  
Tukey-Kramer multiple comparisons of means  
95% family-wise confidence level

|  |  |  |  |  |
| --- | --- | --- | --- | --- |
|  | difference | lower | upper | p-value |
| CF-CS | -13.584286 | -48.58317 | 21.4145959 | 0.7118989 |
| TS-CS | -4.578571 | -39.57745 | 30.4203102 | 0.9836732 |
| TF-CS | -34.727679 | -68.61520 | -0.8401572 | 0.0431318 |
| TS-CF | 9.005714 | -25.99317 | 44.0045959 | 0.8930588 |
| TF-CF | -21.143393 | -55.03091 | 12.7441285 | 0.3367112 |
| TF-TS | -30.149107 | -64.03663 | 3.7384142 | 0.0938572 |

 

## Fig. 4

### Time VLDL

**ANOVA p-value**: 6.365e-05   
  
Tukey-Kramer multiple comparisons of means  
95% family-wise confidence level

|  |  |  |  |  |
| --- | --- | --- | --- | --- |
|  | difference | lower | upper | p-value |
| CF-CS | 0.16737864 | -0.03891216 | 0.3736694 | 0.1422006 |
| TS-CS | 0.26206037 | 0.05576957 | 0.4683512 | 0.0090822 |
| TF-CS | 0.41611112 | 0.21637091 | 0.6158513 | 0.0000322 |
| TS-CF | 0.09468173 | -0.11160907 | 0.3009725 | 0.594376 |
| TF-CF | 0.24873248 | 0.04899228 | 0.4484727 | 0.0107167 |
| TF-TS | 0.15405075 | -0.04568946 | 0.353791 | 0.1738935 |

 

### Time LDL1

**ANOVA p-value**: 4.55e-05   
  
Tukey-Kramer multiple comparisons of means  
95% family-wise confidence level

|  |  |  |  |  |
| --- | --- | --- | --- | --- |
|  | difference | lower | upper | p-value |
| CF-CS | -0.053503356 | -0.14445871 | 0.037452 | 0.3871108 |
| TS-CS | -0.155510723 | -0.24646608 | -0.06455537 | 0.0004409 |
| TF-CS | -0.163317844 | -0.25138499 | -0.0752507 | 0.0001595 |
| TS-CF | -0.102007367 | -0.19296272 | -0.01105201 | 0.023789 |
| TF-CF | -0.109814488 | -0.19788163 | -0.02174734 | 0.0106002 |
| TF-TS | -0.007807121 | -0.09587427 | 0.08026002 | 0.9947697 |

 

### Time LDL2

**ANOVA p-value**: 0.02022   
  
Tukey-Kramer multiple comparisons of means  
95% family-wise confidence level

|  |  |  |  |  |
| --- | --- | --- | --- | --- |
|  | difference | lower | upper | p-value |
| CF-CS | 0.01505972 | -0.031861085 | 0.06198052 | 0.8136878 |
| TS-CS | 0.05559229 | 0.008671488 | 0.10251309 | 0.0158918 |
| TF-CS | 0.03176666 | -0.013664206 | 0.07719754 | 0.2441331 |
| TS-CF | 0.04053257 | -0.006388228 | 0.08745337 | 0.1079817 |
| TF-CF | 0.01670695 | -0.028723922 | 0.06213782 | 0.7442523 |
| TF-TS | -0.02382562 | -0.069256495 | 0.02160525 | 0.4858201 |

 

## Fig. 5

### TG log(CM1/CM2)

**ANOVA p-value**: 0.00492   
  
Tukey-Kramer multiple comparisons of means  
95% family-wise confidence level

|  |  |  |  |  |
| --- | --- | --- | --- | --- |
|  | difference | lower | upper | p-value |
| CF-CS | 0.00694022 | -0.09277053 | 0.106650973 | 0.9974442 |
| TS-CS | 0.12810266 | 0.0283919 | 0.227813409 | 0.0082542 |
| TF-CS | 0.02241746 | -0.07412706 | 0.118961982 | 0.9184635 |
| TS-CF | 0.12116244 | 0.02145168 | 0.220873189 | 0.0130549 |
| TF-CF | 0.01547724 | -0.08106728 | 0.112021762 | 0.9707476 |
| TF-TS | -0.1056852 | -0.20222972 | -0.009140674 | 0.0281366 |

 

## Fig. 6

### Cholesterol LAC1

**ANOVA p-value**: 2.214e-07   
  
Tukey-Kramer multiple comparisons of means  
95% family-wise confidence level

|  |  |  |  |  |
| --- | --- | --- | --- | --- |
|  | difference | lower | upper | p-value |
| CF-CS | -35.742757 | -54.72134 | -16.764172 | 0.0001302 |
| TS-CS | -44.226924 | -63.20551 | -25.248339 | 0.0000059 |
| TF-CS | -52.382518 | -70.75845 | -34.006582 | 0.0000002 |
| TS-CF | -8.484167 | -27.46275 | 10.494418 | 0.6144359 |
| TF-CF | -16.639761 | -35.0157 | 1.736175 | 0.0860026 |
| TF-TS | -8.155594 | -26.53153 | 10.220342 | 0.6198441 |

 

## Histogram of the p-values

Distribution of the p-values showed that the
prior probabilities to the null hypotheses should be quite low. The p-values
were obtained by the one-way ANOVA; the null hypothesis *H*0 was that the groups’
population means were identical. According to the Bayes’ theorem,
the probability of false positive errors can be described as ,
where  is the p-value and  is the prior probabilities to the null
hypotheses. If  is high, the p-value will take random
values hence distribution of the p-value will become uniform, while if  is low, the distribution will be heavily
skewed, such as seen in this case. When  is low enough, the  will also become low. In such cases, the
problems provoked by the multiplicity of
tests, such as pointed in the family-wise error
rate, will not occur, because the problems are estimated on an assumption that
the false positive errors will occur as the same level of the p-values.
